# Efficacy and safety of pembrolizumab in recurrent/metastatic head and neck squamous cell carcinoma: pooled analyses after long-term follow-up in KEYNOTE-012

**DOI:** 10.1038/s41416-018-0131-9

**Published:** 2018-06-29

**Authors:** Ranee Mehra, Tanguy Y. Seiwert, Shilpa Gupta, Jared Weiss, Iris Gluck, Joseph P. Eder, Barbara Burtness, Makoto Tahara, Bhumsuk Keam, Hyunseok Kang, Kei Muro, Ravit Geva, Hyun Cheol Chung, Chia-Chi Lin, Deepti Aurora-Garg, Archana Ray, Kumudu Pathiraja, Jonathan Cheng, Laura Q. M. Chow, Robert Haddad

**Affiliations:** 1 Fox Chase Cancer Center, Philadelphia, PA USA; 2 University of Chicago, Chicago, IL USA; 3 H. Lee Moffitt Cancer Center & Research Institute, Tampa, FL USA; 4 Lineberger Comprehensive Cancer Center at the University of North Carolina, Chapel Hill, NC USA; 5 Sheba Medical Center, Ramat Gan, Israel; 6 Yale Cancer Center, New Haven, CT USA; 7 National Cancer Center Hospital East, Chiba, Japan; 8 Seoul National University Hospital, Seoul, Republic of Korea; 9 Johns Hopkins University, Baltimore, MD USA; 10 Aichi Cancer Center Hospital, Nagoya, Japan; 11 Sourasky Medical Center, Tel Aviv, Israel; 12 Yonsei Cancer Center, Yonsei University College of Medicine, Seoul, Republic of Korea; 13 National Taiwan University Hospital, Taipei, Taiwan; 14 Merck & Co., Inc, Kenilworth, NJ USA; 15 University of Washington, Seattle, WA USA; 16 Dana-Farber Cancer Institute, Boston, MA USA; 17 Present Address: Johns Hopkins University, Baltimore, MD USA; 18 Present Address: Masonic Cancer Centre, University of Minnesota, Minneapolis, MN USA; 19 Present Address: Yale Cancer Center, New Haven, CT USA

**Keywords:** Oncology, Head and neck cancer

## Abstract

**Background:**

Second-line treatment options for advanced head and neck squamous cell carcinoma (HNSCC) are limited. The phase Ib KEYNOTE-012 study evaluated the safety and the efficacy of pembrolizumab for the treatment of HNSCC after long-term follow-up.

**Methods:**

Multi-centre, non-randomised trial included two HNSCC cohorts (initial and expansion) in which 192 patients were eligible. Patients received pembrolizumab 10 mg/kg every 2 weeks (initial cohort; *N* = 60) or 200 mg every 3 weeks (expansion cohort; *N* = 132). Co-primary endpoints were safety and overall response rate (ORR; RECIST v1.1; central imaging vendor review).

**Results:**

Median follow-up was 9 months (range, 0.2–32). Treatment-related adverse events (AEs) of any grade and grade 3/4 occurred in 123 (64%) and 24 (13%) patients, respectively. No deaths were attributed to treatment-related AEs. ORR was 18% (34/192; 95% CI, 13–24%). Median response duration was not reached (range, 2+ to 30+ months); 85% of responses lasted ≥6 months. Overall survival at 12 months was 38%.

**Conclusions:**

Some patients received 2 years of treatment and the responses were ongoing for more than 30 months; the durable anti-tumour activity and tolerable safety profile, observed with long-term follow-up, support the use of pembrolizumab as a treatment for recurrent/metastatic HNSCC.

## INTRODUCTION

More than 500,000 new cases of head and neck squamous cell carcinoma (HNSCC) are diagnosed each year.^1^ Most patients present with locally advanced disease, which is most often managed using a multi-method approach that combines surgery, chemotherapy, and radiation therapy.^2^ With disease recurrence or metastatic disease, the standard first-line treatment is the combination of cetuximab, platinum, and fluorouracil (i.e., EXTREME regimen).^3^ Historically, treatment options were limited for patients with advanced HNSCC that progressed after first-line therapy;^3^ however, recent results from clinical trials with immune checkpoint inhibitors have shown promising activity for second-line therapy.^4–7^

The programmed death 1 (PD-1) pathway is an important immune checkpoint exploited by immunosuppressive cancers including HNSCC to avoid immune detection.^8^ Binding of PD-1 by either of its ligands, PD-L1 or PD-L2, suppresses the activation of effector T cells.^9–11^ Although this interaction functions to protect against excessive inflammation under normal conditions, it is hypothesised that upregulation of the PD-1 pathway allows cancer cells to develop adaptive immune resistance.^12^ Both PD-L1 and PD-L2 expression have been reported in HNSCC,^13,14^ and the PD-1 pathway has been established as an effective target in HNSCC.

Pembrolizumab, an anti-PD-1 antibody, has demonstrated robust anti-tumour activity and a manageable safety profile in multiple tumour types, and is currently approved in more than 60 countries for one or more advanced malignancies.^15^ Based on the safety and efficacy observed in patients with HNSCC enrolled in the phase Ib KEYNOTE-012 trial (clinicaltrials.gov identifier: NCT01848834), pembrolizumab was approved by the US Food and Drug Administration for recurrent or metastatic HNSCC that has progressed on or after platinum-containing chemotherapy.^15^ Specifically, the KEYNOTE-012 trial enrolled two HNSCC cohorts: an initial cohort (*N* = 60) and an expansion cohort (*N* = 132). After a median follow-up of 14 months (interquartile range [IQR], 4–14 months), the confirmed overall response rate (ORR) in the initial cohort was 18% (95% CI, 8–32%); responses lasted a median of 53 weeks (95% CI, 13 weeks to not reached).^6^ An ORR of 18% (95% CI, 12–26%) was also reported in the expansion cohort after a median follow-up of 9 months (IQR, 3–11 months); the median duration of response in this cohort was not reached at the time of reporting.^5^ Pembrolizumab was well tolerated in both cohorts: 17% and 9% of patients experienced grade 3/4 treatment-related adverse events (AEs) in the initial and the expansion cohorts, respectively.

Herein we report long-term results of patients in the two HNSCC cohorts of the multi-centre, non-randomised, phase Ib KEYNOTE-012 trial, which investigated the safety and the efficacy of pembrolizumab in patients with advanced solid tumours.^5,6^ Because the frequency of response was similar across the two cohorts, data from patients in the initial and expansion cohorts were pooled for these analyses.

## METHODS

### Patients

Detailed eligibility criteria for the individual HNSCC cohorts have been published.^5,6^ Key inclusion criteria applicable to both cohorts included ≥18 years of age; histologically or cytologically confirmed HNSCC; recurrent, metastatic, or persistent disease; measurable disease as per the Response Evaluation Criteria in Solid Tumors, version 1.1 (RECIST v1.1); and Eastern Cooperative Oncology Group performance status (ECOG PS), 0 or 1. Only the initial cohort required evidence of PD-L1 expression. There was no limit to the number of prior therapies; however, prior treatment with immune checkpoint inhibitors was not allowed. Prior immunosuppressive therapy, chemotherapy, and therapy with anti-cancer monoclonal antibodies had to be concluded within 7 days, 2 weeks, and 4 weeks, respectively, before the start of the study treatment. The original studies were conducted in accordance with Good Clinical Practice guidelines and the Declaration of Helsinki, and the study protocol was approved by the institutional review boards or ethics committees of all participating sites. All patients provided written informed consent before study entry.

### Study design

Pembrolizumab dose and administration schedule differed between the HNSCC cohorts.^5,6^ Patients in the initial cohort received pembrolizumab 10 mg/kg every 2 weeks; patients in the expansion cohort received pembrolizumab 200 mg every 3 weeks. A lower dose and less frequent administration schedule was chosen for the expansion cohort based on data from the other pembrolizumab trials and pharmacodynamic modeling, which indicated that a lower dose and less frequent administration schedule were sufficient for target engagement and clinical activity.^16,17^ For both cohorts, treatment continued until confirmed disease progression, unacceptable toxicity, investigator’s or patient’s decision to withdraw, or completion of 24 months of treatment.

Tumour response was evaluated every 8 weeks using computed tomography or magnetic resonance imaging assessed per RECIST v1.1 by central imaging vendor review. Patients who experienced confirmed complete response (CR) could discontinue pembrolizumab if they received at least 24 weeks of treatment. If imaging indicated disease progression, the progression was to be substantiated by subsequent imaging performed no sooner than 4 weeks later. Clinically stable patients could remain on treatment during that time. If subsequent imaging indicated a reduction in tumour burden from what was seen with the initial imaging, the patient could continue treatment as scheduled.

AEs were monitored throughout the trial and for 30 days after the end of the treatment. Serious AEs and immune-mediated AEs (imAE), defined as events with potential drug-related immunologic causes, regardless of attribution by the investigator, were monitored for 90 days after the treatment was ended. AEs were graded using the National Cancer Institute Common Terminology Criteria for Adverse Events, version 4.0. Pembrolizumab was withheld for most grade 3 treatment-related AEs, until toxicity was resolved to grade 0/1. If toxicity did not resolve within 12 weeks of the last pembrolizumab dose, the treatment was discontinued. Treatment was also discontinued for grade 4 treatment-related AEs and for recurrent grade 3 treatment-related AEs.

The co-primary endpoints were safety and ORR (RECIST v1.1, central imaging vendor review). Secondary endpoints included ORR (RECIST v1.1, investigator review), ORR (RECIST v1.1, central imaging vendor review) in patients previously treated with cetuximab and platinum, progression-free survival (PFS), overall survival (OS), and duration of response (DOR).

### Tumour analysis

Patients were required to provide the archival tissue samples or the newly obtained core or excisional tumour biopsy samples for human papillomavirus (HPV) and biomarker analyses. Patients were eligible, regardless of the HPV status. Patients were classified as having HPV-associated disease if the primary location of their tumour was in the oropharynx and the site investigator considered the tumour to be HPV positive. HPV-negative tumours determined by the individual study site  and/or patients with primary tumour locations outside of the oropharynx were classified as non-HPV-associated disease.

PD-L1 expression status during screening was determined using a prototype PD-L1 immunohistochemical assay^18^ performed at a laboratory site (QualTek) accredited by the College of American Pathologists and Clinical Laboratory Improvement Amendments, and used commercially available reagents from the EnVision FLEX+ HRP-Polymer kit (DAKO K8012; Agilent Technologies) and the anti-PD-L1 (clone 22C3) antibody (Merck & Co., Inc.). Detailed methods for this assay have been described elsewhere.^18^

In a separate analysis of PD-L1 expression and anti-tumour response, PD-L1 expression was retrospectively evaluated using an investigational version of the PD-L1 IHC 22C3 pharmDx assay (Agilent Technologies). The staining protocol was performed according to the instructions of the commercial assay.^19,20^ The expression was scored using two methods: tumour proportion score (TPS) and combined positive score (CPS). TPS was defined as the percentage of tumour cells with membranous PD-L1 expression. CPS was defined the number of PD-L1-positive cells [tumour cells, lymphocytes, and macrophages] divided by the total number of tumour cells times 100. Both scores ranged from 0 to 100; a cut-off of ≥1 was used to define the PD-L1 expression.

Similarly, PD-L2 expression was retrospectively determined by immunohistochemistry using the anti-PD-L2 (clone 3G2) antibody (Merck & Co., Inc.). PD-L2 expression was scored by determining the percentage of PD-L2–positive cells (tumour cells, macrophages, lymphocyte) over the total tumour cells. Scores ranged from 0 to 100%; a 1% cut-off was used to define the PD-L2 expression.

### Statistical analysis

In this pooled analysis, the efficacy and the safety were assessed in all patients with HNSCC who received at least one dose of pembrolizumab (all-patients-as-treated population). Efficacy endpoints were also analysed by subgroups based on the HPV status, biomarker expression, and prior therapies (platinum, platinum, and cetuximab [treatments could be concurrent or subsequent]).

ORR was defined as the proportion of patients in the analysis population who experienced confirmed CR or partial response (PR). The response rates, point estimates, and 95% CI were determined using the exact binomial distribution. PFS was defined as the time from the first dose to the first instance of documented disease progression or death from any cause, whichever occurred first. OS was defined as the time from the first dose to death. DOR was defined as the time from the first confirmed response to disease progression. Kaplan–Meier statistics were used to estimate PFS, OS, and DOR. Patients with missing response data were considered non-responders; non-responders were excluded from the DOR analyses. Patients with missing survival data were censored at their last assessment.

Logistic (ORR) or Cox (PFS and OS) proportional hazards regression one-sided testing was performed to assess the relationship between efficacy and PD-L1 or PD-L2 expression.

## RESULTS

### Patients

A total of 192 patients with HNSCC from 16 centres in five countries were enrolled and received at least one dose of pembrolizumab, including 60 patients in the initial cohort and 132 patients in the expansion cohort. First patient was enrolled in 7 June, 2013, and the last patient was enrolled in 8 October, 2014. Therefore, the data cut-off for the current analysis (26 April, 2016) was more than 18 months after the last patient started the study.

Patient median age was 60 years (range, 20–84 years); 83% were men (Table 1). The majority of the patients were heavily pre-treated: 74% received at least two prior lines of systemic therapy. Specifically, 91% of patients had received a platinum-based regimen, and 57% had received prior platinum and prior cetuximab treatment. Seventy-seven percent of patients had non-HPV-associated disease and 23% had HPV-associated disease.

**Table 1:** Baseline demographics and characteristics (all-patients-as-treated population)

Characteristic	*N* = 192
Age, median (range) (y)	60 (20–84)
Male	159 (83)
Race	
White	147 (77)
Asian	29 (15)
Other	16 (8)
ECOG performance status	
0	57 (30)
1	135 (70)
Smoking status	
Current/former	140 (73)
Never	52 (27)
HPV status	
Associated	45 (23)
Not associated	147 (77)
Prior radiation	146 (76)
Prior surgery	129 (67)
Prior adjuvant and/or neoadjuvant therapy,^a^ *n*	90 (47)
No. of prior lines of systemic therapies, median (range), *n*	2 (0–7)
No. of previous lines of systemic therapy^b^	
1	47 (24)
2	56 (29)
≥3	86 (45)
Prior platinum therapy	174 (91)
Prior platinum and cetuximab therapy	110 (57)
Metastatic stage	
MX	1 (<1)
M0	26 (14)
M1	165 (86)

The data are no. (%) unless otherwise stated.

*ECOG* Eastern Cooperative Oncology Group, *HPV* human papillomavirus.

^a^All adjuvant/neoadjuvant therapies were chemotherapies.

^b^Three patients had 0 lines of systemic therapy

**Fig. 1:**
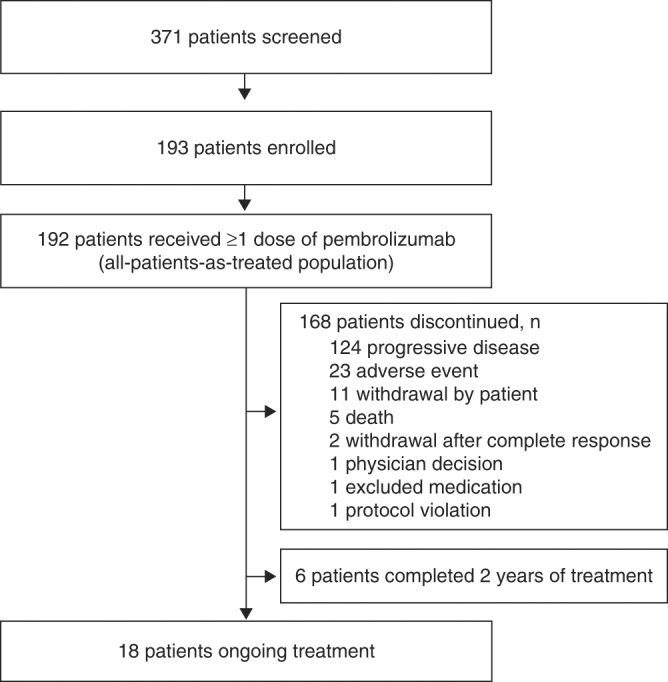
Patient disposition

The median follow-up, as of 26 April, 2016, was 9 months (range, 0.2–32 months). By that time, 168 (88%) patients had discontinued the treatment, most commonly for progressive disease (*n* = 124) or an AE (*n* = 23), 18 (9%) patients remained on the treatment, and 6 (3%) patients had completed 2 years of pembrolizumab (Fig. 1).

### Safety

At data cut-off, the median time on pembrolizumab was 14 weeks (range, 0.1–107 weeks). Treatment-related AEs occurred in 64% (*n* = 123) of patients (Table 2). Thirteen percent (*n* = 24) experienced a treatment-related AE of grade 3/4 severity; increases in the alanine aminotransferase and the aspartate aminotransferase levels were the only grade 3/4 treatment-related AEs that occurred in more than two patients. There were 12 discontinuations (6% of patients) and zero deaths attributed to treatment-related AEs. Immune-mediated adverse events (imAEs) and infusion reactions, regardless of the attribution by the investigator, occurred in 24% (*n* = 46) of patients; the only imAEs that occurred in more than two patients were hypothyroidism (grade 1/2, *n* = 26; grade 3, *n* = 2), pneumonitis (grade 1/2, *n* = 3; grade 3, *n* = 2), adrenal insufficiency (grade 1/2, n = 2), and thyroiditis (grade 1/2, *n* = 3). All patients experiencing hypothyroidism received prior radiation. One grade 4 imAE of diabetic ketoacidosis was reported, as was one grade 3 imAE of each of the following: type 1 diabetes mellitus, decubitus ulcer, papule, rash, colitis, drug-induced liver injury, and macular rash. Two treatment-related cardiac events were reported in the same patient (atrial fibrillation and congestive heart failure; both grade 3) (Table 2).

**Table 2:** Treatment-related adverse events (all-patients-as-treated population; *N* = 192)

Treatment-related adverse event	Any grade occurring in ≥2% of patients (No. (%))
Any	123 (64)
Fatigue	42 (22)
Hypothyroidism	19 (10)
Rash	18 (9)
Pruritus	16 (8)
Appetite decrease	16 (8)
Pyrexia	12 (6)
Nausea	11 (6)
Arthralgia	10 (5)
Dry skin	9 (5)
Weight decrease	9 (5)
AST level increase	6 (3)
Facial swelling	6 (3)
Anaemia	8 (4)
ALT level increase	5 (3)
Myalgia	5 (3)
Diarrhoea	5 (3)
Pneumonitis	5 (3)
Stomatitis	4 (2)
Vomiting	4 (2)
Chills	4 (2)
Blood TSH level increase	4 (2)
Hyponatremia	4 (2)
Maculopapular rash	4 (2)
	*Grade 3/4 occurring in ≥2 patients* (No. (%))
Any	24 (13)
ALT level increase	3 (2)
AST level increase	3 (2)
Hypothyroidism	2 (1)
Fatigue	2 (1)
Appetite decrease	2 (1)
Hyponatremia	2 (1)
Pneumonitis	2 (1)
Facial swelling	2(1)
	*Rare events of interest* (No. (%) [grade])
Immune-mediated	
Adrenal insufficiency	2 (1) [1, 2]
Colitis	1 (1) [3]
Diabetic ketoacidosis	1 (1) [4]
Type 1 diabetes mellitus	1 (1) [3]
Cardiac	
Atrial fibrillation	1 (1) [3]
Congestive heart failure	1 (1) [3]

*ALT* alanine aminotransferase, *AST* aspartate aminotransferase, *TSH* thyroid stimulating hormone

### Efficacy

**Fig. 2:**
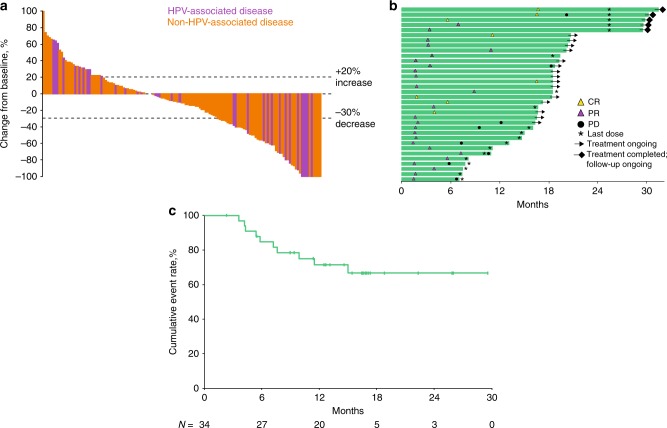
Tumour response to pembrolizumab according to RECIST v1.1 by central imaging vendor review. **a** Best percentage change from baseline in target lesions (*n* = 139). Includes patients who had measurable disease at baseline and at least one post baseline scan. **b** Treatment exposure and duration of response in patients achieving partial responses or complete responses (*n* = 34). **c** Kaplan–Meier estimate of the duration of response in patients achieving partial responses or complete responses. RECIST Response Evaluation Criteria in Solid Tumors

ORR across all patients was 18% (95% CI, 13–24%) (Table 3). Eight (4%) patients experienced CR and 26 (14%) patients experienced PR. Another 33 (17%) patients experienced stable disease (SD) and 93 (48%) patients had progressive disease as the best response. Clinical benefit rate, defined as the proportion of patients experiencing CR, PR, or SD for ≥6 months, was 20% (95% CI, 15–27%). Similar response rates were reported when analyses were restricted to patients whose disease progressed after prior platinum therapy (17% [95% CI, 12–23%]), or prior platinum and prior cetuximab therapy (15% [95% CI, 9–23%]) (Supplemental Table 1). ORR was 24% (95% CI, 13–40%) among patients with HPV-associated disease and 16% (95% CI, 10–23) among those with non-HPV-associated disease (Table 3). Decrease in the target lesion size from baseline was observed in 60% of all patients, including 57% with HPV-associated disease and 62% with non-HPV-associated disease (Fig. 2a).

**Table 3:** Tumour response to pembrolizumab as per RECIST v1.1 by central imaging vendor review (all-patients-as-treated population; *N* = 192)

	All *N* = 192		HPV associated *n* = 45		Non-HPV associated *n* = 147	
	No.	% (95% CI)	No.	% (95% CI)	No.	% (95% CI)
Overall response rate	34	18 (13–24)	11	24 (13–40)	23	16 (10–23)
Complete response	8	4 (2–8)	4	9 (3–21)	4	3 (1–7)
Partial response	26	14 (9–19)	7	16 (7–30)	19	13 (8–19)
Stable disease	33	17 (12–23)	7	16 (7–30)	26	18 (12–25)
Progressive disease	93	48 (41–56)	19	42 (28–58)	74	50 (42–59)
Non-CR/Non-PD	7	4 (2–7)	1	2 (0.1–12)	6	4 (2–9)
No assessment	21	11 (7–16)	6	13 (5–27)	15	10 (6–16)
Not evaluable	4	2 (0.6–5)	1	2 (0.1–12)	3	2 (0.4–6)

Only confirmed responses are included.

*CR* complete response, *PD* progressive disease, *RECIST* Response Evaluation Criteria in Solid Tumours.

No assessment: patient discontinued before the first imaging assessment (reasons: progressive disease [*n* = 12]; adverse event [*n* = 3]; withdrawal by patient [*n* = 3]; death [*n* = 2]; protocol violation [*n* = 1]).

Not evaluable: patient had post baseline imaging, but images were not of sufficient quality to determine response

Among the 34 responders, the median time to response was 2 months (range, 2–17 months) (Fig. 2b). Median DOR was not reached (range, 2+ to 30+ months) (Fig. 2c; Supplemental Table 2). Based on Kaplan–Meier estimates, 85% of responses lasted ≥6 months and 71% of responses lasted ≥12 months, and 65% of responses were ongoing at the data cut-off with three lasting at least 2 years. Among those patients who experienced response, 15 were still receiving pembrolizumab, 2 discontinued pembrolizumab after experiencing CR, and 5 completed the study after receiving pembrolizumab for 2 years.

**Fig. 3:**
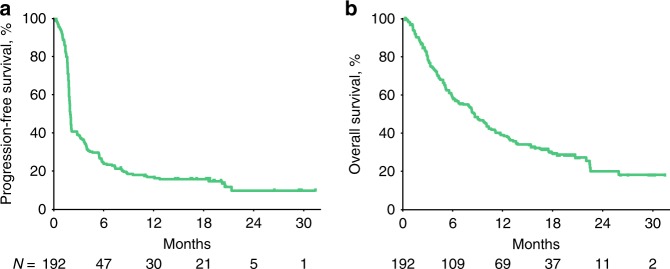
Survival in patients treated with pembrolizumab. Kaplan–Meier estimates of **a** progression-free survival per RECIST v1.1 by central imaging vendor review and **b** overall survival (all-patients-as-treated population). RECIST Response Evaluation Criteria in Solid Tumors

Median PFS was 2.1 months (95% CI, 1.9–2.1 months) (Fig. 3a). PFS rates at 6 and 12 months were 25% and 17%, respectively. Median OS was 8 months (95% CI, 6–10 months) (Fig. 3b). The 6-month OS rate was 58%, and the 12-month OS rate was 38%.

### Biomarker analysis

Efficacy and PD-L1 expression data were available for 188 patients. When PD-L1 expression was determined using TPS, 123 (65%) patients had PD-L1-expressing tumours, whereas 65 (35%) patients had tumours that did not express PD-L1. When PD-L1 expression was determined using CPS, 152 (81%) patients had PD-L1-expressing tumours and 36 (19%) patients had non-PD-L1-expressing tumours. Significantly, higher response rates were observed in patients with vs. without PD-L1 expression using CPS (21 vs. 6%; one-sided *P* = 0.023), but not TPS (Supplemental Table 3). Similarly, the median (95% CI) PFS rates were significantly different using CPS (PD-L1–expressing, 2.1 months [1.9–3.2 months]; non-PD-L1-expressing, 2.0 months [1.7–2.2 months]; one-sided *P* = 0.026), but not TPS (Supplemental Fig. 1A). The median (95% CI) OS rates were also significantly different when CPS was used (PD-L1-expressing, 10 months [9–13 months]; non-PD-L1-expressing, 5 months [3–8 months]; one-sided *P* = 0.008) (Supplemental Fig. 1B).

PD-L2 expression data were available for 172 patients; 111 (65%) patients had PD-L2–expressing tumours and 61 (35%) patients had non-PD-L2-expressing tumours. A significant positive correlation was observed between PD-L1 and PD-L2 expression (one-sided *P* < 0.001). A significantly higher response rate was seen in patients with vs. without tumours that expressed PD-L2 (23% vs. 10%; one-sided *P* = 0.022) (Supplemental Table 3). Additionally, higher ORR was noted in PD-L1-expressing tumours that also expressed PD-L2 (*n* = 108), compared with those that did not express PD-L2 (*n* = 39) (23% vs. 10%).

## Discussion

The last agent to receive approval from the US Food and Drug Administration as second-line therapy for HNSCC before 2016 was cetuximab in 2006, with a reported ORR of 13% (95% CI, 7–21%).^21^ In the subsequent decade, there has been little further advancement with second-line options plagued by low response rates (6–13%) and toxicity.^22,23^ Results presented herein after long-term follow-up confirm the anti-tumour activity and tolerability of pembrolizumab in the heavily pre-treated HNSCC patient population enrolled in KEYNOTE-012. This data set represents, to our knowledge, the longest follow-up period of patients with HNSCC who were treated with a PD-1 inhibitor and highlights long durability of responses achieved with some patients. Furthermore, the impact on survival seems to be significant; with a 38% 12-month OS rate, it is likely that a larger fraction of patients than just those who experienced the response will benefit from the treatment. Although the last patient with HNSCC was enrolled more than 18 months before these analyses were performed, the median DOR was not reached, and 65% of responses were ongoing, with some lasting for more than 30 months. Consistent with the reports from the individual cohorts, 18% of patients experienced CR or PR, and responses were observed, regardless of the HPV status.^5,6^ Despite the long-term treatment, pembrolizumab was well tolerated. The safety profile of pembrolizumab was consistent with profiles reported in other tumour types^16,24–27^ and no new safety risks were identified.

Although this trial did not mandate specific prior therapies, patients were heavily pre-treated. Importantly, 17% of patients treated previously with platinum and 15% treated previously with platinum and cetuximab treatment responded to pembrolizumab. This aligns with the 16% ORR in patients with HNSCC that progressed after platinum and cetuximab who were treated with pembrolizumab in the phase II KEYNOTE-055 trial.^7^ Another PD-1 inhibitor, nivolumab, was studied in CheckMate-141, in which patients were randomly assigned to receive nivolumab 3 mg/kg every 2 weeks, compared with the standard of care. The investigators reported an ORR of 13% and OS of 7.5 months. In a subset analysis, the HR was reported to be 0.55 among patients with PD-L1 expression of ≥1%.^4^ This enrichment for response based on PD-L1 status is consistent with the current study, although different assays and expression analyses were used.

As reported with individual cohorts, significant association between PD-L1 expression and response was observed when the analysis included the expression in both tumour and immune cells (CPS).^5,6^ Adding to these findings, we noted similar correlations of PD-L1 expression with OS and PFS using CPS. Association of PD-L1 expression per CPS and response to pembrolizumab was also reported in patients with advanced HNSCC in KEYNOTE-055.^7^ In addition, we demonstrated a significant association between PD-L1 and PD-L2 expression and found that PD-L2-expressing tumours were more likely to respond to pembrolizumab than non-PD-L2-expressing tumours. Nonetheless, patients without expression of either biomarker also responded to pembrolizumab at a clinically meaningful rate (9%). Therefore, although the use of PD-L1 and PD-L2 expression as biomarkers may enrich the response, patients whose tumours do not express these biomarkers may still respond to pembrolizumab.

These findings raise the questions of whether and how to use the biomarkers for patient selection. Currently, pembrolizumab is approved for use in recurrent or metastatic HNSCC after platinum therapy, regardless of the PD-L1 expression. Studies have already suggested the potential usefulness of an immune-related gene signature to predict the response to the PD-1 checkpoint blockade^28^; however, additional studies are necessary to determine whether a multigene profile or other biomarker can aid in the treatment decisions.

In conclusion, pembrolizumab exhibited durable anti-tumour activity, high survival rates, and acceptable safety in patients with heavily pre-treated advanced HNSCC. These data demonstrate that a subset of patients will receive long-term benefit from treatment with pembrolizumab, a paradigm rarely observed with existing cytotoxic or targeted therapies for recurrent or metastatic HNSCC. Combination studies of pembrolizumab with additional immune-targeting agents and with radiation therapy are under way.

## Electronic supplementary material

Supplemental Table 1

Supplemental Table 2

Supplemental Table 3

Supplemental Figure legend

Supplemental Figure 1a

Supplemental Figure 1b

Supplementary Protocol

## Availability of data and material

Merck & Co., Inc.’s data sharing policy including restrictions is available at http://engagezone.merck.com/ds_documentation.php. Requests for access to the clinical study data can be submitted through the EngageZone site or via email to dataaccess@merck.com.
