# Supplementary material for: Efficacy and safety of pembrolizumab in recurrent/metastatic head and neck squamous cell carcinoma: pooled analyses after long-term follow-up in KEYNOTE-012
Source: Br J Cancer. 2018 Jun 29;119(2):153–9. doi: 10.1038/s41416-018-0131-9 (PMC6048158; doi:10.1038/s41416-018-0131-9)
Supplement: Supplementary file 1 — Supplemental Table 1 [file 41416_2018_131_MOESM1_ESM.docx]

**Supplemental Table 1.** Tumour response to pembrolizumab per RECIST v1.1 by central imaging vendor review based on prior therapies

|  | **Progressed after platinum therapy**  ***n* = 174** | | **Progressed after platinum and cetuximab therapy**  ***n* = 110** | |
| --- | --- | --- | --- | --- |
|  | **No.** | **% (95% CI)** | **No.** | **% (95% CI)** |
| Overall response rate | 29 | 17 (12–23) | 16 | 15 (9–23) |
| Complete response | 8 | 5 (2–9) | 5 | 5 (2–10) |
| Partial response | 21 | 12 (8–18) | 11 | 10 (5–17) |
| Stable disease | 31 | 18 (12–24) | 18 | 16 (10–25) |
| Progressive disease | 86 | 49 (42–57) | 57 | 52 (42–61) |
| Non-CR/Non-PD | 6 | 3 (1–7) | 4 | 4 (1–9) |
| No assessment | 19 | 11 (7–17) | 13 | 12 (6–19) |
| Not evaluable | 3 | 2 (0.4–5) | 2 | 2 (0.2–6) |

Abbreviations: CR = complete response; PD = progressive disease; RECIST = Response Evaluation Criteria in Solid Tumors.

Only confirmed responses were included.

No assessment: patient had no postbaseline imaging.

Not evaluable: patient had postbaseline imaging, but images were not of sufficient quality to determine response.
