# Supplementary material for: Efficacy and safety of pembrolizumab in recurrent/metastatic head and neck squamous cell carcinoma: pooled analyses after long-term follow-up in KEYNOTE-012
Source: Br J Cancer. 2018 Jun 29;119(2):153–9. doi: 10.1038/s41416-018-0131-9 (PMC6048158; doi:10.1038/s41416-018-0131-9)
Supplement: Supplementary file 2 — Supplemental Table 2 [file 41416_2018_131_MOESM2_ESM.docx]

**Supplemental Table 2.** Reasons for censoring of responders from duration of response analysis (responders, *n* = 34)

| **Reason for censoring** | **No. (%)** |
| --- | --- |
| Censored patients | 24 (71) |
| Progressed or died after ≥2 missed visits | 0 (0) |
| New anti-cancer treatment started | 2 (6) |
| Ongoing response | 22 (65) |
| Patient in response and last disease assessment ≥5 months from data cut-off date | 4 (12) |
| Patient in response and last disease assessment <5 months from data cut-off date | 18 (53) |
