# Supplementary material for: Efficacy and safety of pembrolizumab in recurrent/metastatic head and neck squamous cell carcinoma: pooled analyses after long-term follow-up in KEYNOTE-012
Source: Br J Cancer. 2018 Jun 29;119(2):153–9. doi: 10.1038/s41416-018-0131-9 (PMC6048158; doi:10.1038/s41416-018-0131-9)
Supplement: Supplementary file 3 — Supplemental Table 3 [file 41416_2018_131_MOESM3_ESM.docx]

**Supplemental Table 3.** Tumour response to pembrolizumab per RECIST v1.1 by central imaging vendor review based on PD-L1 and PD-L2 expression status

| **Scoring method** | **Expression status** | **Responders/total in subpopulation,**  ***n/N*** | **ORR**  **% (95% CI)** | ***P* value** |
| --- | --- | --- | --- | --- |
| TPS | PD-L1^+^ | 22/123 | 18 (12–26) | .461 |
|  | PD-L1^−^ | 12/65 | 19 (10–30) |  |
| CPS | PD-L1^+^ | 32/152 | 21 (15–28) | .023 |
|  | PD-L1^−^ | 2/36 | 6 (1–19) |  |
| CPS | PD-L2^+^ | 25/111 | 23 (15–31) | .022 |
|  | PD-L2^−^ | 6/61 | 10 (4–20) |  |

Abbreviations: CPS = combined positive score; ORR = overall response rate; RECIST = Response Evaluation Criteria in Solid Tumors; TPS = tumour proportion score.

Only confirmed responses are included.
