# Supplementary material for: Efficacy and safety of pembrolizumab in recurrent/metastatic head and neck squamous cell carcinoma: pooled analyses after long-term follow-up in KEYNOTE-012
Source: Br J Cancer. 2018 Jun 29;119(2):153–9. doi: 10.1038/s41416-018-0131-9 (PMC6048158; doi:10.1038/s41416-018-0131-9)
Supplement: Supplementary file 4 — Supplemental Figure legend [file 41416_2018_131_MOESM4_ESM.docx]

**Supplemental Material**

**Supplemental Figure 1.** Kaplan-Meier estimates of (**A**) progression-free survival per RECIST v1.1 by central imaging vendor review and (**B**) overall survival based on PD-L1 expression determined using tumour proportion score (TPS) or combined positive score (CPS). Abbreviations: RECIST, Response Evaluation Criteria in Solid Tumors.
