# Supplementary material for: Efficacy and safety of pembrolizumab in recurrent/metastatic head and neck squamous cell carcinoma: pooled analyses after long-term follow-up in KEYNOTE-012
Source: Br J Cancer. 2018 Jun 29;119(2):153–9. doi: 10.1038/s41416-018-0131-9 (PMC6048158; doi:10.1038/s41416-018-0131-9)
Supplement: Supplementary file 7 — Supplementary Protocol [file 41416_2018_131_MOESM7_ESM.pdf]

Merck & Co., Inc. policy on posting of redacted study protocols on journal websites is described in Guidelines for Publication of Clinical Trials in the Scientific Literature on the www.merck.com website.

For publicly posted protocols, the Company redacts content that contains proprietary and private information.

This report may include approved and non-approved uses, formulations, or treatment regimens. The results reported may not reflect the overall profile of a product. Before prescribing any product mentioned in this report, healthcare professionals should consult local prescribing information for the product approved in their country.

Copyright © 2018 Merck Sharp & Dohme Corp., a subsidiary of Merck & Co., Inc.

All Rights Reserved. Not for regulatory or commercial use.

THIS PROTOCOL AMENDMENT AND ALL OF THE INFORMATION RELATING TO IT ARE CONFIDENTIAL AND PROPRIETARY PROPERTY OF MERCK SHARP & DOHME CORP., A SUBSIDIARY OF MERCK & CO., INC., WHITEHOUSE STATION, NJ, U.S.A.

SPONSOR:

Merck Sharp & Dohme Corp., a subsidiary of Merck & Co., Inc.
(hereafter referred to as the Sponsor or Merck)
One Merck Drive
P.O. Box 100
Whitehouse Station, NJ 08889-0100, U.S.A.

Protocol-specific Sponsor Contact information can be found in the Investigator Trial File Binder.

TITLE:

A Phase Ib Multi-Cohort Study of MK-3475 in Subjects with Advanced Solid Tumors

IND NUMBER: 110,080

EudraCT NUMBER: 2012-005771-14

TABLE OF CONTENTS

SUMMARY OF CHANGES 10
1.0 TRIAL SUMMARY 11
2.0 TRIAL DESIGN 12
2.1 Trial Design 12
2.2 Trial Diagram 14
3.0 OBJECTIVE(S) & HYPOTHESIS(ES) 14
3.1 Primary Objective(s) & Hypothesis(es) 14
3.2 Secondary Objective(s) & Hypothesis(es) 15
3.3 Exploratory Objective 17
4.0 BACKGROUND & RATIONALE 17
4.1 Background 17
4.1.1 Pharmaceutical and Therapeutic Background 17
4.1.2 Pre-clinical and Clinical Trials 18
4.1.3 Ongoing Clinical Trials 18
4.2 Rationale 18
4.2.1 Rationale for the Trial and Selected Subject Population 18
4.2.2 Rationale for Dose Selection/Regimen/Modification 19
4.2.3 Rationale for Endpoints 20
4.2.3.1 Efficacy Endpoints 20
4.2.3.2 Safety Endpoints 21
4.2.3.3 Biomarker Research 22
4.2.3.4 Future Biomedical Research 23
5.0 METHODOLOGY 23
5.1 Entry Criteria 23
5.1.1 Diagnosis/Condition for Entry into the Trial 23
5.1.2 Subject Inclusion Criteria 23
5.1.3 Subject Exclusion Criteria 25
5.2 Trial Treatments 27
5.2.1 Dose Selection/Modification 28

5.2.1.1 Dose Selection 28
5.2.1.2 Dose Modification 28
5.2.2 Timing of Dose Administration 30
5.2.3 Trial Blinding/Masking 32
5.3 Randomization or Treatment Allocation 32
5.4 Stratification 32
5.5 Concomitant Medications/Vaccinations (allowed & prohibited) 32
5.5.1 Acceptable Concomitant Medications 32
5.5.2 Prohibited Concomitant Medications 33
5.6 Rescue Medications & Supportive Care 34
5.6.1 Supportive Care Guidelines 34
5.7 Diet/Activity/Other Considerations 37
5.7.1 Diet 37
5.7.2 Contraception 38
5.7.3 Use in Pregnancy 38
5.7.4 Use in Nursing Women 38
5.8 Subject Withdrawal/Discontinuation Criteria 39
5.8.1 Discontinuation of Study Therapy after CR 40
5.9 Subject Replacement Strategy 40
5.10 Beginning and End of the Trial 41
5.11 Clinical Criteria for Early Trial Termination 41
6.0 TRIAL FLOW CHART 42
6.1 Study Flow Chart for Cohorts A, B, C and D (10 mg/kg Q2W dosing) 42
6.2 Study Flow Chart for Cohort B2 (200 mg Q3W dosing) 46
6.3 Second Course Phase (Retreatment ONLY) for Cohorts A, B, C and D 49
6.4 Second Course Phase (Retreatment ONLY) for Cohort B2 51
7.0 TRIAL PROCEDURES 53
7.1 Trial Procedures 53
7.1.1 Administrative Procedures 53
7.1.1.1 Informed Consent 53
7.1.1.1.1 General Informed Consent 53

7.1.1.1.2 Consent and Collection of Specimens for Future Biomedical Research 54
7.1.1.2 Inclusion/Exclusion Criteria 54
7.1.1.3 Subject Identification Card 54
7.1.1.4 Medical History 54
7.1.1.5 Prior and Concomitant Medications Review 54
7.1.1.5.1 Prior Medications 54
7.1.1.5.2 Concomitant Medications 54
7.1.1.6 Disease Details and Treatments 55
7.1.1.6.1 Disease Details 55
7.1.1.6.2 Prior Treatment Details 55
7.1.1.6.3 Subsequent Anti-neoplastic Therapy Status 55
7.1.1.7 Assignment of Screening Number 55
7.1.1.8 Assignment of Randomization Number 55
7.1.1.9 Trial Compliance (Medication/Diet/Activity/Other) 55
7.1.2 Clinical Procedures/Assessments 56
7.1.2.1 Adverse Event (AE) Monitoring 56
7.1.2.2 Physical Exam 56
7.1.2.2.1 Full Physical Exam 56
7.1.2.2.2 Directed Physical Exam 56
7.1.2.3 Vital Signs 57
7.1.2.4 Eastern Cooperative Oncology Group (ECOG) Performance Scale 57
7.1.2.5 Tumor Imaging and Assessment of Disease 57
7.1.2.5.1 Assessment of Disease 57
7.1.2.5.2 Initial Tumor Imaging 59
7.1.2.5.3 Tumor Imaging During Trial 59
7.1.2.6 Tumor Tissue Collection and Correlative Studies Blood Sampling 59
7.1.3 Laboratory Procedures/Assessments 61
7.1.3.1 Laboratory Safety Evaluations (Hematology, Chemistry and Urinalysis) .. 62
7.1.3.2 Pharmacokinetic/Pharmacodynamic Evaluations 63
7.1.3.2.1 Blood Collection for Serum MK-3475 63
7.1.3.2.2 Blood Collection for Anti-MK-3475 Antibodies 63

7.1.3.3 Future Biomedical Research 63
7.1.4 Other Procedures 63
7.1.4.1 Withdrawal/Discontinuation 63
7.1.4.1.1 Withdrawal From Future Biomedical Research 64
7.1.4.2 Blinding/Unblinding 64
7.1.5 Visit Requirements 64
7.1.5.1 Screening 64
7.1.5.1.1 Pre-screening Period 64
7.1.5.1.2 Screening Period 65
7.1.5.2 Treatment Period 65
7.1.5.2.1 Second Course Phase (Retreatment Period) 65
7.1.5.3 Post-Treatment Visits 67
7.1.5.3.1 Safety Follow-Up Visit 67
7.1.5.4 Follow-up Visits 67
7.1.5.4.1 Survival Follow-up 67
7.2 Assessing and Recording Adverse Events 68
7.2.1 Definition of an Overdose for This Protocol and Reporting of Overdose to the Sponsor 69
7.2.2 Reporting of Pregnancy and Lactation to the Sponsor 69
7.2.3 Immediate Reporting of Adverse Events to the Sponsor 70
7.2.3.1 Serious Adverse Events 70
7.2.3.2 Events of Clinical Interest 71
7.2.3.3 Protocol-Specific Exceptions to Serious Event Reporting 72
7.2.4 Evaluating Adverse Events 72
7.2.5 Sponsor Responsibility for Reporting Adverse Events 75
8.0 STATISTICAL ANALYSIS PLAN 75
8.1 Statistical Analysis Plan Summary 75
8.1.1 Efficacy Analyses 75
8.1.2 Safety Analyses 77
8.1.3 Power and Sample Size 77
8.1.4 Interim Analysis 78
8.2 Statistical Analysis Plan 80

8.2.1 Responsibility for Analyses/In-House Blinding ... 80
8.2.2 Hypotheses/Estimation ... 80
8.2.3 Analysis Endpoints ... 80
8.2.3.1 Efficacy/Immunogenicity/Pharmacokinetics Endpoints ... 80
8.2.3.2 Safety Endpoints ... 81
8.2.4 Analysis Populations ... 81
8.2.4.1 Efficacy Analysis Populations ... 81
8.2.4.2 Safety Analysis Populations ... 81
8.2.5 Statistical Methods ... 82
8.2.5.1 Statistical Methods for Efficacy Analyses ... 82
8.2.5.2 Statistical Methods for Safety Analyses ... 83
8.2.5.3 Summaries of Baseline Characteristics, Demographics, and Other Analyses ... 84
8.2.5.3.1 Demographic and Baseline Characteristics ... 84
8.2.6 Multiplicity ... 84
8.2.7 Sample Size and Power Calculations ... 84
8.2.8 Subgroup Analyses and Effect of Baseline Factors ... 86
8.2.9 Interim Analyses ... 86
8.2.9.1 Efficacy Interim Analyses ... 86
8.2.9.2 Safety Interim Analyses ... 87
8.2.10 Compliance (Medication Adherence) ... 88
8.2.11 Extent of Exposure ... 88
9.0 LABELING, PACKAGING, STORAGE AND RETURN OF CLINICAL SUPPLIES ... 89
9.1 Investigational Product ... 89
9.2 Packaging and Labeling Information ... 89
9.3 Clinical Supplies Disclosure ... 89
9.4 Storage and Handling Requirements ... 89
9.5 Returns and Reconciliation ... 89
9.6 Standard Policies ... 90
10.0 ADMINISTRATIVE AND REGULATORY DETAILS ... 90
10.1 Confidentiality ... 90

10.1.1 Confidentiality of Data ... 90
10.1.2 Confidentiality of Subject Records ... 90
10.1.3 Confidentiality of Investigator Information ... 91
10.1.4 Confidentiality of IRB/IEC Information ... 91
10.2 Compliance with Financial Disclosure Requirements ... 91
10.3 Compliance with Law, Audit and Debarment ... 92
10.4 Compliance with Trial Registration and Results Posting Requirements ... 94
10.5 Quality Management System ... 94
10.6 Data Management ... 95
10.7 Publications ... 95
11.0 LIST OF REFERENCES ... 96
12.0 APPENDICES ... 101
12.1 Merck Code of Conduct for Clinical Trials ... 101
12.2 Collection and Management of Specimens for Future Biomedical Research ... 103
12.3 Understanding the Intent, Scope and Public Health Benefits of Exploratory Biomarker Research: A Guide for IRBs/IECs and Investigational Site Staff ... 110
12.4 ECOG Performance Status ... 121
12.5 Common Terminology Criteria for Adverse Events V4.0 (CTCAE) ... 122
12.6 Response Evaluation Criteria in Solid Tumors (RECIST) 1.1 Criteria For Evaluating Response in Solid Tumors ... 123
13.0 SIGNATURES ... 124
13.1 Sponsor's Representative ... 124
13.2 Investigator ... 124

LIST OF TABLES

Table 1 Adequate Organ Function Laboratory Values 25
Table 2 Trial Treatment 27
Table 3 Dose Modification Guidelines for Drug-Related Adverse Events. 29
Table 4 Imaging and Treatment After 1st Radiologic Evidence of PD 31
Table 5 Infusion Reaction Treatment Guidelines 37
Table 6 Imaging and Treatment after 1st radiologic evidence of PD 58
Table 7 Laboratory Tests 62
Table 8 Evaluating Adverse Events 73
Table 9 Primary Analysis Strategy for Efficacy Endpoints 76
Table 10 Summary of Interim Analysis Strategy 79
Table 11 Analysis Strategy for Key Efficacy Variables 83
Table 12 Operating Characteristics of Interim Analysis Rule for Triple Negative Breast Cancer – Pause Enrollment if ≤ 2/10 Subjects Respond 86
Table 13 Operating Characteristics of Interim Analysis Rule for Head/Neck Cancer (HPV negative) and Urothelial Cancer– Pause Enrollment if ≤ 1/10 subjects respond 87
Table 14 Estimate of Incidence of Grade 4/5 events and 95% Upper Confidence Bound Based on Hypothetical Number of Subjects with Event Out of 10 Subjects Evaluated 88
Table 15 Product Descriptions 89

LIST OF FIGURES

Figure 1 Trial Design 14

SUMMARY OF CHANGES

PRIMARY REASON(S) FOR THIS AMENDMENT:

Section Number (s)	Section Title(s)	Description of Change (s)	Rationale
8.1.1<br>8.2.4.1<br>8.2.5.1	Efficacy Analyses<br>Efficacy Analysis Populations<br>Statistical Methods for Efficacy Analyses	The Intent to Treat (ITT) population was changed to the All Subjects as Treated (ASaT) population.	To align with modifications to planned supportive efficacy analyses.

ADDITIONAL CHANGE(S) FOR THIS AMENDMENT:

Section Number (s)	Section Title (s)	Description of Change (s)	Rationale
8.2.1	Responsibility for Analyses / In-House Blinding	Clarified that access to the PD-L1 subject-level biomarker results for subjects in Cohort B2 for the purpose of summary reporting of PD-L1 level will be limited until the time that a clinical study report (CSR) is generated.	Knowledge of PD-L1 data for subjects in Cohort B2 is required for analysis of Secondary Objective (6).

1.0 TRIAL SUMMARY

Abbreviated Title	Phase Ib Multicohort Study of MK-3475 in Subjects with Advanced Solid Tumors
Trial Phase	Ib
Clinical Indication	The treatment of subjects with triple negative breast cancer, head/neck cancer, urothelial tract cancer, or gastric cancer.
Trial Type	Interventional
Type of control	No treatment control
Route of administration	Intravenous
Trial Blinding	Unblinded Open-label
Treatment Groups	MK-3475 10 mg/kg every 2 weeks (Cohort A, B, C and D)<br>MK-3475 200 mg every 3 weeks (Cohort B2)
Number of trial subjects	Approximately 114 subjects originally planned for enrollment into Cohorts A, B, C, and D, plus 110 additional H/N expansion (Cohort B2) subjects will be enrolled.
Estimated duration of trial	The sponsor estimates that the trial will require approximately 42 months from the time the first subject signs the informed consent until the last subject's last visit.
Duration of Participation	Each subject will participate in the trial from the time the subject signs the Informed Consent Form (ICF) through the final protocol-specified contact. After a screening phase of 28 days, eligible subjects will receive treatment on Day 1 of each 2-week dosing cycle (Cohorts A, B, C, and D) or 3-week dosing cycle (Cohort B2). Treatment with MK-3475 will continue until documented confirmed disease progression, unacceptable adverse event(s), intercurrent illness that prevents further administration of treatment, investigator's decision to withdraw the subject, subject withdraws consent, pregnancy of the subject, noncompliance with trial treatment or procedure requirements, subject receives 24 months of study medication, or administrative reasons. Subjects who attain a complete response may consider stopping trial treatment if they meet criteria for holding therapy. Subjects who stop trial treatment after receiving 24 months of study medication for reasons other than disease progression or intolerability or who attain a complete response and stop trial treatment may be eligible for up to one year of retreatment after experiencing disease progression. The decision to retreat will be at the discretion of the investigator only if they meet the criteria for retreatment and the trial is ongoing. After the end of treatment, each subject will be followed for 30 days for adverse event monitoring (serious adverse events will be collected for 90 days after the end of treatment or 30 days after the end of treatment if the subject initiates new anticancer therapy, whichever is earlier). Subjects who discontinue for reasons other than disease progression will have post-treatment follow-up for disease status until disease progression, initiating a non-study cancer treatment, withdrawing consent, or becoming lost to follow-up. All subjects will be followed by telephone for overall survival until death, withdrawal of consent, or the end of the study.

2.0 TRIAL DESIGN

2.1 Trial Design

This is a multicenter, nonrandomized, multi-cohort trial of MK-3475 in subjects with advanced solid tumors. Subjects will be enrolled into Cohort A for triple negative breast cancer (TNBC), Cohort B for the initial head and neck (H/N) cancer cohort and Cohort B2 for the H/N cancer expansion cohort, Cohort C for urothelial tract cancer, or Cohort D for gastric cancer. Only subjects with PD-L1 positive tumors will be enrolled in Cohorts A, B, C and D. Subjects in Cohort B2 may be enrolled regardless of PD-L1 status. Approximately 114 subjects were planned to be enrolled in Cohorts A, B, C and D of this trial to examine the safety and efficacy of MK-3475 in these populations. Approximately 110 subjects will be enrolled into Cohort B2 of the study to further explore the safety and efficacy in the head and neck cancer population at a different dose and schedule of MK-3475 and including both PD-L1 positive and negative subjects. Subjects enrolled in Cohorts A, B, C and D will receive 10 mg/kg of MK-3475 administered every 2 weeks. Subjects enrolled in Cohort B2 will receive 200 mg of MK-3475 administered every 3 weeks. Subjects will be evaluated every 8 weeks (56 days ± 7 days) with radiographic imaging to assess response to treatment. RECIST 1.1 response rate as assessed by independent central radiology review will be used as the primary efficacy endpoint for Cohorts A, B, C and D. RECIST 1.1 response rate as assessed by independent central radiology review will be used as the primary efficacy endpoint for Cohort B2. RECIST 1.1 will be adapted as described in Section 4.2.3.1 to accommodate for the tumor response patterns seen with MK-3475 treatment (e.g., tumor flare), and this adapted RECIST will be used by the sites for treatment decisions in all cohorts. Adverse events will be monitored throughout the trial and graded in severity according to the guidelines outlined in the NCI Common Terminology Criteria for Adverse Events (CTCAE) version 4.0. Treatment with MK-3475 will continue until documented disease progression, unacceptable adverse event(s), intercurrent illness that prevents further administration of treatment, investigator's decision to withdraw the subject, subject withdraws consent, pregnancy of the subject, noncompliance with trial treatment or procedure requirements, completion of 24 months of treatment with MK-3475, or administrative reasons. Subjects who attain an investigator-determined confirmed complete response (CR) may consider stopping trial treatment after receiving at least 24 weeks of treatment. Subjects who discontinue after at least 24 months of therapy for reasons other than disease progression or intolerability or who discontinue after attaining a CR may be eligible for up to one year of retreatment after they have experienced radiographic disease progression. The decision to retreat will be at the discretion of the investigator only if no cancer treatment was administered since the last dose of MK-3475, the subject still meets the safety parameters listed in the Inclusion/Exclusion criteria and the trial remains open (refer to Section 7.1.5.2.1 for further details). After the end of treatment, each subject will be followed for 30 days for adverse event monitoring (serious adverse events will be collected for 90 days after the end of treatment or 30 days after the end of treatment if the subject initiates new anticancer therapy, whichever is earlier). Subjects who discontinue treatment for reasons other than disease progression will have post-treatment follow-up for disease status until disease progression, initiating a non-study cancer treatment, withdrawing consent, or becoming lost to follow-up. All subjects will be followed by telephone contact for overall survival until death, withdrawal of consent or the end of the study, whichever comes first.

The primary objectives of the trial are to determine the safety, tolerability, and anti-tumor activity of MK-3475 in subjects with advanced solid tumors receiving either the 10 mg/kg Q2W dose (Cohorts A, B, C and D) or the 200 mg Q3W dose (Cohort B2). Secondary objectives include progression-free survival (PFS), overall survival (OS) and response duration in subjects with advanced solid tumors. In addition, the anti-tumor activity of MK-3475 in subjects with PD-L1 positive advanced human papillomavirus (HPV) positive head/neck cancer will be evaluated as a secondary objective. The relationship between candidate efficacy/resistance biomarkers (including PD-L1 expression in the tumor and its microenvironment) and anti-tumor activity of MK-3475 will also be investigated as a secondary objective. The pharmacokinetic (PK) properties of MK-3475 in the advanced solid tumor population will be investigated as an exploratory objective.

Participation in this trial will be dependent upon supplying tissue from an archival tissue sample or newly obtained biopsy of a tumor lesion not previously irradiated (subjects in the H/N cohort may submit tissue from a previously-irradiated lesion). This specimen will be evaluated at a central laboratory for expression status of PD-L1 by immunohistochemistry (IHC). Only subjects with PD-L1 positive tumors will be enrolled into Cohorts A, B, C and D of the trial. Both PD-L1 positive and negative subjects will be enrolled into Cohort B2, and the clinical activity in both subsets will be evaluated.

This study will be conducted in conformance with Good Clinical Practices.

Specific procedures to be performed during the trial, as well as their prescribed times and associated visit windows, are outlined in the Trial Flow Chart - Section 6.0. Details of each procedure are provided in Section 7.0 – Trial Procedures.

2.2 Trial Diagram

The trial design is depicted in Figure 1 below.

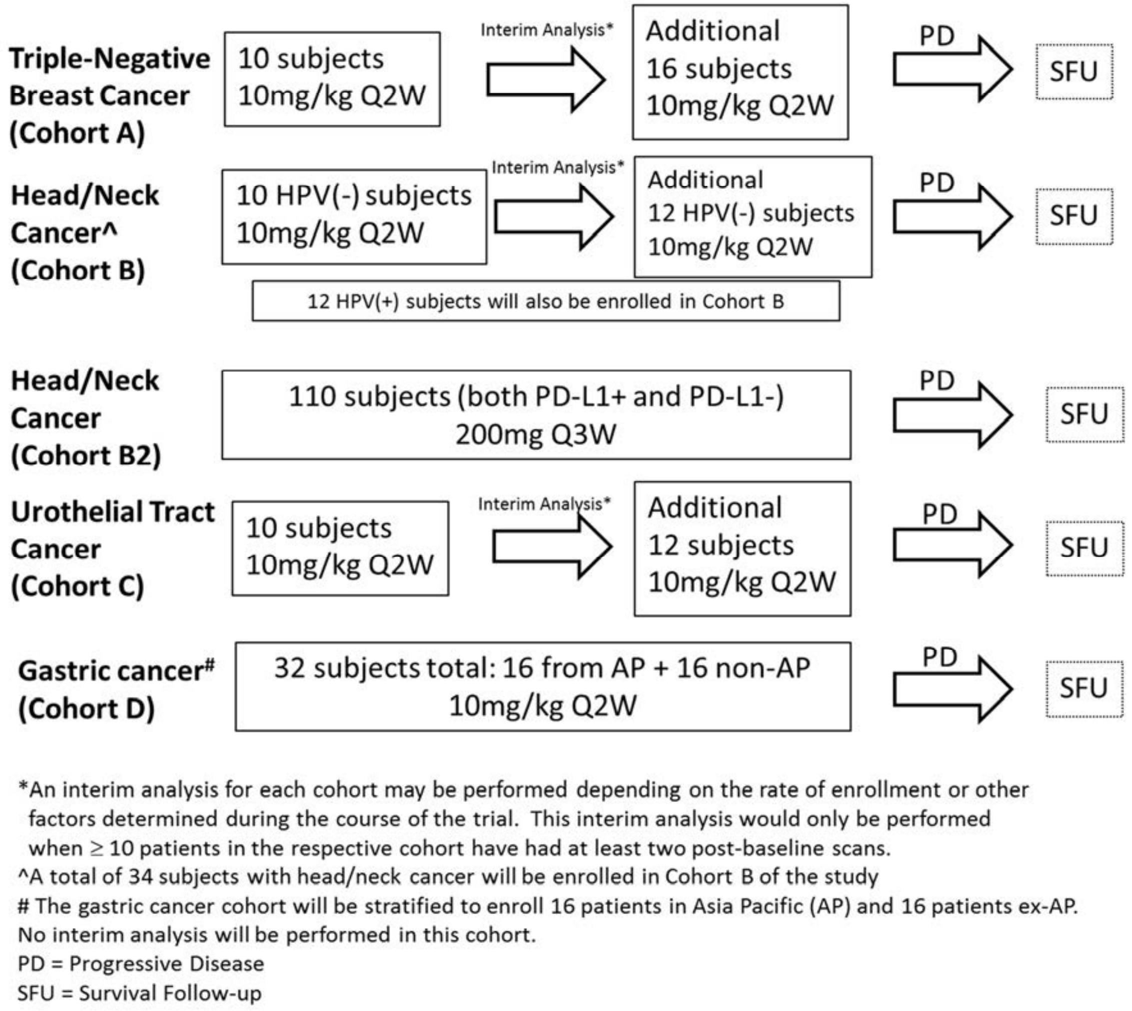

Figure 1 Trial Design

3.0 OBJECTIVE(S) & HYPOTHESIS(ES)

3.1 Primary Objective(s) & Hypothesis(es)

(1) **Objective:** To determine the safety and tolerability of the 10 mg/kg Q2W dose of MK-3475 in subjects with PD-L1 positive advanced solid tumors enrolled into Cohorts A, B, C, and D.

Hypothesis: Intravenous administration of the single agent 10 mg/kg Q2W dose of MK-3475 is sufficiently well-tolerated to permit continued clinical investigation.

(2) **Objective:** To evaluate anti-tumor activity of the 10 mg/kg Q2W dose of MK-3475 in subjects with PD-L1 positive advanced solid tumors enrolled into Cohorts A, B, C, and D based on RECIST 1.1 assessed by independent central radiology review.

a. **Hypothesis:** Intravenous administration of single agent MK-3475 10 mg/kg Q2W to subjects with PD-L1 positive triple negative advanced breast cancer (Cohort A) will result in a clinically meaningful overall response rate (ORR) based on RECIST 1.1.

b. **Hypothesis:** Intravenous administration of single agent MK-3475 10 mg/kg Q2W to subjects with PD-L1 positive HPV negative advanced head and neck cancer (Cohort B) will result in a clinically meaningful overall response rate (ORR) based on RECIST 1.1.

c. **Hypothesis:** Intravenous administration of single agent MK-3475 10 mg/kg Q2W to subjects with PD-L1 positive advanced urothelial tract cancer (Cohort C) will result in a clinically meaningful overall response rate (ORR) based on RECIST 1.1.

d. **Hypothesis:** Intravenous administration of single agent MK-3475 10 mg/kg Q2W to subjects with PD-L1 positive advanced gastric cancer (Cohort D) will result in a clinically meaningful overall response rate (ORR) based on RECIST 1.1.

(3) **Objective:** To determine the safety and tolerability of the 200 mg Q3W dose of MK-3475 in subjects with advanced head and neck cancer enrolled into Cohort B2.

Hypothesis: Intravenous administration of single agent MK-3475 200 mg Q3W in subjects with advanced head and neck cancer is sufficiently well-tolerated to permit continued clinical investigation.

(4) **Objective:** To evaluate anti-tumor activity of MK-3475 200 mg Q3W (Cohort B2) in subjects with advanced head and neck cancer enrolled into Cohort B2 based on RECIST 1.1 assessed by independent central radiology review .

Hypothesis: Intravenous administration of the single agent 200 mg Q3W dose of MK-3475 to subjects with advanced head and neck cancer (Cohort B2) will result in a clinically meaningful overall response rate (ORR) based on RECIST 1.1 assessed by independent central radiology review**.**

3.2 Secondary Objective(s) & Hypothesis(es)

(1) **Objective**: To evaluate the anti-tumor activity of MK-3475 in subjects with PD-L1 positive advanced HPV positive head/neck cancer in Cohort B as determined by independent central radiology review.

Hypothesis: Intravenous administration of single agent MK-3475 to subjects with PD-L1 positive HPV positive advanced head and neck cancer enrolled into Cohort B will result in a clinically meaningful overall response rate (ORR) based on RECIST 1.1 as assessed by an independent central radiology review.

(2) **Objective:** To evaluate the anti-tumor activity of MK-3475 in subjects with PD-L1 positive advanced gastric cancer in the Asia Pacific region as assessed by independent central radiology review.

Hypothesis: Intravenous administration of single agent MK-3475 to subjects with PD-L1 positive advanced gastric cancer in the Asia Pacific region will result in a clinically meaningful overall response rate (ORR) based on RECIST 1.1 as assessed by independent central radiology review.

(3) **Objective:** To evaluate the anti-tumor activity of MK-3475 in subjects with advanced head and neck cancer previously treated with cetuximab and platinum enrolled in Cohort B or Cohort B2 based on RECIST 1.1 as assessed by independent central radiology review.

Hypothesis: Intravenous administration of single agent MK-3475 in subjects with advanced head and neck cancer previously treated with cetuximab and platinum enrolled in Cohort B or Cohort B2 will result in a clinically meaningful overall response rate (ORR) based on RECIST 1.1 as assessed by independent central radiology review.

(4) **Objective:** To evaluate the anti-tumor activity of MK-3475 in subjects with PD-L1 positive advanced solid tumors based on RECIST 1.1 as determined by the Investigator (Cohorts A, B, C and D)

(5) **Objective:** To evaluate the anti-tumor activity of MK-3475 at 200 mg Q3W in subjects with advanced head and neck cancer (Cohort B2) based on RECIST 1.1 as assessed by the Investigator.

(6) **Objective:** To investigate the correlation between PD-L1 expression and anti-tumor activity of pembrolizumab in subjects with advanced head and neck cancer enrolled into Cohort B2.

(7) Objective: To investigate the relationship between candidate efficacy/resistance biomarkers and anti-tumor activity of pembrolizumab utilizing pre- and post-treatment tumor biopsies and sampling.

(8) **Objective**: To evaluate the progression-free survival (PFS) in subjects with advanced solid tumors receiving MK-3475.

(9) **Objective**: To evaluate the overall survival (OS) in subjects with advanced solid tumors receiving MK-3475.

(10) **Objective**: To evaluate the response duration in subjects with advanced solid tumors receiving MK-3475.

3.3 Exploratory Objective

(1) **Objective:** To explore the PK profile of MK-3475 in the advanced solid tumor population.

4.0 BACKGROUND & RATIONALE

Redacted

Redacted

Redacted

Redacted

Redacted

Redacted

Redacted

5.0 METHODOLOGY

5.1 Entry Criteria

5.1.1 Diagnosis/Condition for Entry into the Trial

Male/Female subjects with triple negative breast cancer (Cohort A), head/neck cancer (Cohort B and Cohort B2), urothelial tract cancer (Cohort C), or gastric cancer (Cohort D) of at least 18 years of age will be enrolled in this trial.

5.1.2 Subject Inclusion Criteria

In order to be eligible for participation in this trial, the subject must:

1. Be willing and able to provide written informed consent/assent for the trial. The subject may also provide consent/assent for Future Biomedical Research. However, the subject may participate in the main trial without participating in Future Biomedical Research.

2. Be ≥ 18 years of age on day of signing informed consent.

3. Have histologically or cytologically-confirmed diagnosis of cancer that is recurrent, metastatic, or persistent and meet the following corresponding requirements for the cohort of the study they will enroll into. There is no limit to the number of prior treatment regimens.

    a. Cohort A: Have diagnosis of triple negative breast cancer (Estrogen, Progesterone, and HER2 negative carcinoma of the breast)

    b. Cohort B and B2: Have a diagnosis of squamous cell carcinoma of the head and neck

        Note: A subset of subjects enrolled to Cohort B known to have HPV positive head and neck squamous cell cancer will be assessed.

        Note: Cohort B2 will enroll both HPV positive and HPV negative subjects

    c. Cohort C: Have a diagnosis of urothelial tract cancer of the renal pelvis, ureter, bladder, or urethra. Both transitional cell and non-transitional cell histologies are allowed.

    d. Cohort D: Have a diagnosis of adenocarcinoma of the stomach or gastro-esophageal junction

4. Have provided tissue for biomarker analysis from an archival tissue sample or newly obtained core or excisional biopsy of a tumor lesion not previously irradiated. (Subjects with H/N cancer may provide tissue from a previously irradiated lesion.)

5. Cohorts A, B, C and D only: Have a PD-L1 positive tumor as determined by IHC at a central laboratory from either an archived formalin fixed paraffin embedded (FFPE) tumor sample or a newly obtained biopsy.

6. Have measurable disease based on RECIST 1.1. Tumor lesions situated in a previously irradiated area are considered measurable if progression has been demonstrated in such lesions.

7. Have a performance status of 0 or 1 on the ECOG Performance Scale.

8. Demonstrate adequate organ function as defined in Table 1, all screening labs should be performed within 10 days of treatment initiation.

Table 1 Adequate Organ Function Laboratory Values

System	Laboratory Value
**Hematological**	
Absolute neutrophil count (ANC)	≥1,500 /mcL
Platelets	≥100,000 / mcL
Hemoglobin	≥9 g/dL or ≥5.6 mmol/L
**Renal**	
Creatinine **OR**<br>Measured or calculated[a] creatinine clearance<br>(GFR can also be used in place of creatinine or CrCl)	≤1.5 X upper limit of normal (ULN) **OR**<br>≥60 mL/min for subject with creatinine levels > 1.5 X institutional ULN
**Hepatic**	
Total bilirubin	≤ 1.5 X ULN **OR**
	Direct bilirubin ≤ ULN for subjects with total bilirubin levels > 1.5 ULN
AST (SGOT) and ALT (SGPT)	≤ 2.5 X ULN **OR**<br>≤ 5 X ULN for subjects with liver metastases
**Coagulation**	
International Normalized Ratio (INR) or Prothrombin Time (PT)<br>Activated Partial Thromboplastin Time (aPTT)	≤1.5 X ULN unless subject is receiving anticoagulant therapy as long as PT or PTT is within therapeutic range of intended use of anticoagulants<br>≤1.5 X ULN unless subject is receiving anticoagulant therapy as long as PT or PTT is within therapeutic range of intended use of anticoagulants
[a] Creatinine clearance should be calculated per institutional standard.	

9. Female subject of childbearing potential should have a negative urine or serum pregnancy test within 72 hours prior to receiving the first dose of study medication. If the urine test is positive or cannot be confirmed as negative, a serum pregnancy test will be required.

10. Female subjects of childbearing potential should be willing to use 2 methods of birth control or be surgically sterile, or abstain from heterosexual activity for the course of the study through 120 days after the last dose of study medication (Reference Section 5.7.2). Subjects of childbearing potential are those who have not been surgically sterilized or have not been free from menses for > 1 year.

11. Male subjects should agree to use an adequate method of contraception starting with the first dose of study therapy through 120 days after the last dose of study therapy.

5.1.3 Subject Exclusion Criteria

The subject must be excluded from participating in the trial if the subject:

1. Is currently participating in or has participated in a study of an investigational agent or using an investigational device within 4 weeks of the first dose of treatment.

2. Has a diagnosis of immunosuppression or is receiving systemic steroid therapy or any other form of immunosuppressive therapy within 7 days prior to the first dose of trial treatment.

3. Has had a prior anti-cancer monoclonal antibody within 4 weeks prior to study Day 1 or who has not recovered (i.e., ≤ Grade 1 or at baseline) from adverse events due to agents administered more than 4 weeks earlier.

4. Has had prior chemotherapy, targeted small molecule therapy, or radiation therapy within 2 weeks prior to study Day 1 or who has not recovered (i.e., ≤ Grade 1 or at baseline) from adverse events due to a previously administered agent.

    - Note: Subjects with ≤ Grade 2 neuropathy are an exception to this criterion and may qualify for the study.

    - Note: If subject received major surgery, they must have recovered adequately from the toxicity and/or complications from the intervention prior to starting therapy.

5. Has a known additional malignancy that is progressing or requires active treatment. Exceptions include basal cell carcinoma of the skin, squamous cell carcinoma of the skin, or in situ cervical cancer that has undergone potentially curative therapy.

6. Has known active central nervous system (CNS) metastases and/or carcinomatous meningitis. Subjects with previously treated brain metastases may participate provided they are stable (without evidence of progression by imaging for at least four weeks prior to the first dose of trial treatment and any neurologic symptoms have returned to baseline), have no evidence of new or enlarging brain metastases, and are not using steroids for at least 7 days prior to trial treatment.

7. Has an active automimmune disease requiring systemic treatment within the past 3 months or a documented history of clinically severe autoimmune disease, or a syndrome that requires systemic steroids or immunosuppressive agents. Subjects with vitiligo, diabetes mellitus type I, or resolved childhood asthma/atopy would be an exception to this rule. Subjects that require intermittent use of bronchodilators or local steroid injections would not be excluded from the study. Subjects with hypothyroidism stable on hormone replacement or Sjorgen's syndrome will not be excluded from the study.

8. Has evidence of interstitial lung disease.

9. Has an active infection requiring systemic therapy.

10. Has a history or current evidence of any condition, therapy, or laboratory abnormality that might confound the results of the trial, interfere with the subject's participation for the full duration of the trial, or is not in the best interest of the subject to participate, in the opinion of the treating investigator.

11. Has known psychiatric or substance abuse disorders that would interfere with cooperation with the requirements of the trial.

12. Is pregnant or breastfeeding, or expecting to conceive or father children within the projected duration of the trial, starting with the pre-screening or screening visit through 120 days after the last dose of trial treatment.

13. Has received prior therapy with an anti-PD-1, anti-PD-L1, anti-PD-L2, anti-CD137, or anti-Cytotoxic T-lymphocyte-associated antigen-4 (CTLA-4) antibody (including ipilimumab or any other antibody or drug specifically targeting T-cell co-stimulation or checkpoint pathways).

14. Has a known history of Human Immunodeficiency Virus (HIV) (HIV 1/2 antibodies).

15. Has known active Hepatitis B (e.g., HBsAg reactive) or Hepatitis C (e.g., HCV RNA [qualitative] is detected).

16. Has received a live vaccine within 30 days of planned start of study therapy.

    Note: Seasonal influenza vaccines for injection are generally inactivated flu vaccines and are allowed; however intranasal influenza vaccines (e.g., Flu-Mist®) are live attenuated vaccines, and are not allowed.

17. Is or has an immediate family member (e.g., spouse, parent/legal guardian, sibling or child) who is investigational site or sponsor staff directly involved with this trial, unless prospective IRB approval (by chair or designee) is given allowing exception to this criterion for a specific subject.

5.2 Trial Treatments

The treatment to be used in this trial is outlined below in Table 2.

Table 2 Trial Treatment

Cohort	Drug	Dose/ Potency	Dose Frequency	Route of Administration	Regimen/ Treatment Period	Use
A, B, C, D	MK-3475	10 mg/kg	Q2W	IV infusion	Day 1 of each cycle	Experimental
B2	MK-3475	200 mg	Q3W	IV infusion	Day 1 of each cycle	Experimental

Trial treatment should begin on the day of randomization or as close as possible to the date on which treatment is allocated/assigned.

5.2.1 Dose Selection/Modification

5.2.1.1 Dose Selection

The rationale for selection of doses to be used in this trial is provided in Section 4.0 – Background and Rationale.

For Cohorts A, B, C, and D, the dose amount required to prepare the MK-3475 infusion solution will be based on the subject's weight in kilograms (kg). Details on the dose calculation, preparation and administration are provided in the Pharmacy Manual.

For Cohort B2, subjects will be given 200 mg Q3W.

5.2.1.2 Dose Modification

Adverse events (both non-serious and serious) associated with pembrolizumab exposure may represent an immunologic etiology. These adverse events may occur shortly after the first dose or several months after the last dose of treatment. Pembrolizumab must be withheld for drug-related toxicities and severe or life-threatening AEs as per 3 below. See Section 5.6 and the Events of Clinical Interest Guidance Document for supportive care guidelines, including use of corticosteroids.

Table 3 Dose Modification Guidelines for Drug-Related Adverse Events.

Toxicity	Hold Treatment For Grade	Timing for Restarting Treatment	Treatment Discontinuation
Diarrhea/Colitis	2-3	Toxicity resolves to Grade 0-1.	Toxicity does not resolve within 12 weeks of last dose or inability to reduce corticosteroid to 10 mg or less of prednisone or equivalent per day within 12 weeks.
	4	Permanently discontinue	Permanently discontinue
AST, ALT, or Increased Bilirubin	2	Toxicity resolves to Grade 0-1	Toxicity does not resolve within 12 weeks of last dose.
	3-4	Permanently discontinue (see exception below)[1]	Permanently discontinue
Type 1 diabetes mellitus (if new onset) or Hyperglycemia	T1DM or 3-4	Hold pembrolizumab for new onset Type 1 diabetes mellitus or Grade 3-4 hyperglycemia associated with evidence of beta cell failure.	Resume pembrolizumab when patients are clinically and metabolically stable.
Hypophysitis	2-4	Toxicity resolves to Grade 0-1. Therapy with pembrolizumab can be continued while endocrine replacement therapy is instituted	Toxicity does not resolve within 12 weeks of last dose or inability to reduce corticosteroid to 10 mg or less of prednisone or equivalent per day within 12 weeks.
Hyperthyroidism	3	Toxicity resolves to Grade 0-1	Toxicity does not resolve within 12 weeks of last dose or inability to reduce corticosteroid to 10 mg or less of prednisone or equivalent per day within 12 weeks.
	4	Permanently discontinue	Permanently discontinue
Hypothyroidism		Therapy with pembrolizumab can be continued while thyroid replacement therapy is instituted	Therapy with pembrolizumab can be continued while thyroid replacement therapy is instituted.
Infusion Reaction	2[2]	Toxicity resolves to Grade 0-1	Permanently discontinue if toxicity develops despite adequate premedication
	3-4	Permanently discontinue	Permanently discontinue
Pneumonitis	2	Toxicity resolves to Grade 0-1	Toxicity does not resolve within 12 weeks of last dose or inability to reduce corticosteroid to 10 mg or less of prednisone or equivalent per day within 12 weeks.
	3-4	Permanently discontinue	Permanently discontinue
Renal Failure or Nephritis	2	Toxicity resolves to Grade 0-1	Toxicity does not resolve within 12 weeks of last dose or inability to reduce corticosteroid to 10 mg or less of prednisone or equivalent per day within 12 weeks.
	3-4	Permanently discontinue	Permanently discontinue
All Other Drug-Related Toxicity[3]	3 or Severe	Toxicity resolves to Grade 0-1	Toxicity does not resolve within 12 weeks of last dose or inability to reduce corticosteroid to 10 mg or less of prednisone or equivalent per day within 12 weeks.
	4	Permanently discontinue	Permanently discontinue

Note: Permanently discontinue for any severe or Grade 3 drug-related AE that recurs or any life-threatening event.

[1] For patients with liver metastasis who begin treatment with Grade 2 AST or ALT, if AST or ALT increases by greater than or equal to 50% relative to baseline and lasts for at least 1 week then patients should be discontinued.

[2] If symptoms resolve within one hour of stopping drug infusion, the infusion may be restarted at 50% of the original infusion rate (e.g., from 100 mL/hr to 50 mL/hr). Otherwise dosing will be held until symptoms resolve and the subject should be premedicated for the next scheduled dose; Refer to Table 5– Infusion Reaction Treatment Guidelines for further management details.

[3] Patients with intolerable or persistent Grade 2 drug-related AE may hold study medication at physician discretion. Permanently discontinue study drug for persistent Grade 2 adverse reactions for which treatment with study drug has been held, that do not recover to Grade 0-1 within 12 weeks of the last dose.

In case toxicity does not resolve to Grade 0-1 within 12 weeks after last infusion, trial treatment should be discontinued after consultation with the Sponsor. With investigator and Sponsor agreement, subjects with a laboratory adverse event still at Grade 2 after 12 weeks may continue treatment in the trial only if asymptomatic and controlled. For information on the management of adverse events, see Section 5.6.1.

5.2.2 Timing of Dose Administration

Trial treatment should be administered on Day 1 of each cycle after all procedures/assessments have been completed as detailed on the Trial Flow Chart (Section 6.0). Trial treatment may be administered up to 3 days before or after the scheduled Day 1 of each cycle due to administrative reasons.

All trial treatments will be administered on an outpatient basis.

Dosing interruptions are permitted in the case of medical / surgical events or logistical reasons not related to study therapy (e.g., elective surgery, unrelated medical events, subject vacation, and/or holidays). Subjects should be placed back on study therapy within 3 weeks of the scheduled interruption, unless otherwise discussed with the Sponsor. The reason for interruption should be documented in the patient's study record

MK-3475 will be administered as a 30 minute IV infusion every 2 or 3 weeks depending on the Cohort the subjects have been enrolled into. Sites should make every effort to target infusion timing to be as close to 30 minutes as possible. However, given the variability of infusion pumps from site to site, a window of -5 minutes and +10 minutes is permitted (i.e., infusion time is 30 minutes: -5 min/+10 min).

The Pharmacy Manual contains specific instructions for MK-3475 dose calculation, reconstitution, preparation of the infusion fluid, and administration.

Treatment after initial evidence of radiologic disease progression

Immunotherapeutic agents such as MK-3475 may produce antitumor effects by potentiating endogenous cancer-specific immune responses. The response patterns seen with such an approach may extend beyond the typical time course of responses seen with cytotoxic agents, and can manifest as a clinical response after an initial increase in tumor burden or even the appearance of new lesions.

If radiologic imaging shows PD, tumor assessment should be repeated ≥ 4 weeks later in order to confirm PD with the option of continuing treatment per below while awaiting radiologic confirmation of progression. If repeat imaging shows a reduction in the tumor burden compared to the initial scan demonstrating PD, treatment may be continued as per treatment calendar. If repeat imaging confirms progressive disease, subjects will be discontinued from study therapy (exception noted in Section 7.1.2.5.1). In determining whether or not the tumor burden has increased or decreased, Investigators should consider all target lesions as well as non-target lesions (please refer to the Site Imaging Manual).

When feasible, subjects should not be discontinued until progression is confirmed; however, the decision to continue study treatment after the 1st evidence of disease progression is at the Investigator's discretion based on the clinical status of the subject as described in Table 4 below. Subjects may receive study treatment while waiting for confirmation of PD if they are clinically stable as defined by the following criteria:

- Absence of signs and symptoms (including worsening of laboratory values) indicating disease progression
- No decline in ECOG performance status
- Absence of rapid progression of disease
- Absence of progressive tumor at critical anatomical sites (e.g., cord compression) requiring urgent alternative medical intervention

Table 4 Imaging and Treatment After 1st Radiologic Evidence of PD

	**Clinically Stable**		**Clinically Unstable**	
	**Imaging**	**Treatment**	**Imaging**	**Treatment**
1st radiologic evidence of PD	Repeat imaging at ≥ 4 weeks to confirm PD	May continue study treatment at the Investigator's discretion while awaiting confirmatory scan	Repeat imaging at ≥ 4 weeks to confirm PD if possible	Discontinue treatment
Repeat scan confirms PD	No additional imaging required	Discontinue treatment (exception noted in Section 7.1.2.5.1)	No additional imaging required	N/A
Repeat scan shows SD, PR or CR	Continue regularly scheduled imaging assessments every 8 weeks	Continue study treatment at the Investigator's discretion	Continue regularly scheduled imaging assessments every 8 weeks	May restart study treatment if condition has improved and/or clinically stable per Investigator's discretion

5.2.3 Trial Blinding/Masking

This is an open-label trial; therefore, the Sponsor, investigator and subject will know the treatment administered.

PD-L1 IHC biomarker testing is used to determine study eligibility for Cohorts A, B, C, and D and therefore the subject, investigator and SPONSOR will know the PD-L1 IHC result. Access to PD-L1 subject-level biomarker results for subjects enrolled in Cohort B2 will be limited to an unblinded SPONSOR statistician, and unblinded SPONSOR statistical programmer who will be responsible for data review to ensure validity of results and summary reporting of clinical response by biomarker status, but who will have no other responsibilities associated with the study.

5.3 Randomization or Treatment Allocation

Subjects participating in this trial will be allocated to trial treatment by non-random assignment.

5.4 Stratification

No stratification based on age, sex or other characteristics will be used in this trial.

5.5 Concomitant Medications/Vaccinations (allowed & prohibited)

Medications or vaccinations specifically prohibited in the exclusion criteria are not allowed during the ongoing trial. Listed below are some specific restrictions for concomitant therapy or vaccination during the course of the trial. If there is a clinical indication for one of these or other medications or vaccinations specifically prohibited during the trial, discontinuation from trial therapy or vaccination may be required. The investigator should discuss any questions regarding this with the local Clinical Monitor. The final decision on any supportive therapy or vaccination rests with the investigator and/or the subject's primary physician. However, the decision to continue the subject on trial therapy or vaccination schedule requires the mutual agreement of the investigator, the Sponsor and the subject.

5.5.1 Acceptable Concomitant Medications

All treatments that the investigator considers necessary for a subject’s welfare may be administered at the discretion of the investigator in keeping with the community standards of medical care. All concomitant medication will be recorded on the case report form (CRF) including all prescription, over-the-counter (OTC), herbal supplements, and IV medications and fluids. If changes occur during the trial period, documentation of drug dosage, frequency, route, and date may also be included on the CRF.

All concomitant medications received within 28 days before the first dose of trial treatment and 30 days after the last dose of trial treatment should be recorded. Concomitant medications administered after 30 days after the last dose of trial treatment should be recorded for SAEs and ECIs as defined in Section 7.2.

5.5.2 Prohibited Concomitant Medications

Subjects are prohibited from receiving the following therapies during the Screening and Treatment Phase (including retreatment for post-complete response relapse) of this trial:

- Antineoplastic systemic chemotherapy or biological therapy
- Immunotherapy not specified in this protocol
- Chemotherapy not specified in this protocol
- Investigational agents other than MK-3475
- Radiation therapy
    - Note: Radiation therapy to a symptomatic solitary lesion or to the brain may be allowed after consultation with Sponsor.
- Live vaccines within 30 days prior to the first dose of trial treatment and while participating in the trial. Examples of live vaccines include, but are not limited to, the following: measles, mumps, rubella, chicken pox, yellow fever, , rabies, BCG, and typhoid (oral) vaccine. Seasonal influenza vaccines for injection are generally inactivated flu vaccines and are allowed; however intranasal influenza vaccines (e.g., Flu-Mist®) are live attenuated vaccines, and are not allowed.
- Glucocorticoids for any purpose other than to modulate acute symptoms from an adverse event. The use of physiologic doses for subjects requiring ongoing corticosteroids, may be approved after consultation with the Sponsor.

Subjects who, in the assessment by the investigator, require the use of any of the aforementioned treatments for clinical management should be removed from the trial. Subjects may receive other medications that the investigator deems to be medically necessary.

The Exclusion Criteria describes other medications which are prohibited in this trial.

There are no prohibited therapies during the Post-Treatment Follow-up Phase.

5.6 Rescue Medications & Supportive Care

5.6.1 Supportive Care Guidelines

Subjects should receive appropriate supportive care measures as deemed necessary by the treating investigator. Suggested supportive care measures for the management of adverse events with potential immunologic etiology are outlined below and in greater detail in the ECI guidance document. Where appropriate, these guidelines include the use of oral or intravenous treatment with corticosteroids as well as additional anti-inflammatory agents if symptoms do not improve with administration of corticosteroids. Note that several courses of steroid tapering may be necessary as symptoms may worsen when the steroid dose is decreased. For each disorder, attempts should be made to rule out other causes such as metastatic disease or bacterial or viral infection, which might require additional supportive care. The treatment guidelines are intended to be applied when the investigator determines the events to be related to pembrolizumab.

Note: if after the evaluation the event is determined not to be related, the investigator is instructed to follow the ECI reporting guidance but does not need to follow the treatment guidance (as outlined in the ECI guidance document). Refer to Section 5.2.1 for dose modification.

It may be necessary to perform conditional procedures such as bronchoscopy, endoscopy, or skin photography as part of evaluation of the event. Suggested conditional procedures, as appropriate, can be found in the ECI guidance document.

- **Pneumonitis**:
  - For **Grade 2 events**, treat with systemic corticosteroids. When symptoms improve to Grade 1 or less, steroid taper should be started and continued over no less than 4 weeks.
  - For **Grade 3-4 events**, immediately treat with intravenous steroids. Administer additional anti-inflammatory measures, as needed.
  - Add prophylactic antibiotics for opportunistic infections in the case of prolonged steroid administration.
- **Diarrhea/Colitis**:

  Subjects should be carefully monitored for signs and symptoms of enterocolitis (such as diarrhea, abdominal pain, blood or mucus in stool, with or without fever) and of bowel perforation (such as peritoneal signs and ileus).

  - All subjects who experience diarrhea/colitis should be advised to drink liberal quantities of clear fluids. If sufficient oral fluid intake is not feasible, fluid and electrolytes should be substituted via IV infusion. For Grade 2 or higher diarrhea, consider GI consultation and endoscopy to confirm or rule out colitis.

  - For **Grade 2 diarrhea/colitis** that persists greater than 3 days, administer oral corticosteroids.
  - For **Grade 3 or 4 diarrhea/colitis** that persists > 1 week, treat with intravenous steroids followed by high dose oral steroids.
  - When symptoms improve to Grade 1 or less, steroid taper should be started and continued over no less than 4 weeks.

- **Type 1 diabetes mellitus (if new onset, including diabetic ketoacidosis [DKA]) or ≥ Grade 3 Hyperglycemia, if associated with ketosis (ketonuria) or metabolic acidosis (DKA)**
  - For **T1DM** or **Grade 3-4** Hyperglycemia
    - Insulin replacement therapy is recommended for Type I diabetes mellitus and for Grade 3-4 hyperglycemia associated with metabolic acidosis or ketonuria.
    - Evaluate patients with serum glucose and a metabolic panel, urine ketones, glycosylated hemoglobin, and C-peptide.

- **Hypophysitis**:
  - For **Grade 2** events, treat with corticosteroids. When symptoms improve to Grade 1 or less, steroid taper should be started and continued over no less than 4 weeks. Replacement of appropriate hormones may be required as the steroid dose is tapered.
  - For **Grade 3-4** events, treat with an initial dose of IV corticosteroids followed by oral corticosteroids. When symptoms improve to Grade 1 or less, steroid taper should be started and continued over no less than 4 weeks. Replacement of appropriate hormones may be required as the steroid dose is tapered.

- **Hyperthyroidism or Hypothyroidism**:

  Thyroid disorders can occur at any time during treatment. Monitor patients for changes in thyroid function (at the start of treatment, periodically during treatment, and as indicated based on clinical evaluation) and for clinical signs and symptoms of thyroid disorders.

  - **Grade 2** hyperthyroidism events (and **Grade 2-4** hypothyroidism):
    - In hyperthyroidism, non-selective beta-blockers (e.g. propranolol) are suggested as initial therapy.
    - In hypothyroidism, thyroid hormone replacement therapy, with levothyroxine or liothyroinine, is indicated per standard of care.

- **Grade 3-4** hyperthyroidism
    - Treat with an initial dose of IV corticosteroid followed by oral corticosteroids. When symptoms improve to Grade 1 or less, steroid taper should be started and continued over no less than 4 weeks. Replacement of appropriate hormones may be required as the steroid dose is tapered.

- **Hepatic**:
    - For **Grade 2** events, monitor liver function tests more frequently until returned to baseline values (consider weekly).
        - Treat with IV or oral corticosteroids
    - For **Grade 3-4** events, treat with intravenous corticosteroids for 24 to 48 hours.
    - When symptoms improve to Grade 1 or less, a steroid taper should be started and continued over no less than 4 weeks.

- **Renal Failure or Nephritis**:
    - For **Grade 2** events, treat with corticosteroids.
    - For **Grade 3-4** events, treat with systemic corticosteroids.
    - When symptoms improve to Grade 1 or less, steroid taper should be started and continued over no less than 4 weeks.

- **Management of Infusion Reactions**: Signs and symptoms usually develop during or shortly after drug infusion and generally resolve completely within 24 hours of completion of infusion.

  Table 5 below shows treatment guidelines for subjects who experience an infusion reaction associated with administration of pembrolizumab (MK-3475).

Table 5 Infusion Reaction Treatment Guidelines

<table>
<tr><th>NCI CTCAE Grade</th><th>Treatment</th><th>Premedication at subsequent dosing</th></tr>
<tr><td><u>Grade 1</u>
Mild reaction; infusion interruption not indicated; intervention not indicated</td><td>Increase monitoring of vital signs as medically indicated until the subject is deemed medically stable in the opinion of the investigator.</td><td>None</td></tr>
<tr><td><u>Grade 2</u>
Requires infusion interruption but responds promptly to symptomatic treatment (e.g., antihistamines, NSAIDS, narcotics, IV fluids); prophylactic medications indicated for < =24 hrs</td><td>Stop Infusion and monitor symptoms.
Additional appropriate medical therapy may include but is not limited to:
IV fluids
Antihistamines
NSAIDS
Acetaminophen
Narcotics
Increase monitoring of vital signs as medically indicated until the subject is deemed medically stable in the opinion of the investigator.
If symptoms resolve within one hour of stopping drug infusion, the infusion may be restarted at 50% of the original infusion rate (e.g., from 100 mL/hr to 50 mL/hr). Otherwise dosing will be held until symptoms resolve and the subject should be premedicated for the next scheduled dose.
Subjects who develop Grade 2 toxicity despite adequate premedication should be permanently discontinued from further trial treatment administration.</td><td>Subject may be premedicated 1.5h (± 30 minutes) prior to infusion of pembrolizumab (MK-3475) with:

Diphenhydramine 50 mg po (or equivalent dose of antihistamine).

Acetaminophen 500-1000 mg po (or equivalent dose of antipyretic).</td></tr>
<tr><td><u>Grades 3 or 4</u>

Grade 3:
Prolonged (i.e., not rapidly responsive to symptomatic medication and/or brief interruption of infusion); recurrence of symptoms following initial improvement; hospitalization indicated for other clinical sequelae (e.g., renal impairment, pulmonary infiltrates)

Grade 4:
Life-threatening; pressor or ventilatory support indicated</td><td>Stop Infusion.
Additional appropriate medical therapy may include but is not limited to:
IV fluids
Antihistamines
NSAIDS
Acetaminophen
Narcotics
Oxygen
Pressors
Corticosteroids
Epinephrine

Increase monitoring of vital signs as medically indicated until the subject is deemed medically stable in the opinion of the investigator.
Hospitalization may be indicated.
Subject is permanently discontinued from further trial treatment administration.</td><td>No subsequent dosing</td></tr>
<tr><td colspan="3">Appropriate resuscitation equipment should be available in the room and a physician readily available during the period of drug administration.</td></tr>
</table>

5.7 Diet/Activity/Other Considerations

5.7.1 Diet

Subjects should maintain a normal diet unless modifications are required to manage an AE such as diarrhea, nausea or vomiting.

5.7.2 Contraception

MK-3475 may have adverse effects on a fetus in utero. Furthermore, it is not known if MK-3475 has transient adverse effects on the composition of sperm. Non-pregnant, non-breast-feeding women may be enrolled if they are willing to use 2 methods of birth control or are considered highly unlikely to conceive. Highly unlikely to conceive is defined as 1) surgically sterilized, or 2) postmenopausal (a woman who is ≥45 years of age and has not had menses for greater than 1 year will be considered postmenopausal), or 3) not heterosexually active for the duration of the study. The two birth control methods can either be two barrier methods or a barrier method plus a hormonal method to prevent pregnancy. Subjects should start using birth control from study Visit 1 throughout the study period up to 120 days after the last dose of study therapy.

The following are considered adequate barrier methods of contraception: diaphragm, condom (by the partner), copper intrauterine device, sponge, or spermicide. Appropriate hormonal contraceptives will include any registered and marketed contraceptive agent that contains an estrogen and/or a progestational agent (including oral, subcutaneous, intrauterine, or intramuscular agents).

Subjects should be informed that taking the study medication may involve unknown risks to the fetus (unborn baby) if pregnancy were to occur during the study. In order to participate in the study they must adhere to the contraception requirement (described above) for the duration of the study and during the follow-up period defined in section 7.2.2-Reporting of Pregnancy and Lactation to the Sponsor. If there is any question that a subject will not reliably comply with the requirements for contraception, that subject should not be entered into the study.

5.7.3 Use in Pregnancy

If a subject inadvertently becomes pregnant while on treatment with MK-3475, the subject will immediately be removed from the study. The site will contact the subject at least monthly and document the subject's status until the pregnancy has been completed or terminated. The outcome of the pregnancy will be reported to the Sponsor without delay and within 24 hours if the outcome is a serious adverse experience (e.g., death, abortion, congenital anomaly, or other disabling or life-threatening complication to the mother or newborn). The study investigator will make every effort to obtain permission to follow the outcome of the pregnancy and report the condition of the fetus or newborn to the Sponsor. If a male subject impregnates his female partner the study personnel at the site must be informed immediately and the pregnancy reported to the Sponsor and followed as described above and in Section 7.2.2.

5.7.4 Use in Nursing Women

It is unknown whether MK-3475 is excreted in human milk. Since many drugs are excreted in human milk, and because of the potential for serious adverse reactions in the nursing infant, subjects who are breast-feeding are not eligible for enrollment.

5.8 Subject Withdrawal/Discontinuation Criteria

Subjects may withdraw consent at any time for any reason or be dropped from the trial at the discretion of the investigator should any untoward effect occur. In addition, a subject may be withdrawn by the investigator or the Sponsor if enrollment into the trial is inappropriate, the trial plan is violated, or for administrative and/or other safety reasons. Specific details regarding discontinuation or withdrawal procedures; including specific details regarding withdrawal from Future Biomedical Research, are provided in Section 7.1.4 – Other Procedures.

A subject must be discontinued from the trial for any of the following reasons:

1. The subject or legal representative (such as a parent or legal guardian) withdraws consent.

A subject must be discontinued from treatment (but may continue to be monitored in the post-treatment follow-up portion of the trial) for any of the following reasons:

- The subject or legal representative (such as a parent or legal guardian) withdraws consent for treatment
- Confirmed radiographic disease progression

  Note: For unconfirmed radiographic disease progression, please see Section 5.2.2.

  Note: A subject may be granted an exception to continue on treatment with confirmed radiographic progression if clinically stable or clinically improved, please see Section 7.1.2.5.1.
- Unacceptable adverse experiences as described in Section 5.2.1.2
- Intercurrent illness that prevents further administration of treatment
- Investigator's decision to withdraw the subject
- The subject has a confirmed positive serum pregnancy test
- Noncompliance with trial treatment or procedure requirements
- The subject is lost to follow-up
- Completed 24 months of treatment with MK-3475

  Note: 24 months of study medication is calculated from the date of first dose. Subjects who stop MK-3475 after 24 months may be eligible for up to one year of additional study treatment if they progress after stopping study treatment provided they meet the requirements detailed in Section 7.1.5.2.1.

- Administrative reasons

The End of Treatment and Follow-up visit procedures are listed in Section 6 (Protocol Flow Chart) and Section 7.1.5 (Visit Requirements). After the end of treatment, each subject will be followed for 30 days for adverse event monitoring (serious adverse events will be collected for 90 days after the end of treatment or 30 days after the end of treatment if the subject initiates new anticancer therapy, whichever is earlier, as described in Section 7.2.3.1). Subjects who discontinue for reasons other than progressive disease will have post-treatment follow-up for disease status until disease progression, initiating a non-study cancer treatment, withdrawing consent or becoming lost to follow-up. After documented disease progression each subject will be followed by telephone for overall survival until death, withdrawal of consent, or the end of the study, whichever occurs first.

5.8.1 Discontinuation of Study Therapy after CR

Discontinuation of treatment may be considered for subjects who have attained a confirmed CR that have been treated for at least 24 weeks with MK-3475 and had at least two treatments with MK-3475 beyond the date when the initial CR was declared. Subjects who then experience radiographic disease progression may be eligible for up to one year of additional treatment with MK-3475 at the discretion of the investigator if no cancer treatment was administered since the last dose of MK-3475, the subject meets the safety parameters listed in the Inclusion/Exclusion criteria, and the trial is open. Subjects will resume therapy at the same dose and schedule at the time of initial discontinuation. Additional details are provided in Section 7.1.5.2.1.

5.9 Subject Replacement Strategy

Additional subjects may be enrolled in a given cohort to ensure that the required number of evaluable subjects in each cohort is achieved. A subject that discontinues the trial for progressive disease or a drug-related AE will not be replaced and will be counted in the evaluable population of subjects for the respective cohort. Further details are provided in Section 8.1.3.

5.10 Beginning and End of the Trial

The study begins when the first subject signs the informed consent (either pre-screening consent or main study consent). The end of the study may be designated as the time point when all subjects have discontinued the study or are a minimum of 6 months post initial study medication administration or all subjects still on study have received their 24-week scan (i.e. completed their third 8-week efficacy assessment). If, by the end of the study, there remains at least 1 subject still on study treatment for at least 6 months, the subject(s) may enter additional treatment cycles. At this point a database lock of the trial may occur to allow the analysis of the study data. Any remaining subjects may continue to receive study medication and be seen by the investigator per usual standard of care for this subject population. In addition, the investigator will be expected to monitor for and report any serious adverse events, events of clinical interest, and pregnancies, as detailed in Section 7.2.3 (Serious Adverse Experiences). The subject is considered on study until such time that he/she meets any of the discontinuation criteria and written notification is given to the Sponsor.

5.11 Clinical Criteria for Early Trial Termination

Early trial termination will be the result of the criteria specified below:

1. Quality or quantity of data recording is inaccurate or incomplete

2. Poor adherence to protocol and regulatory requirements

3. Incidence or severity of adverse drug reaction in this or other studies indicates a potential health hazard to subjects

4. Plans to modify or discontinue the development of the study drug

In the event of Sponsor decision to no longer supply study drug, ample notification will be provided so that appropriate adjustments to subject treatment can be made.

6.0 TRIAL FLOW CHART

6.1 Study Flow Chart for Cohorts A, B, C and D (10 mg/kg Q2W dosing)

**Trial Period:**	**Screening Phase**		**Treatment Cycles[a]**								**End of Treatment**	**Post-Treatment**		
							To be repeated beyond 8 cycles							
Treatment Cycle/Title:	Pre-screening (Visit 1)[b]	Main Study Screening (Visit 2)[c]	1	2	3	4	5	6	7	8	Discon	Post-Treatment Safety Follow-up	Follow Up Visits[d]	Survival Follow-Up[e]
Scheduling Window (Days)[f]:		-28 to -1		± 3	± 3	± 3	± 3	± 3	± 3	± 3	At time of Discon	30 days post discon	Every 8 weeks post discon	Every 12 weeks
**Administrative Procedures**														
Pre-screening Consent	X[g]													
Informed Consent		X[h]												
Informed Consent for Future Biomedical Research		X[i]												
Inclusion/Exclusion Criteria		X												
Subject Identification Card		X												
Demographics and Medical History		X												
Prior and Concomitant Medication Review		X[j]	X	X	X	X	X	X	X	X	X	X[j]		
Trial Treatment Administration			X	X	X	X	X	X	X	X				
Post-study anticancer therapy status													X	X
Survival Status														X
**Clinical Procedures/Assessments**														
Review Adverse Events[k]		X	X	X	X	X	X	X	X	X	X	X[l]	X[l]	
Full Physical Examination		X					X[m]							
Directed Physical Examination			X	X	X	X		X[ff]	X[ff]	X[ff]	X			
Vital Signs and Weight[n]		X	X	X	X	X	X	X	X	X	X			
ECOG Performance Status		X	X	X	X	X	X	X[gg]	X[gg]	X[gg]	X			
**Laboratory Procedures/Assessments: analysis performed by local laboratory**														
Pregnancy Test – Urine or Serum . -HCGo		X												
PT/INR and aPTT[p]		X[q]												
CBC with Differential[r]		X[q]		X	X	X	X	X	X	X	X	X[s]		
Comprehensive Chemistry Panel[r]		X[q]		X	X	X	X	X	X	X	X	X[s]		
Urinalysis[r]		X[q]					X[m]					X[s]		

Trial Period:	Screening Phase		Treatment Cycles[a]								End of Treatment	Post-Treatment		
							To be repeated beyond 8 cycles							
Treatment Cycle/Title:	Pre-screening (Visit 1)[b]	Main Study Screening (Visit 2)[c]	1	2	3	4	5	6	7	8	Discon	Post-Treatment Safety Follow-up	Follow Up Visits[d]	Survival Follow-Up[e]
Scheduling Window (Days)[f]:		-28 to -1		± 3	± 3	± 3	± 3	± 3	± 3	± 3	At time of Discon	30 days post discon	Every 8 weeks post discon	Every 12 weeks
T3, FT4 and TSH[r]		X[q]					X[m]					X[s]		
**Laboratory Procedures/Assessments: analysis performed by central laboratory**														
Anti-MK-3475 Antibodies[t]			X[t]	X[t]			X[t]					X[t]		
Pharmacokinetics[t]			X[t,u]	X[t]			X[t]					X[t]		
Blood for Future Biomedical Research[v]			X											
**Efficacy Measurements**														
Tumor Imaging[w,x]		X					X				X[y]		X[d]	
**Tumor Biopsies/Archival Tissue Collection/Correlative Studies Blood**														
Archival Tissue Collection[z]	X[aa]													
Cohort B (H/N) and Cohort D (gastric cancer) Tumor Tissue Collection[z]		X[aa,bb]					X[bb]				X[cc]			
Cohort A (TNBC) and Cohort C (urothelial tract cancer) Tumor Tissue Collection[z]		X[aa,dd]					X[dd]				X[cc]			
Correlative Studies Blood Collection			X[ee]				X[ee]				X[ee]			

a. In general, assessments/procedures are to be performed on Day 1 and prior to the first dose of treatment for each cycle unless otherwise specified. Treatment cycles are 2 weeks. Imaging should always be performed every 8 weeks (56 days ± 7 days) regardless of any treatment delays.
b. At the pre-screening visit, subjects will sign the pre-screening consent and submit an archival sample for PD-L1 characterization.
c. Subjects who submit an archival tumor sample at the prescreening visit and are found to be PD-L1 positive will continue to the screening portion of the study. Subjects who do not have an archival sample will go directly to the screening phase of the study.
d. In subjects who discontinue study therapy without documented disease progression, every effort should be made to continue monitoring their disease status by radiologic imaging[x] every 8 weeks (± 7 days) until (1) the start of new anti-cancer treatment, (2) documented disease progression, (3) death, or (4) the end of the study, whichever occurs first.
e. After the start of new anti-cancer treatment or documented disease progression, the subject should be contacted by telephone every 12 weeks to assess for survival status.
f. In general, the window for each visit is ± 3 days unless otherwise noted.
g. Pre-screening informed consent must be obtained prior to sending an archival sample to the lab for characterization. Subjects that do not have archival tissue available to send must sign the main study consent prior to undergoing a newly obtained biopsy.
h. Written consent must be obtained prior to performing any protocol specific procedure. Results of a test performed prior to the subject signing consent as part of routine clinical management are acceptable in lieu of a screening test if performed within the specified time frame (e.g., within 28 days prior to the first dose of trial treatment). Screening number will be assigned when the study informed consent is signed.
i. Signing the informed consent for future biomedical research (FBR) samples is optional. Detailed instructions for the collection and management of specimens for FBR are provided in the Procedures Manual and Section 12.2.
j. Prior medications – Record all medications taken within 28 days of screening visit. Concomitant medications – Enter new medications started during the trial through the Safety Follow-up visit. Record all medications taken for SAEs as defined in Section 7.2.
k. AEs and laboratory safety measurements will be graded per NCI CTCAE version 4.0. All AEs, whether gradable by CTCAE or not, will also be evaluated for seriousness.
l. Record all AEs occurring within 30 days after the last dose of trial treatment. Report all SAEs (related and unrelated to trial treatment) and ECIs occurring up until 90 days after the last dose of trial treatment or 30 days after the end of treatment if the subject initiates new anticancer therapy, whichever is earlier. Afterwards, report only SAEs and ECIs that are related to trial treatment.
m. To be repeated every 4 cycles after cycle 5.
n. Vital signs to include temperature, pulse, respiratory rate, weight and blood pressure. Height will be measured at visit 2 only.
o. For women of reproductive potential, a urine pregnancy test should be performed within 72 hours prior to first dose of trial treatment. If urine pregnancy results cannot be confirmed as negative, a serum pregnancy test performed by the local study site laboratory will be required. Pregnancy tests (serum and/or urine tests) should be repeated if required by local guidelines.
p. Coagulation factors (PT/INR and aPTT) should be tested as part of the screening procedures for all subjects. Any subject receiving anticoagulant therapy should have coagulation factors monitored closely throughout the trial.
q. Laboratory tests for screening are to be performed within 10 days prior to the first dose of trial treatment. See Section 7.1.3 for details regarding laboratory tests.
r. After Cycle 1, lab samples can be collected up to 72 hours prior to the scheduled time point. See Section 7.1.3 for details regarding laboratory tests.
s. Unresolved abnormal labs that are drug related AEs should be followed until resolution. Labs do not need to be repeated after the end of treatment if labs are within normal range.
t. Pre-dose trough and post-dose peak PK samples will be collected at Cycles 1 and 2. Pre-dose trough samples only will be collected every 4 cycles starting with Cycle 5 and through Cycle 37, 30 days after discontinuation of study drug, and 3 months and 6 months after discontinuation of study drug (or until the subject starts new anti-neoplastic therapy). All trough samples should be drawn within 24 hours before infusion of MK-3475. All peak samples should be drawn within 30 minutes after the end of the infusion. Anti-MK-3475 antibodies should be drawn with all pre-dose trough PK samples, the 30 day discontinuation draw and 3 months and 6 months after discontinuation of study drug (or until the subject starts new anti-neoplastic therapy). Procedures for sample collection are described in the Procedures Manual.
u. An additional single PK sample should be drawn between 24 to 96 hours after Cycle 1 dosing.
v. Informed consent for future biomedical research samples must be obtained before the DNA sample. DNA sample for analysis should be obtained predose, on Day 1 (or with the next scheduled blood draw) as the last sample drawn or at a later date as soon as the informed consent is obtained. Detailed instructions for the collection and management of specimens for FBR are provided in the Procedures Manual and Section 12.2.

w.	The initial tumor imaging will be performed within 28 days prior to the first dose of trial treatment. Scans performed as part of routine clinical management are acceptable for use as the screening scan if they are of diagnostic quality and performed within 28 days prior to the first dose of trial treatment. On-study imaging will be performed every 8 weeks (± 7 days) after the first dose of trial treatment or more frequently if clinically indicated. Imaging timing should follow calendar days and should not be adjusted for delays in cycle starts or extension of MK-3475 cycle frequencies. The same imaging technique should be used in a subject throughout the trial. Local reading (investigator assessment with site radiology reading) will be used to determine eligibility and for subject management; Sponsor will collect radiological assessments for retrospective analysis by an independent central radiology review vendor. The processes for image collection and transmission to the independent central radiology reviewl vendor are in the Site Imaging Manual.
x.	Per the modified RECIST 1.1 used in this protocol, if imaging shows progressive disease, the imaging assessment should be performed at a minimum of 4 weeks later in order to confirm progressive disease as described in Section 4.2.3.1. Please refer to the Procedure Manual for additional details on modifications to RECIST.
y.	In subjects who discontinue study therapy without confirmed disease progression, a radiological evaluation should be performed at the time of treatment discontinuation (i.e. date of discontinuation ± 4 week window). If a previous scan was obtained within 4 weeks prior to the date of discontinuation, then a scan at treatment discontinuation isn't mandatory.
z.	Detailed instructions for tissue collection, processing and shipment are provided in the Procedures Manual. If the subject signs the Future Biomedical Research (FBR) consent, any leftover tissue that would ordinarily be discarded at the end of the main study will be retained for FBR.
aa.	Baseline tumor tissue for biomarker analysis from an archival tissue sample or newly obtained core or excisional biopsy (FNA not adequate) of a tumor lesion not previously irradiated must be provided and received by the independent central radiology reviewl vendor before enrollment for characterization of PD-L1 status. These samples are not required to be obtained within 28 days of enrollment. Subjects with H/N cancer may provide tissue from a previously irradiated lesion.
bb.	Newly obtained tumor biopsy is required for subjects enrolled into Cohort B (H/N cancer) and Cohort D (gastric cancer) of the study. Tumor biopsies that are inaccessible or contraindicated due to subject safety concerns are exempt from this requirement. The pre-dose newly obtained biopsy is not required for PD-L1 characterization and may be performed just prior to the first dose of study treatment after all eligibility criteria has been met.
cc.	Tumor biopsy for clinically stable subjects at treatment discontinuation is highly encouraged.
dd.	Tumor biopsy is highly encouraged for all subjects. If activity within the Cohort is observed (at least 2 responders within the Cohort) the tumor biopsy will become mandatory. Tumor biopsies that are inaccessible or contraindicated due to subject safety concerns are exempt from this requirement. The pre-dose newly obtained biopsy is not required for PD-L1 characterization and may be performed just prior to the first dose of study treatment after all eligibility criteria has been met
ee.	Blood for correlative studies should be collected prior to Cycle 1, at Cycle 5 and again at treatment discontinuation.
ff.	Following Cycle 8, the directed physical exam is only required at Cycle 11, 15, 19, and every 4 cycles thereafter.
gg.	Following Cycle 8, the ECOG performance status should be determined only in conjunction with a protocol-specified full or directed physical exam (Cycle 9, 11, 13, 15, 17, 19 and every 2 cycles thereafter.

6.2 Study Flow Chart for Cohort B2 (200 mg Q3W dosing)

**Trial Period:**	**Screening Phase**	**Treatment Cycles[a]**						**End of Treatment**	**Post-Treatment**		
					To be repeated beyond 6 cycles						
Treatment Cycle/Title:	Main Study Screening (Visit 2)	1	2	3	4	5	6	Discon	Post-Treatment Safety Follow-up	Follow Up Visits[b]	Survival Follow-Up[c]
Scheduling Window (Days)[d]:	-28 to -1		± 3	± 3	± 3	± 3	± 3	At time of Discon	30 days post discon	Every 8 weeks post discon	Every 12 weeks
Informed Consent	X[e]										
Informed Consent for Future Biomedical Research	X[f]										
Inclusion/Exclusion Criteria	X										
Subject Identification Card	X										
Demographics and Medical History	X										
Prior and Concomitant Medication Review	X[g]	X	X	X	X	X	X	X	X[g]		
Trial Treatment Administration		X	X	X	X	X	X				
Post-study anticancer therapy status										X	X
Survival Status											X
Review Adverse Events[h]	X	X	X	X	X	X	X	X	X[i]	X[i]	
Full Physical Examination	X				X[j]						
Directed Physical Examination		X	X	X		X[k]	X[k]	X			
Vital Signs and Weight[l]	X	X	X	X	X	X	X	X			
ECOG Performance Status	X	X	X	X	X	X[m]	X[m]	X			
Pregnancy Test – Urine or Serum β-HCG[n]	X										
PT/INR and aPTT[o]	X[p]										
CBC with Differential[q]	X[p]		X	X	X	X	X	X	X[r]		
Comprehensive Chemistry Panel[q]	X[p]		X	X	X	X	X	X	X[r]		
Urinalysis[q]	X[p]				X[j]				X[r]		
T3, FT4 and TSH[q]	X[p]				X[j]				X[r]		
Blood for Future Biomedical Research[s]		X									
Tumor Imaging[t,u]	X			X				X[v]		X[b]	
Archival Tissue Collection[w]	X[x]										
Newly Obtained Biopsy Collection[w]	X[y]			X[y]				X[z]			
Correlative Studies Blood Collection		X[aa]		X[aa]				X[aa]			

a. In general, assessments/procedures are to be performed on Day 1 and prior to the first dose of treatment for each cycle unless otherwise specified. Treatment cycles are 3 weeks; Imaging should always be performed every 8 weeks (56 days ± 7 days) regardless of any treatment delays.
b. In subjects who discontinue study therapy without documented disease progression, every effort should be made to continue monitoring their disease status by radiologic imaging[u] every 8 weeks (± 7 days) until (1) the start of new anti-cancer treatment, (2) documented disease progression, (3) death, or (4) the end of the study, whichever occurs first.
c. After the start of new anti-cancer treatment or documented disease progression, the subject should be contacted by telephone every 12 weeks to assess for survival status.
d. In general, the window for each visit is ± 3 days unless otherwise noted.
e. Written consent must be obtained prior to performing any protocol specific procedure. Results of a test performed prior to the subject signing consent as part of routine clinical management are acceptable in lieu of a screening test if performed within the specified time frame (e.g., within 28 days prior to the first dose of trial treatment). Screening number will be assigned when the study informed consent is signed.
f. Signing the informed consent for future biomedical research (FBR) samples is optional. Detailed instructions for the collection and management of specimens for FBR are provided in the Procedures Manual and Section 12.2.
g. Prior medications – Record all medications taken within 28 days of screening visit. Concomitant medications – Enter new medications started during the trial through the Safety Follow-up visit. Record all medications taken for SAEs as defined in Section 7.2.
h. AEs and laboratory safety measurements will be graded per NCI CTCAE version 4.0. All AEs, whether gradable by CTCAE or not, will also be evaluated for seriousness.
i. Record all AEs occurring within 30 days after the last dose of trial treatment. Report all SAEs (related and unrelated to trial treatment) and ECIs occurring up until 90 days after the last dose of trial treatment or 30 days after the end of treatment if the subject initiates new anticancer therapy, whichever is earlier. Afterwards, report only SAEs and ECIs that are related to trial treatment.
j. To be repeated every 3 cycles after cycle 4.
k. Following Cycle 6, the directed physical exam is only required as clinically appropriate as long as a physical exam is performed every 6 weeks.
l. Vital signs to include temperature, pulse, respiratory rate, weight and blood pressure. Height will be measured at the screening visit (visit 2) only.
m. Following Cycle 6, the ECOG performance status should be determined only in conjunction with a protocol-specified full or directed physical exam.
n. For women of reproductive potential, a urine pregnancy test should be performed within 72 hours prior to first dose of trial treatment. If urine pregnancy results cannot be confirmed as negative, a serum pregnancy test performed by the local study site laboratory will be required. Pregnancy tests (serum and/or urine tests) should be repeated if required by local guidelines.
o. Coagulation factors (PT/INR and aPTT) should be tested as part of the screening procedures for all subjects. Any subject receiving anticoagulant therapy should have coagulation factors monitored closely throughout the trial.
p. Laboratory tests for screening are to be performed within 10 days prior to the first dose of trial treatment. See Section 7.1.3 for details regarding laboratory tests.
q. After Cycle 1, lab samples can be collected up to 72 hours prior to the scheduled time point. See Section 7.1.3 for details regarding laboratory tests.
r. Unresolved abnormal labs that are drug related AEs should be followed until resolution. Labs do not need to be repeated after the end of treatment if labs are within normal range.
s. Informed consent for future biomedical research samples must be obtained before the DNA sample. DNA sample for analysis should be obtained predose, on Day 1 (or with the next scheduled blood draw) as the last sample drawn or at a later date as soon as the informed consent is obtained. Detailed instructions for the collection and management of specimens for FBR are provided in the Procedures Manual and Section 12.2.
t. The initial tumor imaging will be performed within 28 days prior to the first dose of trial treatment. Scans performed as part of routine clinical management are acceptable for use as the screening scan if they are of diagnostic quality and performed within 28 days prior to the first dose of trial treatment. On-study imaging will be performed every 8 weeks (± 7 days) after the first dose of trial treatment or more frequently if clinically indicated. Imaging timing should follow calendar days and should not be adjusted for delays in cycle starts or extension of MK-3475 cycle frequencies. The same imaging technique should be used in a subject throughout the trial. Local reading (investigator assessment with site radiology reading) will be used to determine eligibility and for subject management; Sponsor will collect radiological assessments for retrospective analysis by an independent central radiology review vendor. The processes for image collection and transmission to the independent central radiology review vendor are in the Site Imaging Manual.
u. Per the modified RECIST 1.1 used in this protocol, if imaging shows progressive disease, the imaging assessment should be performed at a minimum of 4 weeks later in order to confirm progressive disease as described in Section 4.2.3.1. Please refer to the Procedure Manual for additional details on modifications to RECIST.

v. In subjects who discontinue study therapy without confirmed disease progression, a radiological evaluation should be performed at the time of treatment discontinuation (i.e. date of discontinuation ± 4 week window). If a previous scan was obtained within 4 weeks prior to the date of discontinuation, then a scan at treatment discontinuation isn't mandatory.

w. Detailed instructions for tissue collection, processing and shipment are provided in the Procedures Manual. If the subject signs the Future Biomedical Research (FBR) consent, any leftover tissue that would ordinarily be discarded at the end of the main study will be retained for FBR.

x. Baseline tumor tissue for biomarker analysis from an archival tissue sample of a tumor lesion must be provided and submitted to the independent central radiology review vendor before enrollment These samples are not required to be obtained within 28 days of enrollment. . Exceptions to the archival tissue requirement may be granted after discussion with the Sponsor if a newly obtained biopsy is performed at baseline.

y. Newly obtained tumor biopsies are mandatory for subjects prior to Cycle 1 initiation of MK-3475 and again at Cycle 3. Exemptions to the tumor biopsy require Sponsor approval and appropriate justification..

z. Tumor biopsy for clinically stable subjects at treatment discontinuation is highly encouraged.

aa. Blood for correlative studies should be collected prior to Cycle 1, at Cycle 3 and again at treatment discontinuation.

6.3 Second Course Phase (Retreatment ONLY) for Cohorts A, B, C and D

Trial Period:	Treatment Cycles[a]								End of Treatment	Post-Treatment		
					To be repeated beyond 8 cycles							
	Second Course Phase											
Treatment Cycle/Title:	1	2	3	4	5	6	7	8	Discon	Post-Treatment Safety Follow-up	Follow Up Visits[b]	Survival Follow-Up[c]
Scheduling Window (Days)[d]:		± 3	± 3	± 3	± 3	± 3	± 3	± 3	At time of Discon	30 days post discon	Every 8 weeks post discon	Every 12 weeks
**Administrative Procedures**												
Eligibility Criteria[e]	X											
Concomitant Medication Review[f]	X	X	X	X	X	X	X	X	X	X		
Trial Treatment Administration[g]	X	X	X	X	X	X	X	X				
Post-study anticancer therapy status											X	X
Survival Status												X
**Clinical Procedures/Assessments**												
Review Adverse Events[h]	X	X	X	X	X	X	X	X	X	X[i]	X[i]	
Full Physical Examination	X				X[j]							
Directed Physical Examination		X	X	X		X[u]	X[u]	X[u]	X			
Vital Signs and Weight[k]	X	X	X	X	X	X	X	X	X			
ECOG Performance Status	X	X	X	X	X	X[v]	X[v]	X[v]	X			
**Laboratory Procedures/Assessments: analysis performed by local laboratory**												
Pregnancy Test – Urine or Serum β-HCG[l]	X											
PT/INR and aPTT[m]	X[n]											
CBC with Differential[o]	X[n]	X	X	X	X	X	X	X	X	X[s]		
Comprehensive Chemistry Panel[o]	X[n]	X	X	X	X	X	X	X	X	X[s]		
T3, FT4 and TSH[o]	X[n]				X[j]					X[s]		
**Laboratory Procedures/Assessments: analysis performed by central laboratory**												
Anti-MK-3475 Antibodies	X[t]	X[t]			X[t]					X[t]	X[t]	
Pharmacokinetics	X[t]	X[t]			X[t]					X[t]	X[t]	
**Efficacy Measurements**												
Tumor Imaging[p,q]	X				X				X[r]		X[b]	

a. In general, assessments/procedures are to be performed on Day 1 and prior to the first dose of treatment for each cycle unless otherwise specified. Treatment cycles are 2 weeks Imaging should always be performed every 8 weeks (56 days ± 7 days) regardless of any treatment delays.
b. In subjects who discontinue study therapy without documented disease progression, every effort should be made to continue monitoring their disease status by radiologic imaging[p] until (1) the start of new anti-cancer treatment, (2) documented disease progression, (3) death, or (4) the end of the study, whichever occurs first.
c. After the start of new anti-cancer treatment or documented disease progression, the subject should be contacted by telephone every 12 weeks to assess for survival status.
d. In general, the window for each visit is ± 3 days unless otherwise noted.
e. Subjects who either a) attain a CR and discontinue treatment or b) discontinue treatment after 24 months on MK-3475 for reasons other than disease progression or intolerability may restart trial treatment if they meet the criteria specified in Section 7.1.5.2.1.
f. Concomitant medications – Enter new medications started during the trial through the Safety Follow-up visit. Record all medications taken for SAEs as defined in Section 7.2.
g. Subjects who restart treatment should resume at the same dose and cycle interval which they were receiving prior to discontinuation.
h. AEs and laboratory safety measurements will be graded per NCI CTCAE version 4.0. All AEs, whether gradable by CTCAE or not, will also be evaluated for seriousness.
i. Record all AEs occurring within 30 days after the last dose of trial treatment. Report all SAEs (related and unrelated to trial treatment) and ECIs occurring up until 90 days after the last dose of trial treatment or 30 days after the end of treatment if the subject initiates new anticancer therapy, whichever is earlier. Afterwards, report only SAEs and ECIs that are related to trial treatment.
j. To be repeated every 4 cycles after cycle 5.
k. Vital signs to include temperature, pulse, respiratory rate, weight and blood pressure.
l. For women of reproductive potential, a urine pregnancy test will be performed within 72 hours prior to first dose of retreatment. If urine pregnancy results cannot be confirmed as negative, a serum pregnancy test performed by the local study site laboratory will be required. Pregnancy tests (serum and/or urine tests) should be repeated if required by local guidelines.
m. Coagulation factors (PT/INR and aPTT) should be monitored closely throughout the trial for any subject receiving anticoagulant therapy.
n. Laboratory tests for determining eligibility for retreatment are to be performed within 10 days prior to the first retreatment dose of MK-3475. See Section 7.1.3 for details regarding laboratory tests.
o. After the first dose, lab samples can be collected up to 72 hours prior to the scheduled time point. See Section 7.1.3 for details regarding laboratory tests.
p. A scan must be performed within 28 days prior to restarting treatment with MK-3475. Imaging should continue to be performed every 8 weeks (56 ± 7 days) from the first dose of trial treatment or more frequently if clinically indicated. Imaging timing should follow calendar days and should not be adjusted for delays in cycle starts or extension of MK-3475 cycle frequencies. The same imaging technique should be used in a subject throughout the trial. Local reading (investigator assessment with site radiology reading) will be used to determine eligibility and for subject management. The Sponsor will collect radiological assessments for retrospective analysis by an independent central radiology reviewl vendor. The processes for image collection and transmission to the independent central radiology reviewl vendor are in the Site Imaging Manual.
q. Per the modified RECIST 1.1 used in this protocol, if imaging shows progressive disease, the imaging assessment should be performed at a minimum of 4 weeks later in order to confirm progressive disease as described in Section 4.2.3.1. Please refer to the Procedure Manual for additional details on modifications to RECIST.
r. In subjects who discontinue study therapy without confirmed disease progression, a radiological evaluation should be performed at the time of treatment discontinuation (i.e. date of discontinuation ± 4 week window). If a previous scan was obtained within 4 weeks prior to the date of discontinuation, then a scan at treatment discontinuation isn’t mandatory.
s. Unresolved labs that are drug related AEs should be followed until resolution. Labs do not need to be repeated after the end of treatment if labs are within normal range.
t. Pre-dose trough PK samples will be collected at Cycles 1 and 2. Pre-dose trough samples will be collected every 4 cycles starting with Cycle 5 and through Cycle 37, 30 days after discontinuation of study drug, and 3 months and 6 months after discontinuation of study drug (or until the subject starts new anti-neoplastic therapy). All trough samples should be drawn within 24 hours before infusion of MK-3475. Anti-MK-3475 antibodies should be drawn with all pre-dose trough PK samples, the 30 day discontinuation draw and 3 months and 6 months after discontinuation of study drug (or until the subject starts new anti-neoplastic therapy). Procedures for sample collection are described in the Procedures Manual.
u. Following Cycle 8, the directed physical exam is only required at Cycle 11, 15, 19, and every 4 cycles thereafter.
v Following Cycle 8, the ECOG performance status should be determined only in conjunction with a protocol-specified full or directed physical exam (Cycle 9, 11, 13, 15, 17, 19 and every 2 cycles thereafter.

6.4 Second Course Phase (Retreatment ONLY) for Cohort B2

**Trial Period:**	**Treatment Cycles[a]**						**End of Treatment**	**Post-Treatment**		
				To be repeated beyond 6 cycles						
	Second Course Phase						Discon	Post-Treatment Safety Follow-up	Follow Up Visits[b]	Survival Follow-Up[c]
Treatment Cycle/Title:	1	2	3	4	5	6				
Scheduling Window (Days)[d]:		± 3	± 3	± 3	± 3	± 3	At time of Discon	30 days post discon	Every 8 weeks post discon	Every 12 weeks
**Administrative Procedures**										
Eligibility Criteria[e]	X									
Concomitant Medication Review[f]	X	X	X	X	X	X	X	X		
Trial Treatment Administration[g]	X	X	X	X	X	X				
Post-study anticancer therapy status									X	X
Survival Status										X
**Clinical Procedures/Assessments**										
Review Adverse Events[h]	X	X	X	X	X	X	X	X[i]	X[i]	
Full Physical Examination	X			X[j]						
Directed Physical Examination		X	X		X[t]	X[t]	X			
Vital Signs and Weight[k]	X	X	X	X	X	X	X			
ECOG Performance Status	X	X	X	X	X[u]	X[u]	X			
**Laboratory Procedures/Assessments: analysis performed by local laboratory**										
Pregnancy Test – Urine or Serum β-HCG[l]	X									
PT/INR and aPTT[m]	X[n]									
CBC with Differential[o]	X[n]	X	X	X	X	X	X	X[s]		
Comprehensive Chemistry Panel[o]	X[n]	X	X	X	X	X	X	X[s]		
T3, FT4 and TSH[o]	X[n]			X[j]				X[s]		
**Efficacy Measurements**										
Tumor Imaging[p,q]	X		X				X[r]		X[b]	

a. In general, assessments/procedures are to be performed on Day 1 and prior to the first dose of treatment for each cycle unless otherwise specified. Treatment cycles are 3 weeks Imaging should always be performed every 8 weeks (56 days ± 7 days) regardless of any treatment delays.
b. In subjects who discontinue study therapy without documented disease progression, every effort should be made to continue monitoring their disease status by radiologic imaging[p] until (1) the start of new anti-cancer treatment, (2) documented disease progression, (3) death, or (4) the end of the study, whichever occurs first.
c. After the start of new anti-cancer treatment or documented disease progression, the subject should be contacted by telephone every 12 weeks to assess for survival status.
d. In general, the window for each visit is ± 3 days unless otherwise noted.
e. Subjects who either a) attain a CR and discontinue treatment or b) discontinue treatment after 24 months on MK-3475 for reasons other than disease progression or intolerability may restart trial treatment if they meet the criteria specified in Section 7.1.5.2.1.
f. Concomitant medications – Enter new medications started during the trial through the Safety Follow-up visit. Record all medications taken for SAEs as defined in Section 7.2.
g. Subjects who restart treatment should resume at the same dose and cycle interval which they were receiving prior to discontinuation.
h. AEs and laboratory safety measurements will be graded per NCI CTCAE version 4.0. All AEs, whether gradable by CTCAE or not, will also be evaluated for seriousness.
i. Record all AEs occurring within 30 days after the last dose of trial treatment. Report all SAEs (related and unrelated to trial treatment) and ECIs occurring up until 90 days after the last dose of trial treatment or 30 days after the end of treatment if the subject initiates new anticancer therapy, whichever is earlier. Afterwards, report only SAEs and ECIs that are related to trial treatment.
j. To be repeated every 3 cycles after cycle 4.
k. Vital signs to include temperature, pulse, respiratory rate, weight and blood pressure.
l. For women of reproductive potential, a urine pregnancy test will be performed within 72 hours prior to first dose of retreatment. If urine pregnancy results cannot be confirmed as negative, a serum pregnancy test performed by the local study site laboratory will be required. Pregnancy tests (serum and/or urine tests) should be repeated if required by local guidelines.
m. Coagulation factors (PT/INR and aPTT) should be monitored closely throughout the trial for any subject receiving anticoagulant therapy.
n. Laboratory tests for determining eligibility for retreatment are to be performed within 10 days prior to the first retreatment dose of MK-3475. See Section 7.1.3 for details regarding laboratory tests.
o. After the first dose, lab samples can be collected up to 72 hours prior to the scheduled time point. See Section 7.1.3 for details regarding laboratory tests.
p. A scan must be performed within 28 days prior to restarting treatment with MK-3475. Imaging should continue to be performed every 8 weeks (56 ± 7 days) from the first dose of trial treatment or more frequently if clinically indicated. Imaging timing should follow calendar days and should not be adjusted for delays in cycle starts or extension of MK-3475 cycle frequencies. The same imaging technique should be used in a subject throughout the trial. Local reading (investigator assessment with site radiology reading) will be used to determine eligibility and for subject management. The Sponsor will collect radiological assessments for retrospective analysis by an independent central radiology review vendor. The processes for image collection and transmission to the independent central radiology review vendor are in the Site Imaging Manual.
q. Per the modified RECIST 1.1 used in this protocol, if imaging shows progressive disease, the imaging assessment should be performed at a minimum of 4 weeks later in order to confirm progressive disease as described in Section 4.2.3.1. Please refer to the Procedure Manual for additional details on modifications to RECIST.
r. In subjects who discontinue study therapy without confirmed disease progression, a radiological evaluation should be performed at the time of treatment discontinuation (i.e. date of discontinuation ± 4 week window). If a previous scan was obtained within 4 weeks prior to the date of discontinuation, then a scan at treatment discontinuation isn't mandatory.
s. Unresolved labs that are drug related AEs should be followed until resolution. Labs do not need to be repeated after the end of treatment if labs are within normal range.
t. Following Cycle 6, the directed physical exam is only required clinically appropriate as long as a physical exam is performed every 6 weeks..
u. Following Cycle 6, the ECOG performance status should be determined only in conjunction with a protocol-specified full or directed physical exam.

7.0 TRIAL PROCEDURES

7.1 Trial Procedures

The Trial Flow Chart - Section 6.0 summarizes the trial procedures to be performed at each visit. Individual trial procedures are described in detail below. It may be necessary to perform these procedures at unscheduled time points if deemed clinically necessary by the investigator.

Furthermore, additional evaluations/testing may be deemed necessary by the investigator and or the Sponsor for reasons related to subject safety. In some cases, such evaluation/testing may be potentially sensitive in nature (e.g., HIV, Hepatitis C, etc.), and thus local regulations may require that additional informed consent be obtained from the subject. In these cases, such evaluations/testing will be performed in accordance with those regulations.

7.1.1 Administrative Procedures

7.1.1.1 Informed Consent

The investigator must obtain documented consent from each potential subject prior to participating in a clinical trial or Future Biomedical Research.

7.1.1.1.1 General Informed Consent

Consent must be documented by the subject's dated signature or by the subject's legally acceptable representative's dated signature on a consent form along with the dated signature of the person conducting the consent discussion.

A copy of the signed and dated consent form should be given to the subject before participation in the trial.

The initial informed consent form, any subsequent revised written informed consent form and any written information provided to the subject must receive the IRB/ERC's approval/favorable opinion in advance of use. The subject or his/her legally acceptable representative should be informed in a timely manner if new information becomes available that may be relevant to the subject's willingness to continue participation in the trial. The communication of this information will be provided and documented via a revised consent form or addendum to the original consent form that captures the subject's dated signature or by the subject's legally acceptable representative's dated signature.

Specifics about a trial and the trial population will be added to the consent form template at the protocol level.

The informed consent will adhere to IRB/ERC requirements, applicable laws and regulations and Sponsor requirements.

7.1.1.1.2 Consent and Collection of Specimens for Future Biomedical Research

The investigator or qualified designee will explain the Future Biomedical Research consent to the subject, answer all of his/her questions, and obtain written informed consent before performing any procedure related to the Future Biomedical Research sub-trial. A copy of the informed consent will be given to the subject.

7.1.1.2 Inclusion/Exclusion Criteria

All inclusion and exclusion criteria will be reviewed by the investigator or qualified designee to ensure that the subject qualifies for the trial.

7.1.1.3 Subject Identification Card

All subjects will be given a Subject Identification Card identifying them as participants in a research trial. The card will contain trial site contact information (including direct telephone numbers) to be utilized in the event of an emergency. The investigator or qualified designee will provide the subject with a Subject Identification Card after the subject provides written informed consent.

7.1.1.4 Medical History

A medical history will be obtained by the investigator or qualified designee. Medical history will include all active conditions, and any condition diagnosed within the prior 10 years that are considered to be clinically significant by the Investigator. Details regarding the disease for which the subject has enrolled in this study will be recorded separately and not listed as medical history.

7.1.1.5 Prior and Concomitant Medications Review

7.1.1.5.1 Prior Medications

The investigator or qualified designee will review prior medication use, including any protocol-specified washout requirement, and record prior medication taken by the subject within 28 days before starting the trial. Treatment for the disease for which the subject has enrolled in the study will be recorded separately and not listed as a prior medication.

7.1.1.5.2 Concomitant Medications

The investigator or qualified designee will record medication, if any, taken by the subject during the trial. All medications related to reportable SAEs and ECIs should be recorded as defined in Section 7.2.

7.1.1.6 Disease Details and Treatments

7.1.1.6.1 Disease Details

The investigator or qualified designee will obtain prior and current details regarding disease status. HPV status will be collected for those subjects enrolled in Cohort B and B2.

7.1.1.6.2 Prior Treatment Details

The investigator or qualified designee will review all prior cancer treatments including systemic treatments, radiation and surgeries.

7.1.1.6.3 Subsequent Anti-neoplastic Therapy Status

The investigator or qualified designee will review all new anti-neoplastic therapy initiated after the last dose of trial treatment. If a subject initiates a new anti-neoplastic therapy within 30 days after the last dose of trial treatment, the 30 day Safety Follow-up visit must occur before the first dose of the new therapy. Once new anti-neoplastic therapy has been initiated the subject will move into survival follow-up.

7.1.1.7 Assignment of Screening Number

All consented subjects will be given a unique screening number that will be used to identify the subject for all procedures that occur prior to randomization or treatment allocation. Each subject will be assigned only one screening number. Screening numbers must not be re-used for different subjects. Any subject who is screened multiple times will retain the original screening number assigned at the initial screening visit.

7.1.1.8 Assignment of Randomization Number

All eligible subjects will be allocated, by non-random assignment, to trial treatment and will receive a unique number. This unique number is termed a randomization number throughout the protocol for operational purposes. The randomization number identifies the subject for all procedures occurring after treatment allocation. Once a randomization number is assigned to a subject, it can never be re-assigned to another subject.

A single subject cannot be assigned more than 1 randomization number.

7.1.1.9 Trial Compliance (Medication/Diet/Activity/Other)

Interruptions from the protocol specified treatment plan for greater than 12 weeks between MK-3475 doses due to toxicity require consultation between the investigator and the Sponsor and written documentation of the collaborative decision on subject management.

Administration of trial medication will be witnessed by the investigator and/or trial staff. The total volume of trial treatment infused will be compared to the total volume prepared to determine compliance with each dose administered.

The instructions for preparing and administering MK-3475 will be provided in the Pharmacy Manual.

7.1.2 Clinical Procedures/Assessments

7.1.2.1 Adverse Event (AE) Monitoring

The investigator or qualified designee will assess each subject to evaluate for potential new or worsening AEs as specified in the Trial Flow Chart and more frequently if clinically indicated. Adverse experiences will be graded and recorded throughout the study and during the follow-up period according to NCI CTCAE Version 4.0 (see Section 12.5). Toxicities will be characterized in terms regarding seriousness, causality, toxicity grading, and action taken with regard to trial treatment.

All AEs of unknown etiology associated with MK-3475 exposure should be evaluated to determine if it is possibly an event of clinical interest (ECI) of a potentially immunologic etiology (irAE). See Section 5.6.1.1 and the separate guidance document in the administrative binder regarding the identification, evaluation and management of AEs of a potential immunological etiology.

Please refer to section 7.2 for detailed information regarding the assessment and recording of AEs.

7.1.2.2 Physical Exam

7.1.2.2.1 Full Physical Exam

The investigator or qualified designee will perform a complete physical exam during the screening period. Clinically significant abnormal findings should be recorded as medical history. A full physical exam should be performed as specified in the Trial Flow Chart (Section 6.0) for the appropriate cohort. After the first dose of trial treatment new clinically significant abnormal findings should be recorded as AEs.

7.1.2.2.2 Directed Physical Exam

For cycles that do not require a full physical exam per the Trial Flow Chart, the investigator or qualified designee will perform a directed physical exam as clinically indicated prior to trial treatment administration. Directed physical exams should be performed as specified in the Trial Flow Chart (Section 6.0) for the appropriate cohort. For Cohorts A, B, C and D after Cycle 8 directed physical exams should occur at Cycle 11 and every 4 cycles thereafter. For Cohort B2 after Cycle 6 a physical exam (either full or directed) is required to be performed at least every 6 weeks but should be performed more frequently if clinically indicated. New clinically significant abnormal findings should be recorded as AEs.

7.1.2.3 Vital Signs

The investigator or qualified designee will take vital signs at screening, prior to the administration of each dose of trial treatment and at treatment discontinuation as specified in the Trial Flow Chart (Section 6.0). Vital signs should include temperature, pulse, respiratory rate, weight and blood pressure. Height will be measured at screening only.

7.1.2.4 Eastern Cooperative Oncology Group (ECOG) Performance Scale

The investigator or qualified designee will assess ECOG status (see Section 12.4) at screening, prior to the administration of each dose of trial treatment and discontinuation of trial treatment as specified in the Trial Flow Chart. After Cycle 8 (Cohorts A, B, C or D) or Cycle 6 (Cohort B2) assessment of ECOG status will be performed in conjunction with the directed or full physical exam.

7.1.2.5 Tumor Imaging and Assessment of Disease

Processes for image collection and transmission to the independent central radiology review vendor can be found in the Site Imaging Manual.

7.1.2.5.1 Assessment of Disease

RECIST 1.1 will be applied by the site as the primary measure for assessment of tumor response and as a basis for all protocol guidelines related to disease status (e.g., discontinuation of study therapy).

RECIST 1.1 will be adapted as follows to account for the unique tumor response seen in this class of therapeutics.

If imaging shows PD, tumor assessment should be repeated ≥ 4 weeks later in order to confirm PD with the option of continuing treatment for clinically stable subjects as discussed below in Table 6. Clinically stable is defined by the following criteria:

- Absence of signs and symptoms indicating disease progression
- No decline in ECOG performance status
- Absence of rapid progression of disease
- Absence of progressive tumor at critical anatomical sites (e.g., cord compression) requiring urgent alternative medical intervention

Table 6 Imaging and Treatment after 1st radiologic evidence of PD

	Clinically Stable		Clinically Unstable	
	**Imaging**	**Treatment**	**Imaging**	**Treatment**
1st radiologic evidence of PD	Repeat imaging at ≥ 4 weeks to confirm PD	May continue study treatment at the Investigator's discretion while awaiting confirmatory scan	Repeat imaging at ≥ 4 weeks to confirm PD if possible	Discontinue treatment
Repeat scan confirms PD	No additional imaging required	Discontinue treatment (exception noted below)	No additional imaging required	N/A
Repeat scan shows SD, PR or CR	Continue regularly scheduled imaging assessments every 8 weeks	Continue study treatment at the Investigator's discretion	Continue regularly scheduled imaging assessments every 8 weeks	May restart study treatment if condition has improved and/or clinically stable per Investigator's discretion

In determining whether or not the tumor burden has increased or decreased, Investigators should consider all target lesions as well as non-target lesions (please refer to the Site Imaging Manual). Subjects that are deemed clinically unstable are not required to have repeat imaging for confirmation. If radiologic progression is confirmed, then the subject will be discontinued from trial treatment. If radiologic progression is not confirmed, then the subject should resume/continue trial treatment and have their next scan according to the every 8 week (56 ± 7 days) schedule.

NOTE: If a subject with confirmed radiographic progression (i.e. 2 scans at least 28 days apart demonstrating progressive disease) is clinically stable or clinically improved, and there is no further increase in the tumor dimensions at the confirmatory scan, an exception may be considered to continue treatment upon consultation with the Sponsor. Clinically stable subjects should also have at the confirmatory scan no further increase in the target lesions, no unequivocal increase in non-target lesions, and no additional new lesions develop (non-worsening PD) to continue study treatment.

Imaging during the follow-up period is to be repeated every 8 weeks (56 ± 7 days) for subjects who discontinue trial treatment for reasons other than disease progression until the subject experiences confirmed disease progression or starts a new anti-neoplastic therapy.

Local reading (investigator assessment with site radiology reading) based on RECIST 1.1 will be used to determine subject eligibility and for subject management. The Sponsor will also receive radiologic images for a retrospective analysis of subject eligibility and treatment response to be performed by an independent central radiology reviewl vendor, including RECIST 1.1 and evaluation of volumetric tumor response.

7.1.2.5.2 Initial Tumor Imaging

Initial tumor imaging must be performed within 28 days prior to the first dose of trial treatment. The site study team must review pre-trial images to confirm the subject has measurable disease per RECIST 1.1. The baseline imaging scan should be submitted to the independent central radiology review vendor for a retrospective analysis of this eligibility criterion.

Scans performed as part of routine clinical management are acceptable for use as the screening scan if they are of diagnostic quality and performed within 28 days prior to the first dose of trial treatment. The same imaging technique should be used in a subject throughout the study.

7.1.2.5.3 Tumor Imaging During Trial

Tumor imaging may be performed by CT or magnetic resonance imaging (MRI), but the same imaging technique should be used in a subject throughout the trial. Imaging should be performed every 8 weeks (56 days ± 7 days) from the first dose of trial treatment or more frequently if clinically indicated. Imaging should not be delayed for delays in cycle starts or extension of MK-3475 cycle intervals.

Per RECIST 1.1, response should be confirmed by a repeat radiographic assessment not less than 4 weeks from the date the response was first documented. The scan for confirmation of response may be performed at the earliest 4 weeks after the first indication of response, or at the next scheduled scan (8 weeks later), whichever is clinically indicated.

Imaging should continue to be performed until documented disease progression, the start of new anti-cancer treatment, withdrawal of consent, death, or the end of the study, whichever occurs first. Disease progression should be confirmed at least 4 weeks after the first scan indicating progressive disease in clinically stable subjects. Subjects who have unconfirmed disease progression may continue on treatment until progression is confirmed provided they have met the conditions detailed in Section 7.1.2.5.1.

7.1.2.6 Tumor Tissue Collection and Correlative Studies Blood Sampling

Enrollment in this study is limited to those subjects with tumors who have submitted archival FFPE tumor sample or newly obtained core or excisional biopsy (FNA not adequate) to a central lab for PD-L1 characterization.. These samples are not required to be obtained within 28 days of enrollment, however, a biopsy for screening purposes cannot be performed until the main consent is signed.

Enrollment in Cohorts A, B, C and D of this study is limited to those subjects with tumors characterized as PD-L1 positive by IHC at a central laboratory.

Subjects in Cohort B2 should submit both archival FFPE tumor samples and newly obtained biopsy samples (as described below) to the central laboratory. PD-L1 expression by IHC will be evaluated retrospectively. The tumor biopsy specimen must be sufficient for assessment of PD-L1 expression by the central lab or an additional sample will be required. Exceptions to this requirement must be discussed with the Sponsor prior to enrollment in the study.

Biopsy sites should be selected so that subsequent biopsies can be performed at the same location. Exceptions from the mandatory tumor biopsy requirement that allow subjects to continue receiving trial treatment must occur in consultation with the Sponsor.

Blood for correlative biomarker studies should be collected at baseline, on treatment and upon treatment discontinuation. The treatment sample should be collected at Cycle 5 (subjects enrolled in Cohorts A, B, C and D) or Cycle 3 (subjects enrolled in Cohort B2) as appropriate.

Serial tumor biopsy requirements for each cohort are detailed below:

<u>Cohort A – TNBC</u>

Every effort should be made to obtain an optional biopsy prior to Cycle 1 initiation of MK-3475, at Cycle 5, and upon disease progression. If clinical activity of MK-3475 is observed (2 responders in Cohort A), the pre-dose Cycle 1 biopsy and the biopsy at Cycle 5 will become mandatory for all remaining subjects enrolled into the cohort. A tumor biopsy at treatment discontinuation is highly encouraged for all subjects. Tumor biopsies that are inaccessible or contraindicated due to subject safety concerns are exempt from this requirement.

<u>Cohort B – H/N Cancer</u>

Tumor biopsies are mandatory for subjects prior to Cycle 1 initiation of MK-3475 and again at Cycle 5. A tumor biopsy at treatment discontinuation is highly encouraged. Tumor biopsies that are inaccessible or contraindicated due to subject safety concerns are exempt from this requirement.

<u>Cohort B2 – H/N Cancer</u>

Newly obtained tumor biopsies are mandatory for subjects prior to Cycle 1 initiation of MK-3475 and again at Cycle 3. Exemptions to the mandatory tumor biopsy require Sponsor approval and appropriate justification. A tumor biopsy at the time of treatment discontinuation is highly encouraged.

Cohort C – Urothelial Tract Cancer

Every effort should be made to obtain an optional biopsy prior to Cycle 1 initiation of MK-3475, at Cycle 5, and upon disease progression. If clinical activity of MK-3475 is observed (2 responders in Cohort C), the pre-dose Cycle 1 biopsy and the biopsy at Cycle 5 will become mandatory for all remaining subjects enrolled into the cohort. A tumor biopsy at treatment discontinuation is highly encouraged for all subjects. Tumor biopsies that are inaccessible or contraindicated due to subject safety concerns are exempt from this requirement.

Cohort D – Gastric Cancer

Tumor biopsies are mandatory for subjects prior to Cycle 1 initiation of MK-3475 and again at Cycle 5. A tumor biopsy at treatment discontinuation is highly encouraged. Tumor biopsies that are inaccessible or contraindicated due to subject safety concerns are exempt from this requirement.

Detailed instructions for tissue collection, process and shipment are provided in the Procedures Manual.

7.1.3 Laboratory Procedures/Assessments

Details regarding specific laboratory procedures/assessments to be performed in this trial are provided below. The total amount of blood/tissue to be drawn/collected over the course of the trial (from pre-trial to post-trial visits), including approximate blood/tissue volumes drawn/collected by visit and by sample type per subject can be found in the Procedures Manual.

7.1.3.1 Laboratory Safety Evaluations (Hematology, Chemistry and Urinalysis)

Laboratory tests for hematology, chemistry, urinalysis, and others are specified in Table 7.

Table 7 Laboratory Tests

Hematology‡	Chemistry	Urinalysis	Other
Hematocrit	Albumin	Blood	Serum β-human chorionic gonadotropin (β-hCG)†
Hemoglobin	Alkaline phosphatase	Glucose	PT (INR)
Platelet count	Alanine aminotransferase (ALT)	Protein	aPTT
WBC (total and differential)	Aspartate aminotransferase (AST)	Specific gravity	Total thriiodothyronine (T3)¶
Red Blood Cell Count	Lactate dehydrogenase (LDH)	Microscopic exam, if abnormal results are noted‖	Free tyroxine (T4)
Absolute Neutrophil Count	Carbon Dioxide§ (CO_2 or biocarbonate)	Urine pregnancy test*	Thyroid stimulating hormone (TSH)
Absolute Lymphocyte Count	Creatinine††		Anti-MK-3475 Antibodies
	Uric Acid		PK
	Calcium		Blood for FBR
	Chloride		Blood for correlative studies
	Glucose		
	Phosphorus		
	Potassium		
	Sodium		
	Magnesium		
	Total Bilirubin		
	Direct Bilirubin, if total bilirubin is elevated above the upper limit of normal		
	Total protein		
	Blood Urea Nitrogen		

† Perform on women of childbearing potential only. If urine pregnancy results cannot be confirmed as negative, a serum pregnancy test will be required.
‡ Either differential or absolute value is acceptable
§ If considered standard of care in your region
‖ Institutional standards are acceptable
¶ Free T3 may be performed in place of Total T3 per local standards
†† GFR (measured or calculated) or CrCl can be used in place of creatinine

Laboratory tests for screening or entry into the Second Course Phase should be performed within 10 days prior to the first dose of treatment. After Cycle 1, pre-dose laboratory procedures can be conducted up to 72 hours prior to dosing. Results must be reviewed by the investigator or qualified designee and found to be acceptable prior to each dose of trial treatment.

7.1.3.2 Pharmacokinetic/Pharmacodynamic Evaluations

7.1.3.2.1 Blood Collection for Serum MK-3475

Sample collection, storage and shipment instructions for serum samples will be provided in the Procedures Manual.

The timepoints for PK blood sampling are described in Section 6 – Trial Flow Chart.

Please note that PK blood sampling will occur in Cohorts A, B, C, and D only. Subjects in Cohort B2 (H/N expansion) will not undergo PK blood sampling.

7.1.3.2.2 Blood Collection for Anti-MK-3475 Antibodies

Sample collection, storage and shipment instructions for blood samples will be provided in the Procedures Manual.

Please note that blood collection for anti-MK-3475 antibodies will occur in Cohorts A, B, C, and D only. Subjects in Cohort B2 (H/N expansion) will not undergo blood collection for anti-MK-3475 antibodies.

7.1.3.3 Future Biomedical Research

The following specimens are to be obtained as part of Future Biomedical Research:

- Blood for genomics use
- Leftover archival tumor tissue or leftover newly obtained biopsy samples taken throughout the study

7.1.4 Other Procedures

7.1.4.1 Withdrawal/Discontinuation

When a subject discontinues/withdraws prior to trial completion, all applicable activities scheduled for the final trial visit should be performed at the time of discontinuation. Any adverse events which are present at the time of discontinuation/withdrawal should be followed in accordance with the safety requirements outlined in Section 7.2 - Assessing and Recording Adverse Events. Subjects who a) attain a CR or b) complete 24 months of treatment with MK-3475 may discontinue treatment with the option of restarting treatment if they meet the criteria specified in Section 7.1.5.2.1. After discontinuing treatment following assessment of CR, these subjects should return to the site for a Safety Follow-up Visit (described in Section 7.1.5.3.1) and then proceed to the Follow-Up Period of the study (described in Section 7.1.5.4).

7.1.4.1.1 Withdrawal From Future Biomedical Research

Subjects may withdraw their consent for Future Biomedical Research and have their specimens and all derivatives destroyed. Subjects may withdraw consent at any time by writing to the principal investigator for the main trial. If medical records for the main trial are still available, the investigator will contact the Sponsor using the designated mailbox (clinical.specimen.management@merck.com), and a form will be provided by the Sponsor to obtain appropriate information to complete specimen withdrawal. Subsequently, the subject's specimens will be removed from the biorepository and be destroyed. A letter will be sent from the Sponsor to the investigator confirming the destruction. It is the responsibility of the investigator to inform the subject of completion of destruction. Any analyses in progress at the time of request for destruction or already performed prior to the request being received by the Sponsor will continue to be used as part of the overall research trial data and results. No new analyses would be generated after the request is received.

In the event that the medical records for the main trial are no longer available (e.g., if the investigator is no longer required by regulatory authorities to retain the main trial records) or the specimens have been completely anonymized, there will no longer be a link between the subject's personal information and their specimens. In this situation, the request for specimen destruction cannot be processed.

7.1.4.2 Blinding/Unblinding

This is an open label trial; there is no blinding for this trial.

7.1.5 Visit Requirements

Visit requirements are outlined in Section 6.0 - Trial Flow Chart. Specific procedure-related details are provided above in Section 7.1 - Trial Procedures.

7.1.5.1 Screening

7.1.5.1.1 Pre-screening Period

The Pre-screening period may be utilized by subjects in Cohorts A, B, C and D to determine biomarker eligibility based on PD-L1 status using an archival tumor biopsy sample. After providing a pre-screening consent, subjects will be assigned a screening number. Characterization of PD-L1 status will be performed at a pre-screening visit for subjects with an available archival tumor biopsy sample.

Subjects that do not have an archival tumor biopsy sample available must provide written consent for the main study before the tumor biopsy or any other protocol-specified procedures can occur. These subjects will not enter the pre-screening period as eligibility based on PD-L1 expression will be determined in the main study screening period.

As PD-L1 tumor expression is not an inclusion criterion for subjects in Cohort B2, the Pre-screening period is not relevant for this cohort and all subjects in this cohort will begin the study in the Screening Period.

7.1.5.1.2 Screening Period

Approximately 28 days prior to enrollment, potential subjects will be evaluated to determine that they fulfill the entry requirements as set forth in Section 5.1. Visit requirements are outlined in Section 6.0 – Trial Flow Chart.

Written consent for the main study must be obtained prior to performing any protocol specific procedure. Results of a test performed prior to the subject signing consent as part of routine clinical management are acceptable in lieu of a screening test if performed within the specified time frame. Screening procedures are to be completed within 28 days prior to the first dose of trial treatment except for the following:

- Laboratory tests are to be performed within 10 days prior to the first dose of trial treatment
- For women of reproductive potential, a urine pregnancy test will be performed within 72 hours prior to the first dose of trial treatment. If urine pregnancy results cannot be confirmed as negative, a serum pregnancy test will be required (performed by the local study site laboratory)
- Biopsy for PD-L1 characterization is not required to be obtained within 28 days prior to the first dose of trial treatment

Subjects may be rescreened after initially failing to meet the inclusion/exclusion criteria. Results from assessments performed during the initial screening period are acceptable in lieu of a repeat screening test if performed within the specified time frame and the inclusion/exclusion criteria is met.

7.1.5.2 Treatment Period

7.1.5.2.1 Second Course Phase (Retreatment Period)

Subjects who stop MK-3475 with SD or better may be eligible for up to one year of additional MK-3475 therapy if they progress after stopping MK-3745. This retreatment is termed the Second Course Phase of this study and is only available if the study remains open and the subject meets the following conditions:

- Either
    - Stopped initial treatment with MK-3475 after attaining an investigator-determined confirmed CR according to RECIST 1.1

- Was treated for at least 24 weeks with MK-3475 before discontinuing therapy
- Received at least two treatments with MK-3475 beyond the date when the initial CR was declared

OR

- Subject had SD, PR or CR and stopped MK-3475 treatment after 24 months of study therapy for reasons other than disease progression or intolerability

AND

- Experienced an investigator-determined confirmed radiographic disease progression after stopping their initial treatment with MK-3475
- Did not receive any anti-cancer treatment since the last dose of MK-3475
- Have a performance status of 0 or 1 on the ECOG Performance Scale
- Demonstrate adequate organ function as detailed in Section 5.1.2
- Female subject of childbearing potential should have a negative urine or serum pregnancy test within 72 hours prior to receiving retreatment with study medication.
- Female subjects of childbearing potential should be willing to use 2 methods of birth control or be surgically sterile, or abstain from heterosexual activity for the course of the study through 120 days after the last dose of study medication (Reference Section 5.7.2). Subjects of child bearing potential are those who have not been surgically sterilized or have been free from menses for > 1 year.
- Male subjects should agree to use an adequate method of contraception starting with the first dose of study therapy through 120 days after the last dose of study therapy.
- Does not have a history or current evidence of any condition, therapy, or laboratory abnormality that might interfere with the subject's participation for the full duration of the trial or is not in the best interest of the subject to participate, in the opinion of the treating investigator.

Subjects who restart treatment will be retreated at the same dose frequency as when they last received MK-3475. Treatment will be administered for up to one additional year.

Visit requirements are outlined in Section 6.0 – Trial Flow Chart.

7.1.5.3 Post-Treatment Visits

7.1.5.3.1 Safety Follow-Up Visit

The mandatory Safety Follow-Up Visit should be conducted approximately 30 days after the last dose of trial treatment or before the initiation of a new anti-neoplastic treatment, whichever comes first. All AEs that occur prior to the Safety Follow-Up Visit should be recorded. Subjects with an AE of Grade > 1 will be followed until the resolution of the AE to Grade 0-1 or until the beginning of a new anti-neoplastic therapy, whichever occurs first. SAEs that occur within 90 days of the end of treatment or 30 days after the end of treatment if the subject initiates new anticancer therapy should also be followed and recorded.

Subjects who are eligible for retreatment with MK-3475 (as described in Section 7.1.5.2.1) may have up to two safety follow-up visits, one after the Treatment Period and one after the Second Course Phase.

7.1.5.4 Follow-up Visits

Subjects who discontinue trial treatment for a reason other than disease progression will move into the Follow-Up Phase and should be assessed every 8 weeks (56 ± 7 days) by radiologic imaging to monitor disease status. Every effort should be made to collect information regarding disease status until the start of new anti-neoplastic therapy, disease progression, death, end of the study or if the subject begins retreatment with MK-3475 as detailed in Section 7.1.5.2.1. Information regarding post-study anti-neoplastic treatment will be collected if new treatment is initiated.

Subjects who are eligible to receive retreatment with MK-3475 according to the criteria in Section 7.1.5.2.1 will move from the follow-up phase to the Second Course Phase when they experience disease progression. Details are provided in Section 6.2 – Trial Flow Chart for Retreatment.

7.1.5.4.1 Survival Follow-up

Once a subject experiences confirmed disease progression or starts a new anti-neoplastic therapy, the subject moves into the survival follow-up phase and should be contacted by telephone every 12 weeks (+/- 4 weeks) to assess for survival status until death, withdrawal of consent, or the end of the study, whichever occurs first.

7.2 Assessing and Recording Adverse Events

An adverse event is defined as any untoward medical occurrence in a patient or clinical investigation subject administered a pharmaceutical product and which does not necessarily have to have a causal relationship with this treatment. An adverse event can therefore be any unfavourable and unintended sign (including an abnormal laboratory finding, for example), symptom, or disease temporally associated with the use of a medicinal product or protocol-specified procedure, whether or not considered related to the medicinal product or protocol-specified procedure. Any worsening (i.e., any clinically significant adverse change in frequency and/or intensity) of a preexisting condition that is temporally associated with the use of the Sponsor's product, is also an adverse event.

Changes resulting from normal growth and development that do not vary significantly in frequency or severity from expected levels are not to be considered adverse events. Examples of this may include, but are not limited to, teething, typical crying in infants and children and onset of menses or menopause occurring at a physiologically appropriate time.

Sponsor's product includes any pharmaceutical product, biological product, device, diagnostic agent or protocol-specified procedure, whether investigational (including placebo or active comparator medication) or marketed, manufactured by, licensed by, provided by or distributed by the Sponsor for human use.

Adverse events may occur during clinical trials, or as prescribed in clinical practice, from overdose (whether accidental or intentional), from abuse and from withdrawal.

Progression of the cancer under study is not considered an adverse event.

All adverse events that occur after the consent form is signed but before randomization/treatment allocation must be reported by the investigator if they cause the subject to be excluded from the trial, or are the result of a protocol-specified intervention, including but not limited to washout or discontinuation of usual therapy, diet, placebo treatment or a procedure.

From the time of randomization/treatment allocation through 30 days following cessation of treatment, all adverse events must be reported by the investigator. Such events will be recorded at each examination on the Adverse Event case report forms/worksheets. The reporting timeframe for adverse events meeting any serious criteria is described in section 7.2.3.1. The investigator will make every attempt to follow all subjects with non-serious adverse events for outcome.

Electronic reporting procedures can be found in the EDC data entry guidelines. Paper reporting procedures can be found in the Investigator Trial File Binder (or equivalent).

Adverse events will not be collected for subjects during the pre-screening period (for determination of archival tissue status) as long as that subject has not undergone any protocol-specified procedure or intervention. If the subject requires a blood draw, fresh tumor biopsy etc., the subject is first required to provide consent to the main study and AEs will be captured according to guidelines for standard AE reporting.

7.2.1 Definition of an Overdose for This Protocol and Reporting of Overdose to the Sponsor

For purposes of this trial, an overdose will be defined as any dose exceeding the prescribed dose for MK-3475 by 20% over the prescribed dose. No specific information is available on the treatment of overdose of MK-3475. In the event of overdose, MK-3475 should be discontinued and the subject should be observed closely for signs of toxicity. Appropriate supportive treatment should be provided if clinically indicated.

If an adverse event(s) is associated with ("results from") the overdose of Sponsor's product or vaccine, the adverse event(s) is reported as a serious adverse event, even if no other seriousness criteria are met.

If a dose of Sponsor's product or vaccine meeting the protocol definition of overdose is taken without any associated clinical symptoms or abnormal laboratory results, the overdose is reported as a non-serious Event of Clinical Interest (ECI), using the terminology "accidental or intentional overdose without adverse effect."

All reports of overdose with and without an adverse event must be reported within 24 hours to the Sponsor either by electronic media or paper. Sponsor Contact information can be found in the Investigator Trial File Binder.

7.2.2 Reporting of Pregnancy and Lactation to the Sponsor

Although pregnancy and lactation are not considered adverse events, it is the responsibility of investigators or their designees to report any pregnancy or lactation in a subject (spontaneously reported to them) that occurs during the trial.

Pregnancies and lactations that occur after the consent form is signed but before randomization/treatment allocation must be reported by the investigator if they cause the subject to be excluded from the trial, or are the result of a protocol-specified intervention, including but not limited to washout or discontinuation of usual therapy, diet, placebo treatment or a procedure.

Pregnancies and lactations that occur from the time of randomization/treatment allocation through 120 days following cessation of Sponsor's product, or 30 days following cessation of treatment if the subject initiates new anticancer therapy, whichever is earlier, must be reported by the investigator. All reported pregnancies must be followed to the completion/termination of the pregnancy. Pregnancy outcomes of spontaneous abortion, missed abortion, benign hydatidiform mole, blighted ovum, fetal death, intrauterine death, miscarriage and stillbirth must be reported as serious events (Important Medical Events). If the pregnancy continues to term, the outcome (health of infant) must also be reported.

Such events must be reported within 24 hours to the Sponsor either by electronic media or paper. Electronic reporting procedures can be found in the EDC data entry guidelines. Paper reporting procedures can be found in the Investigator Trial File Binder (or equivalent).

7.2.3 Immediate Reporting of Adverse Events to the Sponsor

7.2.3.1 Serious Adverse Events

A serious adverse event is any adverse event occurring at any dose or during any use of Sponsor's product that:

- Results in death;
- Is life threatening;
- Results in persistent or significant disability/incapacity;
- Results in or prolongs an existing inpatient hospitalization;
- Is a congenital anomaly/birth defect;
- Is an other important medical event

Note: In addition to the above criteria, adverse events meeting either of the below criteria, although not serious per ICH definition, are reportable to the Sponsor in the same timeframe as SAEs to meet certain local requirements. Therefore, these events are considered serious by the Sponsor for collection purposes.

- Is a new cancer (that is not a condition of the study);
- Is associated with an overdose.

Refer to Table 8 for additional details regarding each of the above criteria.

For the time period beginning when the consent form is signed until randomization/treatment allocation, any serious adverse event, or follow up to a serious adverse event, including death due to any cause other than progression of the cancer under study (reference Section 7.2.3.3 for additional details), that occurs to any subject must be reported within 24 hours to the Sponsor if it causes the subject to be excluded from the trial, or is the result of a protocol-specified intervention, including but not limited to washout or discontinuation of usual therapy, diet, placebo treatment or a procedure.

For the time period beginning at randomization/treatment allocation through 90 days following cessation of treatment, or 30 days following cessation of treatment if the subject initiates new anticancer therapy, whichever is earlier, any serious adverse event, or follow up to a serious adverse event, including death due to any cause other than progression of the cancer under study (reference Section 7.2.3.3 for additional details), whether or not related to the Sponsor's product, must be reported within 24 hours to the Sponsor either by electronic media or paper. Electronic reporting procedures can be found in the EDC data entry guidelines. Paper reporting procedures can be found in the Investigator Trial File Binder (or equivalent)

Additionally, any serious adverse event, considered by an investigator who is a qualified physician to be related to the Sponsor's product that is brought to the attention of the investigator at any time outside of the time period specified in the previous paragraph also must be reported immediately to the Sponsor.

All subjects with serious adverse events must be followed up for outcome.

7.2.3.2 Events of Clinical Interest

Selected non-serious and serious adverse events are also known as Events of Clinical Interest (ECI) and must be reported to the Sponsor.

For the time period beginning when the consent form is signed until randomization/treatment allocation, any ECI, or follow up to an ECI, that occurs to any subject must be reported within 24 hours to the Sponsor if it causes the subject to be excluded from the trial, or is the result of a protocol-specified intervention, including but not limited to washout or discontinuation of usual therapy, diet, placebo treatment or a procedure.

For the time period beginning at randomization/treatment allocation through 90 days following cessation of treatment, or 30 days following cessation of treatment if the subject initiates new anticancer therapy, whichever is earlier, any ECI, or follow up to an ECI, whether or not related to the Sponsor's product, must be reported within 24 hours to the Sponsor, either by electronic media or paper. Electronic reporting procedures can be found in the EDC data entry guidelines. Paper reporting procedures can be found in the Investigator Trial File Binder (or equivalent).

Events of clinical interest for this trial include:

1. an overdose of Sponsor's product, as defined in Section 7.2.1 - Definition of an Overdose for This Protocol and Reporting of Overdose to the Sponsor, that is not associated with clinical symptoms or abnormal laboratory results.

2. an elevated AST or ALT lab value that is greater than or equal to 3X the upper limit of normal and an elevated total bilirubin lab value that is greater than or equal to 2X the upper limit of normal and, at the same time, an alkaline phosphatase lab value that is less than 2X the upper limit of normal, as determined by way of protocol-specified laboratory testing or unscheduled laboratory testing.*

   *Note: These criteria are based upon available regulatory guidance documents. The purpose of the criteria is to specify a threshold of abnormal hepatic tests that may require an additional evaluation for an underlying etiology. The trial site guidance for assessment and follow up of these criteria can be found in the Investigator Trial File Binder.

3. Additional adverse events:

A separate guidance document has been provided entitled Event of Clinical Interest Guidance Document" (previously entitled "Event of Clinical Interest and Immune-Related Adverse Event Guidance Document"). This document can be found in the administrative binder and provides guidance regarding identification, evaluation and management of ECIs and irAEs.

Subjects should be assessed for possible ECIs prior to each dose. Lab results should be evaluated and subjects should be asked for signs and symptoms suggestive of an immune-related event. Subjects who develop an ECI thought to be immune-related should have additional testing to rule out other etiologic causes. If lab results or symptoms indicate a possible immune-related ECI, then additional testing should be performed to rule out other etiologic causes. If no other cause is found, then it is assumed to be immune-related.

ECIs that occur to any subject from the date of first dose through 90 days following cessation of treatment, or 30 days after the end of treatment if the subject initiates new anticancer therapy, whichever is earlier, whether or not related to the Sponsor's product, must be reported within 24 hours to the Sponsor either by electronic media or paper. Sponsor Contact information can be found in the administrative binder.

7.2.3.3 Protocol-Specific Exceptions to Serious Event Reporting

Efficacy endpoints as outlined in this section will not be reported to the Sponsor as described in Section 7.2.3. - Immediate Reporting of Adverse Events to the Sponsor.

Specifically, the suspected/actual events covered in this exception include any event that is disease progression of the cancer under study.

The Sponsor will monitor unblinded aggregated efficacy endpoint events and safety data to ensure the safety of the subjects in the trial. Any suspected endpoint which upon review is not progression of the cancer under study will be forwarded to global safety as a SAE within 24 hours of determination that the event is not progression of the cancer under study.

7.2.4 Evaluating Adverse Events

An investigator who is a qualified physician will evaluate all adverse events according to the NCI Common Terminology for Adverse Events (CTCAE), version 4.0. Any adverse event which changes CTCAE grade over the course of a given episode will have each change of grade recorded on the adverse event case report forms/worksheets.

All adverse events regardless of CTCAE grade must also be evaluated for seriousness.

Table 8 Evaluating Adverse Events

An investigator who is a qualified physician, will evaluate all adverse events as to:

**V4.0 CTCAE Grading**	**Grade 1**	**Mild; asymptomatic or mid symptoms; clinical or diagnostic observations only; intervention not indicated.**
	**Grade 2**	**Moderate; minimal, local or noninvasive intervention indicated; limiting age-appropriate instrumental ADL.**
	**Grade 3**	**Severe or medically significant but not immediately life-threatening; hospitalization or prolongation or hospitalization indicated; disabling; limiting self-care ADL.**
	**Grade 4**	**Life threatening consequences; urgent intervention indicated.**
	**Grade 5**	**Death related to AE**
**Seriousness**	A serious adverse event is any adverse event occurring at any dose or during any use of Sponsor's product that:	
	†**Results in death**; or	
	†**Is life threatening; or** places the subject, in the view of the investigator, at immediate risk of death from the event as it occurred (Note: This does not include an adverse event that, had it occurred in a more severe form, might have caused death.); or	
	†**Results in a persistent or significant disability/incapacity** (substantial disruption of one's ability to conduct normal life functions); or	
	†**Results in or prolongs an existing inpatient hospitalization** (hospitalization is defined as an inpatient admission, regardless of length of stay, even if the hospitalization is a precautionary measure for continued observation. (Note: Hospitalization [including hospitalization for an elective procedure] for a preexisting condition which has not worsened does not constitute a serious adverse event.); or	
	†**Is a congenital anomaly/birth defect** (in offspring of subject taking the product regardless of time to diagnosis);or	
	**Other important medical events** that may not result in death, not be life threatening, or not require hospitalization may be considered a serious adverse event when, based upon appropriate medical judgment, the event may jeopardize the subject and may require medical or surgical intervention to prevent one of the outcomes listed previously (designated above by a †).	
**Duration**	Record the start and stop dates of the adverse event. If less than 1 day, indicate the appropriate length of time and units	
**Action taken**	Did the adverse event cause the Sponsor's product to be discontinued?	
**Relationship to Sponsor's Product**	Did the Sponsor's product cause the adverse event? The determination of the likelihood that the Sponsor's product caused the adverse event will be provided by an investigator who is a qualified physician. The investigator's signed/dated initials on the source document or worksheet that supports the causality noted on the AE form, ensures that a medically qualified assessment of causality was done. This initialed document must be retained for the required regulatory time frame. The criteria below are intended as reference guidelines to assist the investigator in assessing the likelihood of a relationship between the test drug and the adverse event based upon the available information. **The following components are to be used to assess the relationship between the Sponsor's product and the AE**; the greater the correlation with the components and their respective elements (in number and/or intensity), the more likely the Sponsor's product caused the adverse event (AE):	
	**Exposure**	Is there evidence that the subject was actually exposed to the Sponsor's product such as: reliable history, acceptable compliance assessment (pill count, diary, etc.), expected pharmacologic effect, or measurement of drug/metabolite in bodily specimen?
	**Time Course**	Did the AE follow in a reasonable temporal sequence from administration of the Sponsor's product? Is the time of onset of the AE compatible with a drug-induced effect (applies to trials with investigational medicinal product)?
	**Likely Cause**	Is the AE not reasonably explained by another etiology such as underlying disease, other drug(s)/vaccine(s), or other host or environmental factors

**Relationship to Sponsor's Product (continued)**	**The following components are to be used to assess the relationship between the test drug and the AE: (continued)**	
	**Dechallenge**	Was the Sponsor's product discontinued or dose/exposure/frequency reduced?<br>If yes, did the AE resolve or improve?<br>If yes, this is a positive dechallenge. If no, this is a negative dechallenge.<br>(Note: This criterion is not applicable if: (1) the AE resulted in death or permanent disability; (2) the AE resolved/improved despite continuation of the Sponsor's product; or (3) the trial is a single-dose drug trial); or (4) Sponsor's product(s) is/are only used one time.)
	**Rechallenge**	Was the subject re-exposed to the Sponsor's product in this study?<br>If yes, did the AE recur or worsen?<br>If yes, this is a positive rechallenge. If no, this is a negative rechallenge.<br>(Note: This criterion is not applicable if: (1) the initial AE resulted in death or permanent disability, or (2) the trial is a single-dose drug trial); or (3) Sponsor's product(s) is/are used only one time).<br>NOTE: IF A RECHALLENGE IS PLANNED FOR AN ADVERSE EVENT WHICH WAS SERIOUS AND WHICH MAY HAVE BEEN CAUSED BY THE SPONSOR'S PRODUCT, OR IF REEXPOSURE TO THE SPONSOR'S PRODUCT POSES ADDITIONAL POTENTIAL SIGNIFICANT RISK TO THE SUBJECT, THEN THE RECHALLENGE MUST BE APPROVED IN ADVANCE BY THE U.S. CLINICAL MONITOR AS PER DOSE MODIFICATION GUIDELINES IN THE PROTOCOL.
	**Consistency with Trial Treatment Profile**	Is the clinical/pathological presentation of the AE consistent with previous knowledge regarding the Sponsor's product or drug class pharmacology or toxicology?
The assessment of relationship will be reported on the case report forms /worksheets by an investigator who is a qualified physician according to his/her best clinical judgment, including consideration of the above elements.		
**Record one of the following**		**Use the following scale of criteria as guidance (not all criteria must be present to be indicative of a Sponsor's product relationship).**
**Yes, there is a reasonable possibility of Sponsor's product relationship.**		There is evidence of exposure to the Sponsor's product. The temporal sequence of the AE onset relative to the administration of the Sponsor's product is reasonable. The AE is more likely explained by the Sponsor's product than by another cause.
**No, there is not a reasonable possibility of Sponsor's product relationship**		Subject did not receive the Sponsor's product OR temporal sequence of the AE onset relative to administration of the Sponsor's product is not reasonable OR the AE is more likely explained by another cause than the Sponsor's product. (Also entered for a subject with overdose without an associated AE.)

7.2.5 Sponsor Responsibility for Reporting Adverse Events

All Adverse Events will be reported to regulatory authorities, IRB/IECs and investigators in accordance with all applicable global laws and regulations, i.e., per ICH Topic E6 (R1) Guidelines for Good Clinical Practice.

8.0 STATISTICAL ANALYSIS PLAN

8.1 Statistical Analysis Plan Summary

This section contains a brief summary of the statistical analyses for this trial. Full detail is in the Statistical Analysis Plan (SAP) (Section 8.2).

The primary purpose of this study is to:

- investigate the safety, tolerability, and anti-tumor activity of MK-3475 10 mg/kg Q2W administered intravenously to subjects with PD-L1 positive triple negative advanced breast cancer (Cohort A), subjects with PD-L1 positive advanced head and neck cancer (Cohort B), subjects with PD-L1 positive advanced urothelial tract cancer (Cohort C), and subjects with PD-L1 positive advanced gastric cancer (Cohort D).

- investigate the safety, tolerability, and anti-tumor activity of MK-3475 200 mg Q3W administered intravenously to subjects with advanced head and neck cancer irrespective of PD-L1 expression (Cohorts B2).

8.1.1 Efficacy Analyses

The full analysis set (FAS) population (defined as all subjects with a baseline scan with measurable disease; by independent central radiology review for each cohort, and who either have a post baseline scan or discontinue the trial due to progressive disease or a drug-related AE) will serve as the primary population for the analyses of efficacy data in this trial. Supportive analyses of efficacy will be conducted in the all subjects as treated (ASaT) population and the FAS population by Investigator review. For each cohort, overall response rate will be used as the primary endpoint for efficacy assessment. A 95% confidence interval along with a one-sided p-value for testing the null hypothesis based on the binomial distribution will be provided for the response rate in each cohort. The respective cohort is considered to have reached the efficacy objective if the corresponding p-value for testing the respective null hypothesis is less than 2.5%. An outline of the efficacy analysis strategy is presented in Table 9 below.

Table 9 Primary Analysis Strategy for Efficacy Endpoints

**Endpoint/Variable‡ (Description, Time Point)**	**Statistical Method**	**Analysis Population**	**Missing Data Approach**
Primary:			
Overall RECIST 1.1 response rate based on independent central radiology review (Cohort A, Cohort B HPV negative subjects, Cohort C, and Cohort D evaluated separately)	Exact test of binomial parameter	FAS	Subjects with missing data are considered non-responders
Overall RECIST 1.1 response rate based on independent central radiology review for subjects in Cohort B2	Exact test of binomial parameter	FAS	Subjects with missing data are considered non-responders
Secondary:			
Overall RECIST1.1 response rate based on independent central radiology review, Cohort B HPV positive subjects,	Exact test of binomial parameter	FAS	Subjects with missing data are considered non-responders
Overall RECIST 1.1 response rate based on independent central radiology review, Cohort D AP subjects.	Exact test of binomial parameter	FAS	Subjects with missing data are considered non-responders
Overall RECIST 1.1 response rate based on based on independent central radiology review, for subjects previously treated with cetuximab and platinum in Cohorts B and B2	Exact test of binomial parameter	FAS	Subjects with missing data are considered non-responders
Overall RECIST 1.1 response rate based on investigator assessment for cohorts A,B, C and D	Exact methods for binomial parameter	FAS	Subjects with missing data are considered non-responders
Overall RECIST 1.1 response rate based on investigator assessment for Cohort B2	Exact methods for binomial parameter	FAS	Subjects with missing data are considered non-responders
Progression-free survival	Summary statistics using Kaplan-Meier method	FAS	Censored at last assessment
Overall survival	Summary statistics using Kaplan-Meier method	FAS	Censored at last assessment
Response duration	Summary statistics using Kaplan-Meier method	All responders	Non-responders are excluded in analysis
‡ For Cohort D, the analyses for the Asia Pacific (AP) population will be performed as appropriate.			

8.1.2 Safety Analyses

The All-Subjects-as-Treated population will be employed for safety analyses.

8.1.3 Power and Sample Size

The calculation of power and sample size for each cohort does not account for interim analysis. Cohort A (triple negative breast cancer subjects): With approximately 26 evaluable PD-L1 positive subjects with triple negative breast cancer, the study has approximately 80% power to detect a 25% difference in ORR under the null hypothesis of ORR=20% with a type I error rate of 2.5% if the true ORR is 45%. Success for this hypothesis requires at least 10/26 responses. The actual number of subjects enrolled may be larger than 26 to ensure that at least 26 subjects are evaluable for analysis.

Cohort B (head and neck cancer subjects): HPV negative head and neck cancer subjects will be evaluated separately from HPV positive head and neck cancer subjects. With a maximum of 22 evaluable PD-L1 positive subjects with HPV negative head and neck cancer, the study has approximately 80% power to detect a 25% difference in ORR under the null hypothesis of ORR=10% with a type I error rate of 2.5% if the true ORR is 35%. Success for this hypothesis requires at least 6/22 responses. The actual number of subjects enrolled may be larger than 22 to ensure that at least 22 subjects are evaluable for analysis.

With a maximum of 12 evaluable PD-L1 positive subjects with HPV positive head and neck cancer, the study has approximately 73% power to detect a 35% difference in ORR under the null ORR=20% with a type I error rate of 5% if the true ORR is 55%. Success for this hypothesis requires at least 6 responses. The actual number of subjects enrolled may be larger than 12 to ensure that at least 12 subjects are evaluable for analysis. However, if 6 responses are observed prior to 12 subjects with HPV positive head and neck cancer enrolling in the trial, enrollment may be stopped in this group as the hypothesis success criterion will have been reached.

Cohort C (urothelial tract cancer subjects): With a maximum of 22 evaluable PD-L1 positive subjects with urothelial tract cancer, the study has approximately 80% power to detect a 25% difference in ORR under the null hypothesis of ORR=10% with a type I error rate of 2.5% if the true ORR is 35%. Success for this hypothesis requires at least 6/22 responses. The actual number of subjects enrolled may be larger than 22 to ensure that at least 22 subjects are evaluable for analysis.

Confidential

Cohort D (gastric cancer subjects): With a maximum of 32 evaluable PD-L1 positive subjects with gastric cancer, stratified to include 16 Asia Pacific (AP) and 16 non-AP, the study has approximately 90% power to detect a 25% difference in ORR under the null hypothesis of ORR=15% with a type I error rate of 2.5% if the true ORR is 40%. Success for this hypothesis requires at least 10/32 responses. A total of 16 AP subjects will provide approximately 80% power with a type I error rate of 0.1 to detect a 25% difference in ORR under the null hypothesis of ORR=15% if the true ORR is 40%. At least 4/16 responses will be needed to claim the success of this hypothesis for AP population. The actual number of subjects enrolled may be larger than 32 to ensure that at least 32 subjects are evaluable for analysis.

Cohort B2 (head and neck cancer subjects, expansion cohort): With 100 evaluable subjects in Cohort B2, the study provides >99% power to detect a 15% difference in ORR under the null hypothesis of ORR=5% with a type I error rate of 2.5% if the true ORR is 20%. Success for this hypothesis requires at least 11/100 responses. The actual number of subjects enrolled may be larger than 100 to ensure at least 100 subjects are evaluable for analysis.

Cohorts B and B2 previously treated with cetuximab and platinum: With 60 evaluable head and neck cancer subjects previously treated with cetuximab and platinum, the study has 93% power to detect a 15% difference in ORR under the null hypothesis of ORR=5% with a type I error rate of 2.5% if the true ORR is 20%. Success for this hypothesis requires at least 8/60 responses.

8.1.4 Interim Analysis

No efficacy interim analyses are planned for Cohort B2 or Cohort D in this trial. However, an interim analysis for Cohort A, B or C may be performed if the rate of enrollment is much slower than anticipated during the course of the trial. An interim analysis for each cohort would only be performed in this study when ≥10 subjects in the respective cohort have had post-baseline scans through Week 16. Results will be reviewed by the study team. Table 10 summarizes the strategy and timing of the potential interim analysis for each cohort.

For Cohort A, if an interim analysis is conducted and 2 or fewer subjects out of the first 10 subjects with post-baseline imaging scans (Week 16) have a confirmed or unconfirmed response, then enrollment may be paused until response data for the subsequent imaging scan (Week 24) are reviewed for subjects already enrolled in the trial. Enrollment may be resumed if the binomial probability of the observed response rate is ≥ 80% under the assumption of a ≥ 20% true response rate (e.g., ≥4/16 confirmed or unconfirmed response).

For Cohorts B (HPV negative subjects only) and C, if an interim analysis is conducted and 1 or fewer subjects out of the first 10 subjects with post-baseline imaging scans (Week 16) have a confirmed or unconfirmed response, then enrollment may be paused until response data for the subsequent imaging scan (Week 24) are reviewed for subjects already enrolled in the trial. Enrollment may be resumed if the binomial probability of the observed response rate is ≥ 80% under the assumption of a ≥ 10% true response rate (e.g., ≥2/16 confirmed or unconfirmed response). No interim analysis will be performed for the subjects with HPV positive head and neck cancer in Cohort B.

Although safety monitoring will occur continuously in the study, an assessment of Grade 4/5 drug-related immunologic AEs will be performed after 10 subjects have been enrolled within each cohort for 1 cycle of therapy. Enrollment will be paused if 4 or more subjects out of the first 10 subjects within a cohort experience a Grade 4/5 drug-related immunologic adverse event. Enrollment may be resumed within a cohort only after a full safety evaluation is performed in consultation between the study investigators and the Sponsor.

Table 10 Summary of Interim Analysis Strategy

Interim Analysis Number	Key Endpoints for Interim Analysis	Timing (Sample Size) for Analysis	Purpose of Analysis
Interim Analysis Cohort A	• ORR	• ≥10 subjects with post-baseline scans (Week 16)	• Pause enrollment until further evidence of efficacy<br>• Pause enrollment for safety based on Grade 4+ drug-related immunologic AEs out of first 10 subjects
Interim Analysis Cohort B (HPV negative subjects only)	• ORR	• ≥10 subjects with post-baseline scans (Week 16)	• Pause enrollment until further evidence of efficacy<br>• Pause enrollment for safety based on Grade 4+ drug-related immunologic AEs out of first 10 subjects
Interim Analysis Cohort C	• ORR	• ≥10 subjects with post-baseline scans (Week 16)	• Pause enrollment until further evidence of efficacy<br>• Pause enrollment for safety based on Grade 4+ drug-related immunologic AEs out of first 10 subjects

8.2 Statistical Analysis Plan

8.2.1 Responsibility for Analyses/In-House Blinding

The statistical analysis of the data obtained from this study will be the responsibility of the Clinical Biostatistics department of the SPONSOR.

This trial is being conducted as an open-label study, i.e., subjects, investigators, and SPONSOR personnel will be aware of subject treatment assignments after each subject is enrolled and treatment is assigned. Access to the PD-L1 subject-level biomarker results for subjects in Cohort B2 for the purpose of summary reporting of PD-L1 level until the time that a clinical study report (CSR) is generated will be limited to an unblinded SPONSOR statistician and unblinded SPONSOR statistical programmer who will be responsible for data review to ensure validity of results and summary reporting of clinical response by biomarker status, but who will have no other responsibilities associated with the study.

The Clinical Biostatistics department will generate the allocation schedule(s) for study treatment assignment. Allocation will be implemented in an interactive voice response system (IVRS).

8.2.2 Hypotheses/Estimation

Objectives and hypotheses of the study are stated in Section 3.0.

8.2.3 Analysis Endpoints

Efficacy and safety endpoints that will be evaluated for within- and/or between-treatment differences are listed below, followed by the descriptions of the derivations of selected endpoints.

8.2.3.1 Efficacy/Immunogenicity/Pharmacokinetics Endpoints

Efficacy and safety endpoints that will be evaluated for are listed below, followed by the descriptions of the derivations of selected endpoints.

The primary efficacy endpoint is response rate, defined as the proportion of subjects in the analysis population who have complete response (CR) or partial response (PR) using RECIST 1.1 criteria at any time during the study. Response for the primary analysis for cohorts A, B, C, D and B2 will be determined by a central independent radiology review. Secondary analyses will be based on the responses as assessed by the investigator.

Key secondary efficacy endpoints include:

- RECIST 1.1 response rate among HPV positive subjects.
- Response rate based on site assessments using RECIST 1.1.

Other secondary efficacy endpoints include: (1) duration of response, defined as time from first RECIST 1.1 response to disease progression in subjects who achieve a PR or better; (2) progression-free survival (PFS), defined as the time from allocation to the first documented disease progression according to RECIST 1.1 or death due to any cause, whichever occurs first; and (3) overall survival (OS).

8.2.3.2 Safety Endpoints

A description of safety measures is provided in Section 4.2.3.2.

The primary safety endpoints are AEs graded using CTCAE (Version 4.0) criteria. Safety will be assessed by quantifying the toxicities and grades experienced by subjects who have received MK-3475, including serious adverse events (SAEs) and events of clinical interest (ECIs). Immune-related ECIs, as described in Section 7.2.3.2 will be collected. Other safety endpoints include laboratory safety assessments, ECOG performance status, vital signs and physical examinations.

8.2.4 Analysis Populations

8.2.4.1 Efficacy Analysis Populations

The Full Analysis Set (FAS) population will serve as the primary population for the analysis of efficacy data in this study. The FAS population consists of all randomized subjects in each Cohort who:

- Receive at least one dose of study treatment,
- Have a baseline scan with measurable disease per RECIST 1.1 by independent central radiology review, and
- Have a post baseline scan OR discontinue the trial due to progressive disease/drug related AE

For secondary by Investigator assessment, measurable disease for inclusion in the FAS population will be defined using Investigator assessment of the baseline scan.

A supportive analysis using the All Subjects as Treated (ASaT) population, defined as all randomized subjects who received at least one dose of study treatment, will be performed for the primary efficacy endpoint(s).

Subjects will be included in the treatment group to which they are randomized for the analysis of efficacy data. Details on the approach to handling missing data are provided in Section 8.2.5 Statistical Methods.

8.2.4.2 Safety Analysis Populations

The All Subjects as Treated (ASaT) population will be used for the analysis of safety data in this study. The ASaT population consists of all allocated subjects who received at least one dose of study treatment.

At least one laboratory or vital sign measurement obtained subsequent to at least one dose of study treatment is required for inclusion in the analysis of each specific parameter. To assess change from baseline, a baseline measurement is also required.

Details on the approach to handling missing data for safety analyses are provided in Section 8.2.5 Statistical Methods.

8.2.5 Statistical Methods

Statistical testing and inference for safety analyses are described in 8.2.5.2. Efficacy results that will be considered to be statistically significant after consideration of the strategy for controlling the Type I error are described in Section 8.2.6, Multiplicity. Nominal p-values may be computed for other efficacy analyses as a measure of strength of association between the endpoint and the treatment effect rather than formal tests of hypotheses. Unless otherwise stated, all statistical tests will be conducted at the $\alpha=0.05$ (2-sided) level.

8.2.5.1 Statistical Methods for Efficacy Analyses

Efficacy will be evaluated separately in each cohort. For the primary efficacy endpoint independent centrally reviewed RECIST 1.1 response rate, the point estimate, exact 95% Clopper-Pearson confidence interval, and p-value for testing the RECIST 1.1 response rate is greater than the historical control for each cohort will be provided using exact binomial distribution. Subjects in the primary analysis population (FAS) without response data will be counted as non-responder.

Secondary efficacy evaluations of RECIST 1.1 response based on Investigator assessment will also be conducted using the same methodology as for the primary efficacy analysis. Efficacy for the secondary analysis of RECIST 1.1 response by independent central radiology review among subjects with HPV positive head and neck cancer will be evaluated in the same manner as in the primary analysis.

For PFS endpoint, Kaplan-Meier (KM) curves and median estimates from the KM curves will be provided as appropriate. Subjects without efficacy evaluation data or without survival data will be censored at Day 1. Table 11 summarizes the key efficacy analyses.

Table 11 Analysis Strategy for Key Efficacy Variables

**Endpoint/Variable**[‡] **(Description, Time Point)**	**Statistical Method**	**Analysis Population**	**Missing Data Approach**
Overall RECIST 1.1 response rate based on independent central radiology review (Cohort A, Cohort B HPV negative subjects, Cohort C, and Cohort D evaluated separately)	Exact test of binomial parameter	FAS (Primary) ASaT (Supportive)	Subjects with missing data are considered non-responders
Overall RECIST 1.1 response rate based on independent central radiology review for subjects in Cohort B2	Exact test of binomial parameter	FAS (Primary) ASaT (Supportive)	Subjects with missing data are considered non-responders
Overall RECIST1.1 response rate based on independent central radiology review, Cohort B HPV positive subjects,	Exact test of binomial parameter	FAS (Primary) ASaT (Supportive)	Subjects with missing data are considered non-responders
Overall RECIST 1.1 response rate based on independent central radiology review, Cohort D AP subjects.	Exact test of binomial parameter	FAS (Primary) ASaT (Supportive)	Subjects with missing data are considered non-responders
Overall RECIST 1.1 response rate based on based on independent central radiology review, for subjects previously treated with cetuximab and platinum in Cohorts B and B2	Exact test of binomial parameter	FAS (Primary) ASaT (Supportive)	Subjects with missing data are considered non-responders
Overall RECIST 1.1 response rate based on investigator assessment for cohorts A,B, C and D	Exact methods for binomial parameter	FAS (Primary) ASaT (Supportive)	Subjects with missing data are considered non-responders
Overall RECIST 1.1 response rate based on investigator assessment for Cohort B2	Exact methods for binomial parameter	FAS (Primary) ASaT (Supportive)	Subjects with missing data are considered non-responders
Progression-free survival	Summary statistics using Kaplan-Meier method	FAS(Primary) ASaT (Supportive)	Censored at last assessment
Overall survival	Summary statistics using Kaplan-Meier method	FAS(Primary) ASaT (Supportive)	Censored at last assessment
Response duration	Summary statistics using Kaplan-Meier method	All responders	Non-responders are excluded in analysis

The strategy to address multiplicity issues with regard to multiple efficacy endpoints is described in Section 8.2.6, Multiplicity and Section 8.2.9, Interim Analyses.

8.2.5.2 Statistical Methods for Safety Analyses

Safety and tolerability will be assessed by clinical review of all relevant parameters including adverse experiences (AEs), laboratory tests, and vital signs.

Summary statistics (counts, percentage, mean, standard deviation, etc.) will be provided for the safety endpoints as appropriate. The 80% confidence interval for the incidence rate of Grade 2 or higher adverse events with an immune etiology and the incidence rate of Grade 4/5 AEs will be provided as appropriate.

8.2.5.3 Summaries of Baseline Characteristics, Demographics, and Other Analyses

8.2.5.3.1 Demographic and Baseline Characteristics

Baseline characteristics will be assessed by the use of tables and/or graphs for each cohort. No statistical hypothesis tests will be performed on these characteristics. The number and percentage of subjects screened, randomized, the primary reasons for screening failure, and the primary reason for discontinuation will be displayed. Demographic variables (e.g., age, gender), baseline characteristics, primary and secondary diagnoses, and prior and concomitant therapies will be summarized by treatment either by descriptive statistics or categorical tables.

8.2.6 Multiplicity

The false positive rate for testing the primary efficacy endpoint in each cohort (Cohort A, Cohort B, Cohort C, Cohort D, and Cohort B2) is controlled at 0.025 (1-sided) for each cohort.

If the primary hypothesis in Cohort B2 is successful, then alpha=0.025 (1-sided) will be passed to the secondary evaluation of subjects in Cohort B and Cohort B2 with prior exposure to cetuximab and platinum therapy in a step down approach.

8.2.7 Sample Size and Power Calculations

The calculation of power and sample size for each cohort does not account for interim analysis. Cohort A (triple negative breast cancer subjects): With approximately 26 evaluable PD-L1 positive subjects with triple negative breast cancer, the study has approximately 80% power to detect a 25% difference in ORR under the null hypothesis of ORR=20% with a type I error rate of 2.5% if the true ORR is 45%. The null hypothesis rate of 20% is based on the historic response rate in large phase III trials for standard single agent chemotherapy in 2nd line breast cancer utilizing WHO-based bi-dimensional responses [34, 35, 36]. Success for this hypothesis requires at least 10/26 responses. The actual number of subjects enrolled may be larger than 26 to ensure that at least 26 subjects are evaluable for analysis.

Cohort B (head and neck cancer subjects): HPV negative head and neck cancer subjects will be evaluated separately from HPV positive head and neck cancer subjects. With a maximum of 22 evaluable PD-L1 positive subjects with HPV negative head and neck cancer, the study has approximately 80% power to detect a 25% difference in ORR under the null hypothesis of ORR=10% with a type I error rate of 2.5% if the true ORR is 35%. Success for this hypothesis requires at least 6/22 responses. The actual number of subjects enrolled may be larger than 22 to ensure that at least 22 subjects are evaluable for analysis.

With a maximum of 12 evaluable PD-L1 positive subjects with HPV positive head and neck cancer, the study has approximately 73% power to detect a 35% difference in ORR under the null ORR=20% with a type I error rate of 5% if the true ORR is 55%. The null hypothesis rate of 20% is based on historic response of chemotherapy in head and neck cancer trials [37, 38]. Success for this hypothesis requires at least 6 responses. The actual number of subjects enrolled may be larger than 12 to ensure that at least 12 subjects are evaluable for analysis. However, if 6 responses are observed prior to 12 subjects with HPV positive head and neck cancer enrolling in the trial, enrollment may be stopped in this group as the hypothesis success criterion will have been reached.

Cohort C (urothelial tract cancer subjects): With a maximum of 22 evaluable PD-L1 positive subjects with urothelial tract cancer, the study has approximately 80% power to detect a 25% difference in ORR under the null hypothesis of ORR=10% with a type I error rate of 2.5% if the true ORR is 35%. The null hypothesis rate of 10% is based on the historic response rate of single agent pemetrexed (RR 6%) or vinflunine (RR 9%) chemotherapy for bladder cancer [39, 40]. Success for this hypothesis requires at least 6/22 responses. The actual number of subjects enrolled may be larger than 22 to ensure that at least 22 subjects are evaluable for analysis.

Cohort D (gastric cancer subjects): With a maximum of 32 evaluable PD-L1 positive subjects with gastric cancer, stratified to include 16 Asia Pacific (AP) and 16 non-AP, the study has approximately 90% power to detect a 25% difference in ORR under the null hypothesis of ORR=15% with a type I error rate of 2.5% if the true ORR is 40%. Success for this hypothesis requires at least 10/32 responses. A total of 16 AP subjects will provide approximately 80% power with type I error rate of 0.1 to detect a 25% difference in ORR under the null hypothesis of ORR=15% if the true ORR is 40%. At least 4/16 responses will be needed to claim the success of this hypothesis for the AP population. The null hypothesis rate of 15% is based on the response rate in large (n > 100), trials for standard of care chemotherapy irinotecan/mFOLFIRI (RR 12%), and irinotecan/docetaxel (RR 13%) for 2nd line metastatic gastric cancer [28, 29]. The actual number of subjects enrolled may be larger than 32 to ensure that at least 32 subjects are evaluable for analysis.

Cohort B2 (head and neck cancer subjects, expansion cohort): With 100 evaluable subjects in Cohort B2, the study provides >99% power to detect a 15% difference in ORR under the null hypothesis of ORR=5% with a type I error rate of 2.5% if the true ORR is 20%. Success for this hypothesis requires at least 11/100 responses. The null hypothesis rate of 5% is based on historic response of chemotherapy in head and neck cancer trials [37]. The actual number of subjects enrolled may be larger than 100 to ensure at least 100 subjects are evaluable for analysis.

Cohorts B and B2 previously treated with cetuximab and platinum: With 60 evaluable head and neck cancer subjects previously treated with cetuximab and platinum, the study has 93% power to detect a 15% difference in ORR under the null hypothesis of ORR=5% with a type I error rate of 2.5% if the true ORR is 20%. Success for this hypothesis requires at least 8/60 responses. The null hypothesis rate of 5% is based on historic response of chemotherapy in head and neck cancer trials [37].

8.2.8 Subgroup Analyses and Effect of Baseline Factors

Efficacy will be summarized for the key endpoints for the following subgroups:

- HPV-positive vs. HPV negative head/neck cancer
- Subjects with head/neck cancer enrolled in Cohort B and Cohort B2 with prior cetuximab and platinum exposure.

8.2.9 Interim Analyses

8.2.9.1 Efficacy Interim Analyses

No efficacy interim analyses are planned in this trial.

However, an interim analysis for Cohort A, B, or C may be performed if the rate of enrollment is much slower than anticipated during the course of the trial. An interim analysis for each cohort would only be performed in this study when ≥10 subjects in the respective cohort have had scans through Week 16. Results will be reviewed by the study team.

For Cohort A, if an interim analysis is conducted and 2 or fewer subjects out of the first 10 subjects with post-baseline imaging scans (Week 16) have a confirmed or unconfirmed response, then enrollment may be paused until response data for the subsequent imaging scan (Week 24) are reviewed for subjects already enrolled in the trial. Enrollment may be resumed if the binomial probability of the observed response rate is ≥ 80% under the assumption of a ≥ 20% true response rate (e.g., ≥4/16 confirmed or unconfirmed response). The operating characteristics of the interim analysis rule are provided in Table 12. The power in Cohort A (assuming true ORR of 45%) decreases to ~77% if the interim analysis is conducted.

Table 12 Operating Characteristics of Interim Analysis Rule for Triple Negative Breast Cancer – Pause Enrollment if ≤ 2/10 Subjects Respond

True ORR	Probability of Pausing Enrollment	Probability of Study Success
10%	0.93	<0.01
15%	0.82	<0.01
20%	0.68	0.02
25%	0.53	0.08
30%	0.38	0.21
35%	0.26	0.39
40%	0.17	0.60
45%	0.10	0.77
50%	0.06	0.88

For Cohorts B (HPV negative) and C, if an interim analysis is conducted and 1 or fewer subjects out of the first 10 subjects with post-baseline imaging scans (Week 16) have a confirmed or unconfirmed response, then enrollment may be paused until response data for the subsequent imaging scan (Week 24) are reviewed for subjects already enrolled in the trial. Enrollment may be resumed if the binomial probability of the observed response rate is ≥ 80% under the assumption of a ≥ 10% true response rate (e.g., ≥2/16 confirmed or unconfirmed response). The operating characteristics of the interim analysis rule are provided in Table 13. The power in Cohort B (HPV negative) and Cohort C (assuming true ORR of 35%) does not substantially decrease from 80% as a result of conducting the interim analysis.

Table 13 Operating Characteristics of Interim Analysis Rule for Head/Neck Cancer (HPV negative) and Urothelial Cancer– Pause Enrollment if ≤ 1/10 subjects respond

True ORR	Probability of Pausing Enrollment	Probability of Study Success
5%	0.91	<0.01
10%	0.74	0.02
15%	0.54	0.09
20%	0.38	0.25
25%	0.25	0.45
30%	0.15	0.65
35%	0.09	0.80
40%	0.05	0.90
45%	0.02	0.95

8.2.9.2 Safety Interim Analyses

Although safety monitoring will occur continuously in the study, an assessment of Grade 4/5 drug-related immunologic AEs will be performed after 10 subjects have been enrolled within each cohort for 1 cycle of therapy. Enrollment will be paused if 4 or more subjects out of the first 10 subjects within a cohort experience a Grade 4/5 drug-related immunologic adverse event. Enrollment may be resumed within a cohort only after a full safety evaluation is performed in consultation between the study investigators and the Sponsor.

The estimate of and the upper bound of the 95% confidence interval for the underlying percentage of subjects with a Grade 4/5 drug-related immunologic adverse event given various hypothetical observed number of subjects are provided in Table 14. These calculations are based on the exact binomial method proposed by Clopper and Pearson [41].

Table 14 Estimate of Incidence of Grade 4/5 events and 95% Upper Confidence Bound Based on Hypothetical Number of Subjects with Event Out of 10 Subjects Evaluated

Hypothetical Number of Subjects With Grade 4/5 Event	Estimate of Incidence	95% Upper Confidence Bound†
0	0%	30.9%
1	10%	44.5%
2	20%	55.6%
3	30%	65.2%
4	40%	73.8%
5	50%	81.3%

† Based on the two-tailed exact confidence interval of a binomial proportion [41].

8.2.10 Compliance (Medication Adherence)

A day within the study will be considered an On-Therapy day if the subject receives the study medication infusion. The number of days on therapy is the total number of days from the first day of study medication to the date of the last dose of study medication. Compliance with trial treatment administration will be measured by the number of administered infusions divided by the number of infusions that were supposed to be administered as determined by the number of days on therapy.

Summary statistics for the number of Days on Therapy will be provided by treatment group for the FAS population.

8.2.11 Extent of Exposure

Extent of Exposure for a subject is defined as number of cycles in which the subject receives the study medication infusion. Summary statistics will be provided on Extent of Exposure for ASaT population.

9.0 LABELING, PACKAGING, STORAGE AND RETURN OF CLINICAL SUPPLIES

9.1 Investigational Product

The investigator shall take responsibility for and shall take all steps to maintain appropriate records and ensure appropriate supply, storage, handling, distribution and usage of investigational product in accordance with the protocol and any applicable laws and regulations.

Clinical Supplies will be provided by the Sponsor as summarized in Table 15.

Table 15 Product Descriptions

Product Name & Potency	Dosage Form
MK-3475 50 mg	Lyophilized Powder for Injection
MK-3475 100 mg/ 4mL	Solution for Injection

9.2 Packaging and Labeling Information

Clinical supplies will be affixed with a clinical label in accordance with regulatory requirements.

Vials will be provided in an open label fashion for subject dosing.

9.3 Clinical Supplies Disclosure

This trial is open-label; therefore, the subject, the trial site personnel, the Sponsor and/or designee are not blinded to treatment. Drug identity (name, strength) is included in the label text; random code/disclosure envelopes or lists are not provided.

9.4 Storage and Handling Requirements

Clinical supplies must be stored in a secure, limited-access location under the storage conditions specified on the label.

Receipt and dispensing of trial medication must be recorded by an authorized person at the trial site.

Clinical supplies may not be used for any purpose other than that stated in the protocol.

9.5 Returns and Reconciliation

The investigator is responsible for keeping accurate records of the clinical supplies received from the Sponsor or designee, the amount dispensed to and returned by the subjects and the amount remaining at the conclusion of the trial.

For all trial sites, the local country Sponsor personnel or designee will provide appropriate documentation that must be completed for drug accountability and return.

9.6 Standard Policies

Trial site personnel will have access to a central electronic randomization system (IVRS/IWRS system) to allocate subjects, to assign drug to subjects and to manage the distribution of clinical supplies. Each person accessing the IVRS system must be assigned an individual unique PIN. They must use only their assigned PIN to access the system, and they must not share their assigned PIN with anyone.

10.0 ADMINISTRATIVE AND REGULATORY DETAILS

10.1 Confidentiality

10.1.1 Confidentiality of Data

By signing this protocol, the investigator affirms to the Sponsor that information furnished to the investigator by the Sponsor will be maintained in confidence, and such information will be divulged to the institutional review board, ethics review committee (IRB/ERC) or similar or expert committee; affiliated institution and employees, only under an appropriate understanding of confidentiality with such board or committee, affiliated institution and employees. Data generated by this trial will be considered confidential by the investigator, except to the extent that it is included in a publication as provided in the Publications section of this protocol.

10.1.2 Confidentiality of Subject Records

By signing this protocol, the investigator agrees that the Sponsor (or Sponsor representative), IRB/ERC, or regulatory authority representatives may consult and/or copy trial documents in order to verify worksheet/case report form data. By signing the consent form, the subject agrees to this process. If trial documents will be photocopied during the process of verifying worksheet/case report form information, the subject will be identified by unique code only; full names/initials will be masked prior to transmission to the Sponsor.

By signing this protocol, the investigator agrees to treat all subject data used and disclosed in connection with this trial in accordance with all applicable privacy laws, rules and regulations.

10.1.3 Confidentiality of Investigator Information

By signing this protocol, the investigator recognizes that certain personal identifying information with respect to the investigator, and all subinvestigators and trial site personnel, may be used and disclosed for trial management purposes, as part of a regulatory submissions, and as required by law. This information may include:

1. name, address, telephone number and e-mail address;
2. hospital or clinic address and telephone number;
3. curriculum vitae or other summary of qualifications and credentials; and
4. other professional documentation.

Consistent with the purposes described above, this information may be transmitted to the Sponsor, and subsidiaries, affiliates and agents of the Sponsor, in your country and other countries, including countries that do not have laws protecting such information. Additionally, the investigator's name and business contact information may be included when reporting certain serious adverse events to regulatory authorities or to other investigators. By signing this protocol, the investigator expressly consents to these uses and disclosures.

If this is a multicenter trial, in order to facilitate contact between investigators, the Sponsor may share an investigator's name and contact information with other participating investigators upon request.

10.1.4 Confidentiality of IRB/IEC Information

The Sponsor is required to record the name and address of each IRB/IEC member that reviews and approves this trial. The Sponsor is also required to document that each IRB/IEC meets regulatory and ICH GCP requirements by requesting and maintaining records of the names and qualifications of the IRB/IEC members and to make these records available for regulatory agency review upon request by those agencies.

10.2 Compliance with Financial Disclosure Requirements

Financial Disclosure requirements are outlined in the US Food and Drug Administration Regulations, Financial Disclosure by Clinical Investigators (21 CFR Part 54). It is the Sponsor's responsibility to determine, based on these regulations, whether a request for Financial Disclosure information is required. It is the investigator's/subinvestigator's responsibility to comply with any such request.

The investigator/subinvestigator(s) agree, if requested by the Sponsor in accordance with 21 CFR Part 54, to provide his/her financial interests in and/or arrangements with the Sponsor to allow for the submission of complete and accurate certification and disclosure statements. The investigator/subinvestigator(s) further agree to provide this information on a Certification/Disclosure Form, commonly known as a financial disclosure form, provided by the Sponsor or through a secure password-protected electronic portal provided by the Sponsor. The investigator/subinvestigator(s) also consent to the transmission of this information to the Sponsor in the United States for these purposes. This may involve the transmission of information to countries that do not have laws protecting personal data.

10.3 Compliance with Law, Audit and Debarment

By signing this protocol, the investigator agrees to conduct the trial in an efficient and diligent manner and in conformance with this protocol; generally accepted standards of Good Clinical Practice (e.g., International Conference on Harmonization of Technical Requirements for Registration of Pharmaceuticals for Human Use Good Clinical Practice: Consolidated Guideline and other generally accepted standards of good clinical practice); and all applicable federal, state and local laws, rules and regulations relating to the conduct of the clinical trial.

The Code of Conduct, a collection of goals and considerations that govern the ethical and scientific conduct of clinical investigations sponsored by Merck, is provided in Section 12.1 - Merck Code of Conduct for Clinical Trials.

The investigator also agrees to allow monitoring, audits, IRB/ERC review and regulatory authority inspection of trial-related documents and procedures and provide for direct access to all trial-related source data and documents.

The investigator agrees not to seek reimbursement from subjects, their insurance providers or from government programs for procedures included as part of the trial reimbursed to the investigator by the Sponsor.

The investigator shall prepare and maintain complete and accurate trial documentation in compliance with Good Clinical Practice standards and applicable federal, state and local laws, rules and regulations; and, for each subject participating in the trial, provide all data, and, upon completion or termination of the clinical trial, submit any other reports to the Sponsor as required by this protocol or as otherwise required pursuant to any agreement with the Sponsor.

Trial documentation will be promptly and fully disclosed to the Sponsor by the investigator upon request and also shall be made available at the trial site upon request for inspection, copying, review and audit at reasonable times by representatives of the Sponsor or any regulatory authorities. The investigator agrees to promptly take any reasonable steps that are requested by the Sponsor as a result of an audit to cure deficiencies in the trial documentation and worksheets/case report forms.

The investigator must maintain copies of all documentation and records relating to the conduct of the trial in compliance with all applicable legal and regulatory requirements. This documentation includes, but is not limited to, the protocol, worksheets/case report forms, advertising for subject participation, adverse event reports, subject source data, correspondence with regulatory authorities and IRBs/ERCs, consent forms, investigator's curricula vitae, monitor visit logs, laboratory reference ranges, laboratory certification or quality control procedures and laboratory director curriculum vitae. By signing this protocol, the investigator agrees that documentation shall be retained until at least 2 years after the last approval of a marketing application in an ICH region or until there are no pending or contemplated marketing applications in an ICH region or until at least 2 years have elapsed since the formal discontinuation of clinical development of the investigational product. Because the clinical development and marketing application process is variable, it is anticipated that the retention period can be up to 15 years or longer after protocol database lock. The Sponsor will determine the minimum retention period and notify the investigator when documents may be destroyed. The sponsor also recognizes that documents may need to be retained for a longer period if required by local regulatory requirements. All trial documents shall be made available if required by relevant regulatory authorities. The investigator must consult with and obtain written approval by the Sponsor prior to discarding trial and/or subject files.

ICH Good Clinical Practice guidelines recommend that the investigator inform the subject's primary physician about the subject's participation in the trial if the subject has a primary physician and if the subject agrees to the primary physician being informed.

The investigator will promptly inform the Sponsor of any regulatory authority inspection conducted for this trial.

Persons debarred from conducting or working on clinical trials by any court or regulatory authority will not be allowed to conduct or work on this Sponsor's trials. The investigator will immediately disclose in writing to the Sponsor if any person who is involved in conducting the trial is debarred or if any proceeding for debarment is pending or, to the best of the investigator's knowledge, threatened.

In the event the Sponsor prematurely terminates a particular trial site, the Sponsor will promptly notify that trial site's IRB/IEC.

According to European legislation, a Sponsor must designate an overall coordinating investigator for a multi-center trial (including multinational). When more than one trial site is open in an EU country, Merck, as the Sponsor, will designate, per country, a national principal coordinator (Protocol CI), responsible for coordinating the work of the principal investigators at the different trial sites in that Member State, according to national regulations. For a single-center trial, the Protocol CI is the principal investigator. In addition, the Sponsor must designate a principal or coordinating investigator to review the trial report that summarizes the trial results and confirm that, to the best of his/her knowledge, the report accurately describes the conduct and results of the trial [Clinical Study Report (CSR) CI]. The Sponsor may consider one or more factors in the selection of the individual to serve as the Protocol CI and or CSR CI (e.g., availability of the CI during the anticipated review process, thorough understanding of clinical trial methods, appropriate enrollment of subject cohort, timely achievement of trial milestones). The Protocol CI must be a participating trial investigator.

10.4 Compliance with Trial Registration and Results Posting Requirements

Under the terms of the Food and Drug Administration Modernization Act (FDAMA) and the Food and Drug Administration Amendments Act (FDAAA), the Sponsor of the trial is solely responsible for determining whether the trial and its results are subject to the requirements for submission to the Clinical Trials Data Bank, http://www.clinicaltrials.gov. Merck, as Sponsor of this trial, will review this protocol and submit the information necessary to fulfill these requirements. Merck entries are not limited to FDAMA/FDAAA mandated trials. Information posted will allow subjects to identify potentially appropriate trials for their disease conditions and pursue participation by calling a central contact number for further information on appropriate trial locations and trial site contact information.

By signing this protocol, the investigator acknowledges that the statutory obligations under FDAMA/FDAAA are that of the Sponsor and agrees not to submit any information about this trial or its results to the Clinical Trials Data Bank.

10.5 Quality Management System

By signing this protocol, the Sponsor agrees to be responsible for implementing and maintaining a quality management system with written development procedures and functional area standard operating procedures (SOPs) to ensure that trials are conducted and data are generated, documented, and reported in compliance with the protocol, accepted standards of Good Clinical Practice, and all applicable federal, state, and local laws, rules and regulations relating to the conduct of the clinical trial.

10.6 Data Management

The investigator or qualified designee is responsible for recording and verifying the accuracy of subject data. By signing this protocol, the investigator acknowledges that his/her electronic signature is the legally binding equivalent of a written signature. By entering his/her electronic signature, the investigator confirms that all recorded data have been verified as accurate.

Detailed information regarding Data Management procedures for this protocol will be provided by the Sponsor.

10.7 Publications

This trial is intended for publication, even if terminated prematurely. Publication may include any or all of the following: posting of a synopsis online, abstract and/or presentation at a scientific conference, or publication of a full manuscript. The Sponsor will work with the authors to submit a manuscript describing trial results within 12 months after the last data become available, which may take up to several months after the last subject visit in some cases such as vaccine trials. However, manuscript submission timelines may be extended on OTC trials. For trials intended for pediatric-related regulatory filings, the investigator agrees to delay publication of the trial results until the Sponsor notifies the investigator that all relevant regulatory authority decisions on the trial drug have been made with regard to pediatric-related regulatory filings. Merck will post a synopsis of trial results for approved products on www.clinicaltrials.gov by 12 months after the last subject's last visit for the primary outcome, 12 months after the decision to discontinue development, or product marketing (dispensed, administered, delivered or promoted), whichever is later.

These timelines may be extended for products that are not yet marketed, if additional time is needed for analysis, to protect intellectual property, or to comply with confidentiality agreements with other parties. Authors of the primary results manuscript will be provided the complete results from the Clinical Study Report, subject to the confidentiality agreement. When a manuscript is submitted to a biomedical journal, the Sponsor's policy is to also include the protocol and statistical analysis plan to facilitate the peer and editorial review of the manuscript. If the manuscript is subsequently accepted for publication, the Sponsor will allow the journal, if it so desires, to post on its website the key sections of the protocol that are relevant to evaluating the trial, specifically those sections describing the trial objectives and hypotheses, the subject inclusion and exclusion criteria, the trial design and procedures, the efficacy and safety measures, the statistical analysis plan, and any amendments relating to those sections. The Sponsor reserves the right to redact proprietary information.

For multicenter trials, subsequent to the multicenter publication (or after public disclosure of the results online at www.clinicaltrials.gov if a multicenter manuscript is not planned), an investigator and his/her colleagues may publish their data independently. In most cases, publication of individual trial site data does not add value to complete multicenter results, due to statistical concerns. In rare cases, publication of single trial site data prior to the main paper may be of value. Limitations of single trial site observations in a multicenter trial should always be described in such a manuscript.

Authorship credit should be based on 1) substantial contributions to conception and design, or acquisition of data, or analysis and interpretation of data; 2) drafting the article or revising it critically for important intellectual content; and 3) final approval of the version to be published. Authors must meet conditions 1, 2 and 3. Significant contributions to trial execution may also be taken into account to determine authorship, provided that contributions have also been made to all three of the preceding authorship criteria. Although publication planning may begin before conducting the trial, final decisions on authorship and the order of authors' names will be made based on participation and actual contributions to the trial and writing, as discussed above. The first author is responsible for defending the integrity of the data, method(s) of data analysis and the scientific content of the manuscript.

The Sponsor must have the opportunity to review all proposed abstracts, manuscripts or presentations regarding this trial 45 days prior to submission for publication/presentation. Any information identified by the Sponsor as confidential must be deleted prior to submission; this confidentiality does not include efficacy and safety results. Sponsor review can be expedited to meet publication timelines.

11.0 LIST OF REFERENCES

1. Disis ML. Immune regulation of cancer. J Clin Oncol 2010; 28(29):4531-8.

2. Dong H, Strome SE, Salomao DR, Tamura H, Hirano F, Flies DB, et al. Tumor-associated B7-H1 promotes T-cell apoptosis: a potential mechanism of immune evasion. Nat Med 2002;8(8):793-800.

3. Sharpe AH, Freeman GJ. The B7-CD28 superfamily. Nature 2002;2:116-26.

4. Brown JA, Dorfman DM, Ma F-R, Sullivan EL, Munoz O, Wood CR, et al. Blockade of programmed death-1 ligands on dendritic cells enhances T cell activation and cytokine production. J Immunol 2003;170:1257-66.

5. Francisco LM, Sage PT, Sharpe AH. The PD-1 pathway in tolerance and autoimmunity. Immunol Rev 2010;236:219-42.

6. Thompson RH, Dong H, Lohse CM, Leibovich BC, Blute ML, Cheville JC, et al. PD-1 expressed by tumor-infiltrating immune cells and is associated with poor outcome for patients with renal cell carcinoma. Clin Caner Res 2007;13(6):1757-61.

7. Talmadge JE, Donkor M, Scholar E. Inflammatory cell infiltration of tumors: Jekyll or Hyde. Cancer Metastasis Rev 2007;26:373-400.

8. Usubütün A, Ayhan A, Uygur MC, Ozen H, Toklu C, Ruacan S. Prognostic factors in renal cell carcinoma. J Exp Clin Cancer Res 1998;17(1):77-81.

9. Al-Shibli KI, Donnem T, Al-Saad S, Persson M, Bremnes RM, Busund L-T. Prognostic effect of epithelial and stromal symphocyte infiltration in non-small cell lung cancer. Clin Cancer Res 2008;14(16):5220-7.

10. Deschoolmeester V, Baay M, Van Marck E, Weyler J, Vermeulen p, Lardon F, et al. Tumor infiltrating lymphocytes: an intriguing player in the survival of colorectal cancer patients. BMC Immunol 2010;11:19.

11. Diez M, Pollán M, Enriquez JM, Dominguez P, Santana A, Tobaruela E, et al. Histopathologic prognostic score in colorectal adenocarcinomas. Anticancer Res 1998;18:689-94.

12. Galon J, Costes A, Sanchez-Cabo F, Kirilovsky A, Mlecnik B, Lagorce-Pagès C, et al. Type, density, and location of immune cells within human colorectal tumors predict clinical outcome. Science 2006;313:1960-4.

13. Hiraoka N. Tumor-infiltrating lymphocytes and hepatocellular carcinoma: molecular biology. Int J Clin Oncol 2010;15:544-51.

14. Nobili C, Degrate L, Caprotti R, Franciosi C, Leone BE, Trezzi R, et al. Prolonged survival of a patient affected by pancreatic adenocarcinoma with massive lymphocyte and dendritic cell infiltration after interleukin-2 immunotherapy. Report of a case. Tumori 2008;94:426-30.

15. Hodi FS, Dranoff G. The biologic importance of tumor-infiltrating lymphocytes. J Cutan Pathol 2010;37(Suppl 1):48-53.

16. Kloor M. Lymphocyte infiltration and prognosis in colorectal cancer. Lancet 2009;10:840-841.

17. Hillen F, Baeten CIM, van de Winkel A, Creytens D, van der Schaft DWJ, Winnepenninckx V, et al. Leukocyte infiltration and tumor cell plasticity are parameters of aggressiveness in primary cutaneous melanoma. Cancer Immunol Immunother 2008;57:97-106.

18. Lee HE, Chae SW, Lee YJ, Kim MA, Lee HS, Lee BL, et al. Prognostic implications of type and density of tumour-infiltrating lymphocytes in gastric cancer. Br J Cancer 2008;99(10):1704-11.

19. Leffers N, Gooden MJM, de Jong RA, Hoogeboom B-N, ten Hoor KA, Hollema H, et al. Prognostic significance of tumor-infiltrating T-lymphocytes in primary and metastatic lesions of advanced stage ovarian cancer. Cancer Immunol Immunother 2009;58:449-59.

20. Nishimura H, Honjo T, Minato N. Facilitation of β selection and modification of positive selection in the thymus of PD-1-deficient mice. J Exp Med 2000;191(5):891-7.

21. Liotta F, Gacci M, Frosali F, Querci V, Vittori G, Lapini A, et al. Frequency of regulatory T cells in peripheral blood and in tumour-infiltrating lymphocytes correlates with poor prognosis in renal cell carcinoma. BJU Intern 2010;107:1500-6.

22. Ropponen KM, Eskelinen MJ, Lipponen PK, Alhava E, Kosma V-M. Prognostic value of tumour-infiltrating lynmphocytes (TILs) in colorectal cancer. J Pathol 1997;182:318-24.

23. Dudley ME, Wunderlich JR, Yang JC, Sherry RM, Topalian SL, Restifo NP, et al. Adoptive cell transfer therapy following non-myeloablative but lymphodepleting chemotherapy for the treatment of patients with refractory metastatic melanoma. J Clin Oncol 2005;23(10):2346-57.

24. Hunder NN, Wallen H, Cao J, Hendricks DW, Reilly JZ, Rodmyre R, et al. Treatment of metastatic melanoma with autologous CD4+ T cells against NY-ESO-1. N Engl J Med 2008;358(25):2698-703.

25. Ölcher M, Braun M, Friedrichs N, Rudlowski C, Bercht E, Fimmers R, et al. Foxp3+ cell infiltration and granzyme B+/Foxp3+ cell ratio are associated with outcome in neoadjuvant chemotherapy-treated ovarian carcinoma. Cancer Immunol Immunother 2010;59:909-19.

26. Okazaki T, Maeda A, Nishimura H, Kurosaki T, Honjo T. PD-1 immunoreceptor inhibits B cell receptor-mediated signaling by recruiting src homology 2-domain-containing tyrosine phosphatase 2 to phosphotyrosine. PNAS 2001;98(24):13866-71.

27. Greenwald RJ, Freeman GJ, Sharpe AH. The B7 family revisted. Annu Rev Immunol 2005;23:515-48.

28. Kang J, Lee S, Lim D, Park K, Oh S, Kwon H, et al. Salvage chemotherapy for pretreated gastric cancer: a randomized Phase III trial comparing chemotherapy plus best supportive care with best supportive care alone. J Clin Oncol 2012; 30(13): 1513-18. .

29. Sym SJ, Ryu MH, Lee JL, Change HM, Kim TW, Lee SS, et al. Salvage chemotherapy with biweekly irinotecan, plus 5-fluorouracil and leucovorin in patients with advanced gastric cancer previously treated with fluoropyrimidine, platinum, and taxane. Am J Clin Oncol. 2008; 31(2):151-6.

30. Topalian SL, Hodi FS, Brahmer JR, Gettinger SN, Smith DC, McDermott DF, et al. Safety, activity, and immune correlates of Anti-PD-1 antibody in cancer. N Engl J Med 2012:doi: 10.1056/NEJMoa1200690.

31. Lyford-Pike S, Peng S, Young GD, et al. Evidence for a role of the PD-1:PD-L1 pathway in immune resistance of HPV-associated head and neck squamous cell carcinoma. Cancer Res 2013;73(2): Published online January 3.

32. Eisenhauer EA, Therasse P, Bogaerts J, Schwartz LH, Sargent R, Ford R, et al. New response evaluation criteria in solid tumors: Revised RECIST guideline (version 1.1). Eur J Cancer 2009;45(2):228-47.

33. Wolchok JD, Hoos A, O'Day S, Weber JS, Hamid O, Lebbe C, et al. Guidelines for the evaluation of immune therapy activity in solid tumors: immune-related response criteria. Clin Cancer Res 2009;15(23):7412-20.

34. Blum JL, Jones SE, Buzdar AU, LoRusso PM, et al. Multicenter Phase II Study of Capecitabine in Paclitaxel-Refractory Metastatic Breast Cancer. J Clin Oncol 17: 485-493, 1999

35. Martin M, Ruiz A, Munoz M, Balil A, Garcia-Mata J, Calvo L, et al. Gemcitabine plus vinorelbine monotherapy in patients with metastatic breast cancer previously treated with anthracyclines and taxanes: final results of the phase III Spanish Breast Cancer Research Group (GEICAM) trial. Lancet Oncol. 2007;8(3):219.

36. Sledge GW, Neuberg D, Bernardo P, Ingle JN, Martino S, et al. Phase III Trial of Doxorubicin, Paclitaxel, and the Combination of Doxorubicin and Paclitaxel as Front-Line Chemotherapy for Metastatic Breast Cancer: An Intergroup Trial (E1193). J Clin Oncol. 2003;21(4):588.

37. Stewart JSW, Ezra E.W. Cohen EEW, Licitra L, Van Herpen CML, et al. Phase III Study of Gefitinib Compared With Intravenous Methotrexate for Recurrent Squamous Cell Carcinoma of the Head and Neck. J Clin Oncol. 2009;27(11):1864.

38. Vermorken JB, Trigo J, Hitt R, Koralewski P, Diaz-Rubio E, et al. Open-Label, Uncontrolled, Multicenter Phase II Study to Evaluate the Efficacy and Toxicity of Cetuximab As a Single Agent in Patients With Recurrent and/or Metastatic Squamous Cell Carcinoma of the Head and Neck Who Failed to Respond to Platinum-Based Therapy. J Clin Oncol. 2007;25(16):2171.

39. Bellmunt J, Théodore C, Demkov T, Komyakov B, Sengelov L, et al. Phase III Trial of Vinflunine Plus Best Supportive Care Compared With Best Supportive Care Alone After a Platinum-Containing Regimen in Patients With Advanced Transitional Cell Carcinoma of the Urothelial Tract. J Clin Oncol. 2009;27(27):4454

40. Sweeney CJ, Roth BJ, Kabbinavar FF, Vaughn DJ, Arning M, et al. Phase II Study of Pemetrexed for Second-Line Treatment of Transitional Cell Cancer of the Urothelium. J Clin Oncol. 2006;24(21):3451

41. Clopper C and Pearson ES. The use of confidence or fiducial limits illustrated in the case of the binomial. Biometrika 1934;26:404-413.

42. Garon E. Preliminary clinical safety and activity of MK-3475 monotherapy for the treatment of previously treated patients with non-small cell lung cancer. Presented at: IASLC 15th World Conference on Lung Cancer; October 2013; Sydney, Australia. MO18.02.

43. Robert, C., et. al, Updated Clinical efficacy and safety of MK-3475 (Anti-PD-1 monoclonal antibody) in advanced melanoma. Society for Melanoma Research 2013 Congress; November 17-20, 2013; Philadelphia, PA, USA. Pigment Cell & Melanoma Research: John Wiley & Sons Ltd; Volume 26, Issue 6, pages 932–1019, November 2013.
